# The Origin of the Kinetic Isotope Effect and Mechanism of H/D Exchange in Radical C–C Bond Formation Catalyzed by Benzylsuccinate Synthase: Experiments and Microkinetic Modeling

**DOI:** 10.1021/acs.jpcb.6c01533

**Published:** 2026-08-28

**Authors:** Maciej Szaleniec, Gabriela Oleksy, Ivana Aleksic, Kai Krämer, Tomasz Borowski, Johann Heider

**Affiliations:** † Jerzy Haber Institute of Catalysis and Surface Chemistry, Polish Academy of Sciences, Kraków 30-239, Poland; ‡ Department of Biology, Laboratory for Microbial Biochemistry, 9377 Philipps University Marburg, Marburg 35043, Germany; § SYNMIKRO Research Center, Marburg 35043, Germany

## Abstract

Fumarate-adding enzymes (FAE) are a subset of the glycyl radical enzyme superfamily involved in anaerobic hydrocarbon degradation. Benzylsuccinate synthase (BSS) catalyzes the enantiospecific formation of (*R*)-benzylsuccinate from toluene and fumarate, initiating anaerobic toluene degradation. In this paper, we present the first microkinetic analysis of the full reaction, predicting kinetic isotope effects in the range of 2.587–2.604, close to the experimentally observed value (KIE^exp^= 2.13 ± 0.1). Our experiments confirmed that KIE values are lower in direct assays with substrate-saturated enzyme, relative to the values obtained for competitive kinetic isotope effects ^*D*^(*V*/*K*) via substrate fractionation (3.69 ± 0.16). We postulate that this apparent KIE suppression originates from preferential binding of unlabeled versus labeled substrates to the enzyme, as well as from the rates of product release. We also show that tunneling effects have only minor influence on the observed KIE and ^*D*^(*V*/*K*) values, although they have been estimated to potentially accelerate the overall reaction rate by approximately 17%. On the other hand, inclusion in the kinetic equation of the barrier recrossing effect, jointly with tunneling correction can result in a lowering of predicted KIE values to the range of 2.38–2.43. Furthermore, we analyze a slowly occurring, experimentally observed H/D exchange process in the product during incubation in D_2_O, confirming partial reversibility of the reaction. We estimate the rate of this H/D exchange and propose a potential mechanism. Our study contributes to elucidating the processes catalyzed by BSS and its role in the bioremediation of hydrocarbon pollutants.

## Graphical abstract

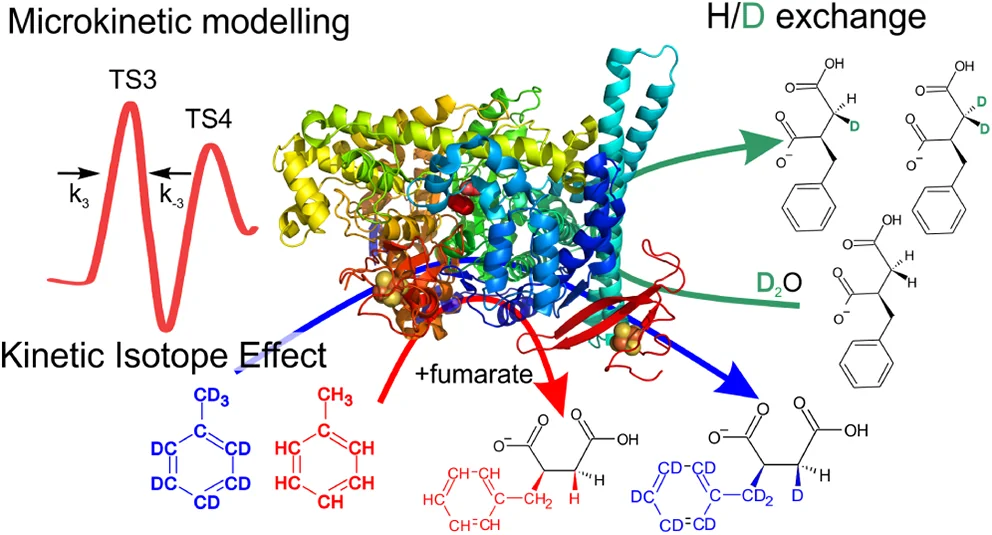

## Introduction

The kinetic isotope effect is one of the most direct ways of validating the mechanistic hypothesis for a reaction. It is often quite easy to establish experimentally, and several protocols are available to eliminate sources of measurement error. This is especially true for competitive kinetic isotope effects, where both isotopologues are present in the reaction mixture and errors in the concentrations of the catalyst or reagents cancel out. Furthermore, the calculation of KIE values is also very robust due to the highly local nature of the KIE (restricted to a few bonds away from the transferred atom), which results in sensible predictions, even when relatively small models of reaction systems are used. Even in the pre-quantum-chemistry era, many studies showed that agreement between predicted and experimental values is a valuable tool for validating or falsifying a mechanistic hypothesis.

In this paper, we study the multistep reaction mechanism of (*R*)-benzylsuccinate synthesis from toluene and fumarate by benzylsuccinate synthase (BSS), which has been intensively investigated and validated at various levels of theory over the past few years.^1–7^ In particular, we explore the extent to which microkinetic analysis combined with QM/MM modeling can explain two strange experimental observations in the kinetic results obtained for the reaction. First, experimental studies, reported previously^2^ and in this paper, showed that values of direct KIEs (i.e., measured at enzyme saturation with substrates) for BSS are significantly lower than those obtained in competitive experiments (i.e., reflecting kinetic isotope fractionation when both substrates are present in the reaction mixture). Second, an unexpected double-deuterated H/D exchange product was produced when the enzyme was incubated with (*R*)-benzylsuccinate in the presence of D_2_O.^5^ We show that our approach can be used for a deeper study of any complex reaction for which one can collect steady-state kinetic data, as well as to conduct reliable mechanistic calculations.

Fumarate-adding enzymes (FAEs) comprise a subset of the glycyl radical enzyme (GRE) superfamily^8,9^ and are involved in anaerobic degradation pathways of hydrocarbons and similar substrates, catalyzing the addition of chemically inert alkyl carbon atoms to the double bond of a fumarate cosubstrate.^10–13^ The first recognized FAE was benzylsuccinate synthase (BSS),^14,15^ which enantiospecifically forms (*R*)-benzylsuccinate from toluene and fumarate^15–17^ and initiates anaerobic toluene degradation in all known organisms capable of this trait. Very similar initial reactions were also reported for the anaerobic degradation of many other hydrocarbons.^18–27^ The discovery of both the characteristic succinate adducts and the genes coding for the respective FAEs in field studies of contaminated sediments and aquifers indicated that these enzymes are widespread in nature and contribute an important role to bioremediation.^28–33^

The mechanism of BSS (Figure 1A) has been investigated for many years,^10,13,34^ and recently we analyzed it in detail using a QM/MM approach, which for the first time provided a step-by-step mechanistic proposal in the full protein context.^6^ The enzyme first needs to be activated to the relatively stable glycyl radical state by a separate S-adenosylmethionine-dependent activating enzyme,^35^ but it appears to be relatively unreactive in this state. Until recently, it was assumed that the binding of the substrates (fumarate and toluene) triggers conformational changes that close the active site^3,36^ and initiate the reaction by hydrogen atom transfer (HAT) from the conserved Cys493 of the active site to the glycyl radical at Gly829. However, a recent study by Anas et al.^37^ suggests that the accessory subunit BSSβ instead promotes thiyl radical formation on Cys493, even in the absence of substrates. In any case, removal of a hydrogen atom from Cys493 generates a reactive thiyl radical, unlocks Cys493 from facing toward Gly829, and increases the chance that the thiyl radical turns toward the toluene bound in the active site, where it abstracts a hydrogen atom from the methyl group. The generated benzyl radical intermediate attacks the double bond of the bound fumarate, forming a new C–C bond in a benzylsuccinyl product radical. The attack proceeds regioselectively on the less sterically hindered C atom of the double bond, resulting in the formation of an (*R*)-benzylsuccinyl radical in close proximity to Cys493. As a result, the product radical can re-abstract the hydrogen atom from the Cys493 side chain and subsequently the product can be released from the active site. This step is reversible, as during incubation of the product with the enzyme in D_2_O, H/D exchange occurs (Figure 1B), yielding d_1_-benzylsuccinate and, to a smaller extent also d_2_-benzylsuccinate.^5^ Our calculations indicate that a conformational shift of the enzyme occurs after the release of the product, which makes the final transition from the thiyl to the glycyl radical state more favorable. The proposed mechanism is fully in accordance with the experimentally determined (*R*)-enantiospecificity of the reaction,^16^ the preferential return of the abstracted hydrogen in a *syn* addition,^38^ the inversion of the methyl group of toluene during the reaction,^39^ and a specific preference for toluene addition to the distal C atom of the double bond of fumarate to enable the product radical to retrieve the hydrogen from Cys493.^36^

**Figure 1:**
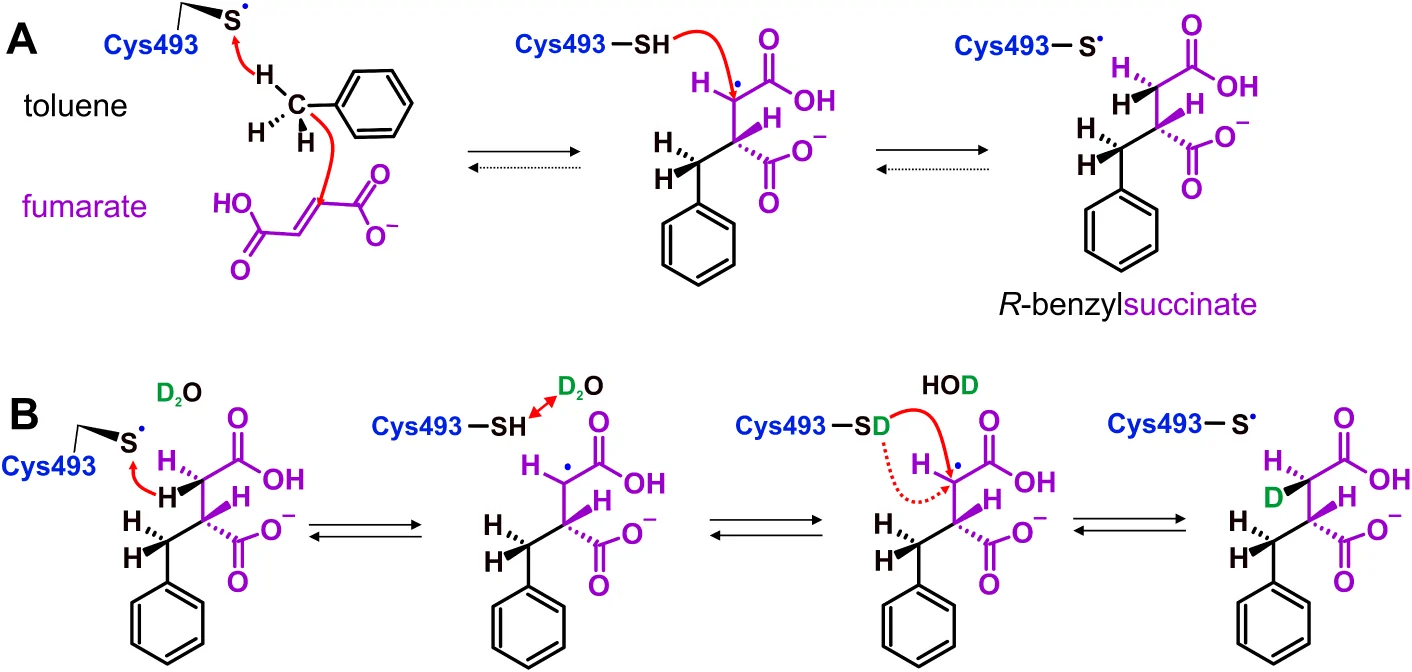
Principal catalytic mechanism proposed for BSS: A) synthesis of (*R*)-benzylsuccinate from toluene and fumarate, B) H/D exchange in the product during incubation in D_2_O.

In this study, we tested the mechanistic hypothesis by comparing theoretically predicted kinetic isotope effects with experimental values. In contrast to previous attempts, we estimated kinetic rates for all internal elementary steps and derived a kinetic equation involving substrate binding and product release steps. It should also be emphasized that this represents the first kinetic model of BSS reactivity based on calculations performed for the full protein, taking into consideration the conformational constraints imposed on reagent orientation by the geometry of the active site. We demonstrate that such an approach, which can be applied to the study of any reaction, enables a better understanding of factors influencing the experimentally observed kinetic phenomena.

## Methods

### BSS Production in *Aromatoleum* *Sp*

Cell-free extract from toluene-grown *Aromatoleum* *sp.* was prepared as described in Szaleniec et al.^5^ Growth conditions, DNA isolation, gene sequencing, and bioinformatic analysis are described in the SI.

### Kinetic Characterization

The kinetic parameters of BSS activity with toluene were determined under saturation with the second substrate, fumarate. 100 μL of the cell-free extract (100 mg protein/mL) was diluted in 0.83 mL of TEA-HCl buffer (pH 7.8) and incubated with 50 μL of sodium fumarate, added from a 100 mM aqueous stock, to a final, saturating concentration of 5 mM. The activity test was initiated by adding toluene from a 167 mM stock solution in isopropanol to obtain final concentrations of 0.3, 0.6, 0.9, 1.2, 2, and 3 mM. The reaction volume was adjusted to 1 mL with isopropanol to ensure equal organic content in each experiment (2%). Samples were taken at *t*_0_ and every 5 min for the first 20 min of the reactions. The reactions were stopped by mixing the sample in a 1:1 ratio with acetonitrile, and spiked with 0.367 mM phenylacetic acid, which served as an internal standard. Samples were centrifuged (8000 × *g*, 20 min) to remove the residual protein content and transferred to HPLC vials. BSS activity was analyzed by measuring the product concentration with LC-MS/MS. Experiments were performed in triplicate. The obtained data were fitted with the Michaelis–Menten model in OriginPro 2021.

### Kinetic Isotope Effect

The kinetic isotope effect (KIE) was measured using two methods: i.e., a direct method, in which kinetic assays are conducted independently with labeled and unlabeled substrates, and a competitive method, in which the reaction is carried out with a mixture of both isotopologues of the substrate. The value of the KIE is the ratio of kinetic constants obtained from zero-order kinetics measured under enzyme-saturated conditions with unlabeled and labeled substrates (eq 1).

1 $$KIE=\frac{k^{H}}{k^{D}}$$

Meanwhile, the competitive kinetic isotope effect (VK)D is by definition, the ratio of the secondary kinetic rate (*V*/*K*) measured for the light and heavy substrates:

2 (VK)D=(VHKmH)(VDKmD)

The experimental values were compared with the kinetic isotope effects predicted on the basis of microkinetic constants calculated for the reaction pathway involving toluene and d_8_-toluene.

### Direct Kinetic Isotope Effect

Direct KIE was measured by conducting separate assays for unlabeled and isotopically labeled d_8_-toluene. The reaction mixture comprised 0.2 mL of cell-free extract (39 mg/mL) buffered with 0.73 mL of 20 mM TEA/HCl, pH 7.8, 50 μL of a 100 mM aqueous solution of sodium fumarate (final concentration of 5 mM) and 3 mM toluene (8.2 μL of a 364.5 mM stock solution in isopropanol) or d_8_-toluene (8.4 μL of a 358.2 mM stock solution in isopropanol). The reactions were conducted anaerobically at 30 °C. Samples of 150 μL were collected at 0, 5, 10, 15, and 20 min, and the reaction was stopped by the addition of acetonitrile in a 1:1 (v/v) ratio. The samples were then centrifuged (8000 × *g*, 20 min) to remove the precipitated protein, and the supernatants were analyzed by LC-DAD. All experiments were carried out in triplicate.

The catalytic activities of BSS with toluene and d_8_-toluene were determined by linear regression of the product concentration over time for the first 20 min of the reaction using OriginPro 2021. The KIE was calculated as the ratio of the average enzyme activity recorded with toluene to that recorded with d_8_-toluene.

### Competitive Kinetic Isotope Effect

The reaction mixture, comprising 0.2 mL of cell-free extract (39.8 mg/mL), 0.73 mL of 20 mM TEA/HCl buffer, pH 7.8, and 50 μL of 100 mM sodium fumarate (final concentration of 5 mM), was assembled under anaerobic conditions and incubated at 30 °C in a thermoblock for 2 min before reaction initiation. The reactions were initiated by the addition of a 3 mM toluene/d_8_-toluene mixture dissolved in isopropanol, resulting in final concentrations of 1.5 mM for both toluene and d_8_-toluene. The isopropanol content in each reaction mixture was adjusted to 2%. Samples of 150 μL were collected at 0, 5, 10, 15, and 20 min, then mixed in a 1:1 ratio with acetonitrile to stop the reaction. They were then further processed as described above. All experiments were carried out in triplicate.

The signal ratio of lighter and heavier products (m/z 207→163 vs m/z 215→171) from MS/MS was combined with quantitative analysis from DAD. The competitive KIE on (VK)D was estimated by following the changes in benzylsuccinate and d_8_-benzylsuccinate molar fractions over the course of the reaction, up to 30% conversion of the lighter isotopologue, according to the formula:

3 (VK)D=k1k2=log(1−x1)log(1−x2)

Molar fractions of heavier (*x*_2_) and lighter (*x*_1_) products were plotted in OriginPro 2021 (Figure 5B). (VK)D was obtained by nonlinear regression to *x*_1_(*x*_2_), following the equation:^40^

4 $$x_{1}=1-{(1-x_{2})}^{\frac{k_{1}}{k_{2}}}$$

### UHPLC-DAD-MS/MS

Samples were analyzed by UHPLC (Agilent 1260) equipped with DAD and 6460 Triple Quad MS detectors, according to procedures described in ref. 5. In short, separations of direct KIE samples were conducted on a ZORBAX 300SB -C18 column (RRHD, 2.1 x 50 mm, 1.8 μm, Agilent) at 30 °C and a flow rate of 0.4 mL/min with a gradient in H_2_O/ACN/0.01% HCOOH mobile phase (see SI), using 2 μL injection volume and 210 nm as the detection wavelength.

The samples from competitive KIE were separated using the same chromatographic program, and the MS signal was detected in Multiple Reaction Monitoring mode (MRM), using a jet stream ESI ion source in negative polarity mode, following the transitions of the parent [M – H]^−^ ions at m/z 207 and 215, respectively for benzylsuccinate and d_8_-benzylsuccinate and the fragmentation to [M – H_2_O – H]^−^ quantifier ions at m/z 189 or 197, as well as [M – CO_2_ – H]^−^ qualifier ions (m/z 163 or 171, respectively ). Additionally, the signal of d_7_-benzylsuccinate (m/z 214), i.e., the product of H/D exchange occurring in H_2_O during the reaction, was also analyzed (fragmentation ions at m/z 188 and 196) and added to the abundance of d_8_-benzylsuccinate (see Tables S1–S3 for method parameters). Additionally, the 135→91 m/z transition of phenylacetic acid, used as an internal standard, was monitored to correct for sample preparation errors. The data from the MS experiment were used to determine the ratio of labeled to unlabeled products, while the total product concentration was determined by analysis of undiluted samples with the DAD detector (Figure S1).

### BSS Model Preparation

The initial structure of the α subunit of BSS_T1_ in complex with monoprotonated fumarate and toluene was obtained from the crystal structures (PDB codes: 5BWD, 5BWE)^41^ as described previously by Salii et al.^3^ and Szaleniec et al.^5,6^

### MD and QM/MM Modeling of the Reaction Mechanism

As described in our previous report,^6^ prior to modeling MD simulation of the BSS model E:S complex was conducted using the AMBER ff03 force field^42^ in AMBER 18 ^43^ according to the previously described protocol.^3^ The MD trajectory was analyzed by clustering, providing initial geometries for the QM:MM studies.

### QM/MM

The reaction mechanism was studied using the QM/MM method with the Gaussian 16, Revision C.01 program.^44^ The detailed methodology was described recently in ref. 6. In short, the potential energy surface was explored in consecutive steps of the reaction using a partially constrained model of the catalytic subunit (beyond a 15 Å radius from Cys493). The geometry optimization and vibrational corrections were calculated at the B3LYP/6-31g­(d,p):AMBER level of theory for the smaller QM part (QM1), which comprised the Cys493 and Gly829 residues with adjacent fragments of the main chain, Gln707, fumarate, and toluene (see Figure S2a). The energies of the obtained geometries of stationary states were corrected by single-point calculations at the B3LYP/6-311g+(2d,2p):AMBER level of theory with electronic embedding and Grimme’s D3 dispersion interactions^45^ with a larger QM part (QM2), which expanded the QM1 part to include all residues and solvent molecules within a 3 Å radius of Cys493, toluene, and fumarate (Figure S2b).

To estimate the intrinsic kinetic isotope effects, we used d_8_-toluene. The electronic energies were corrected with thermal energy corrections calculated at the B3LYP/6-31G­(d, p):AMBER level of theory at 303 K and 1 atm, using a 0.9806 scaling factor according to the B3LYP/6-31g­(d) correction calculated by Scott and Radom.^46^ The thermal energy correction was applied to account for the experimental temperature. We decided not to use Gibbs free energy corrections due to the potential bias in entropy estimation from vibrational analysis in the partially constrained model. All data for the energy profiles and vibrational corrections that account for isotopologues of the substrate are provided in Tables S4–S7.

### Kinetic Rate Estimations

All kinetic constants were calculated according to the Eyring transition-state theory equation:

5 $$k_{i}=\left(\frac{k_{B}T}{h}\right)\exp\left(\frac{-\Delta E_{\mathrm{therm}}^{‡}}{RT}\right)$$

where *k*_B_ is the Boltzmann constant, *h* is the Planck constant, *R* is the gas constant, *T* is 303 K, Δ*E* ^‡^_therm_ is the thermal energy calculated for 303 K, and the transmission coefficient is assumed to be unity. It should be noted that the Eyring equation introduces a partition function and applies the Gibbs free energy correction to the electronic energy (Δ*G* ^‡^). However, as our QM:MM model has only 2901 out of 14046 atoms subjected to geometry optimization, the estimation of entropy based on vibrational analysis may introduce significant errors. Therefore, in our analysis, we used thermal energy instead (Δ*E* ^‡^_therm_), which only partially describes the partition function. On the other hand, kinetic rates derived using Δ*E* ^‡^_therm_ were previously shown to provide an unbiased estimate of the kinetic isotope effects.^47^

To account for the tunneling effect that may be involved in the H atom transfer, the obtained rates were corrected by Wigner’s tunneling corrections Γ­(*T*)^48^ according to eqs 6–7:

6 $$k_{i}^{T}=\Gamma \left(T\right)k_{i}$$

7 $$\Gamma \left(T\right)=1+\frac{1}{24}{(\frac{hIm(\nu^{‡})}{k_{b}T})}^{2}$$

where *Im*(ν^‡^) is the imaginary vibrational frequency (Hz) associated with the transition state.

To account for the recrossing effect, the kinetic constants calculated by eq 5 were corrected using Wigner’s tunneling factor and the estimated transmission coefficient κ:

8 $$k_{i}^{\mathrm{recross}}=\kappa \cdot \Gamma \left(T\right)\cdot k_{i}$$

The calculated kinetic constants were used to estimate the reaction rate based on the equation derived according to the King–Altman algorithm^49^ (see the SI Methods section). The derivation was verified by an algebraic method conducted in a Jupyter notebook using the SymPy Python library.^50^

Estimation of the predicted KIE error was based on the ratio of derived kinetic equations (eq 10 and S10) calculated for unlabeled and labeled toluene, while the value of ^*D*^(*V*/*K*) was estimated from the corresponding values calculated using eq S12. The rates of H/D exchange were calculated based on eqs S19 and S25 for single H/D exchange or eqs S32 and S40–S41 for double exchange. The Python 3 script, saved as a Jupyter notebook, is provided in the SI.

## Results

In a recent paper, we analyzed a number of mechanistic variants that describe the reaction catalyzed by benzylsuccinate synthase.^6^ In Figure 2, we present all stages of the proposed detailed reaction mechanism.^6^ In contrast to earlier modeling studies, we propose that the product is released from the active site immediately after its formation (E:P), while the radical remains on Cys493. This triggers a conformational change of the protein, resulting in destabilization of the thiyl radical state relative to the glycyl radical state. As a result, a hydrogen atom is transferred quite easily from Gly829 to Cys493 (barrier of 2.3 kJ mol^–1^). In the alternative scenario, the HAT would occur with the benzylsuccinate product still bound in the active site. However, the calculation predicts that, for such a situation, the thiyl radical state is more stable than the glycyl radical state, resulting in a very high energy barrier for the transfer (96.2 kJ mol^–1^), which makes it highly improbable.^5^

**Figure 2:**
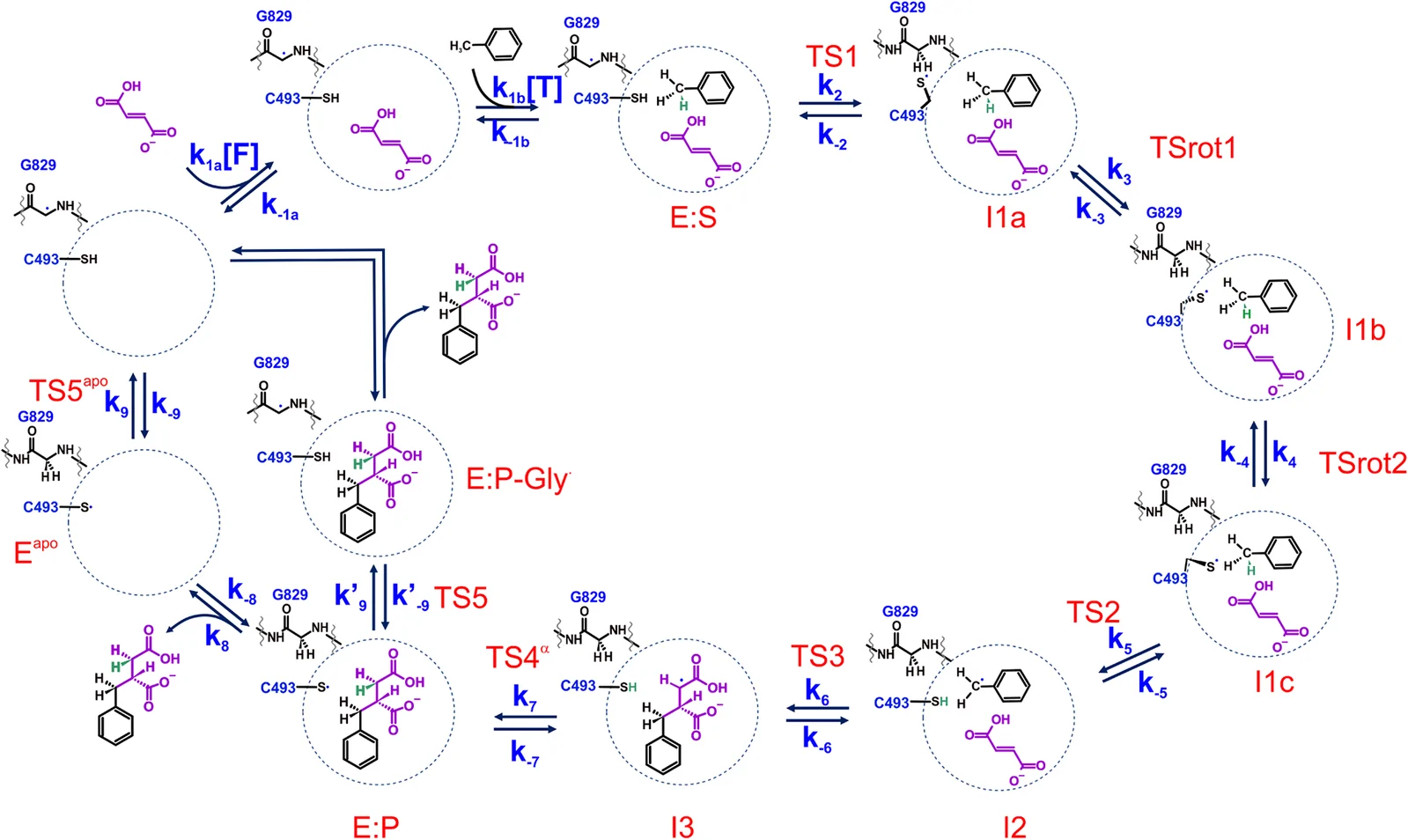
The detailed reaction mechanism proposed for (*R*)-benzylsuccinate formation is described according to published studies published in refs 6 and 5 [F] and [T] stand for fumarate and toluene concentrations, and E:S and E:P are the enzyme-substrate and enzyme-product complexes, respectively. TS indicates the various transition states, TSrot indicates transition states for rotation of certain amino acid residues, and I indicates the various intermediate states.

The energy profile analysis (Tables S4–S5) shows that all reaction barriers, from radical transfer at the active site to product formation, are below 60 kJ mol^–1^ with respect to E:S, and the highest barrier is associated with radical quenching of the benzylsuccinyl radical (TS4, 57 kJ mol^–1^) i.e., with a transfer of a radical H atom from the Cys-SH group to the benzylsuccinyl radical, which removes the radical from the product and creates a cysteinyl radical instead. However, the highest overall energy difference is between I1 (E:S complex with activated Cys493) and the barrier responsible for C–C bond formation (TS3), which amounts to 87 kJ mol^–1^ (Figure 3).

**Figure 3:**
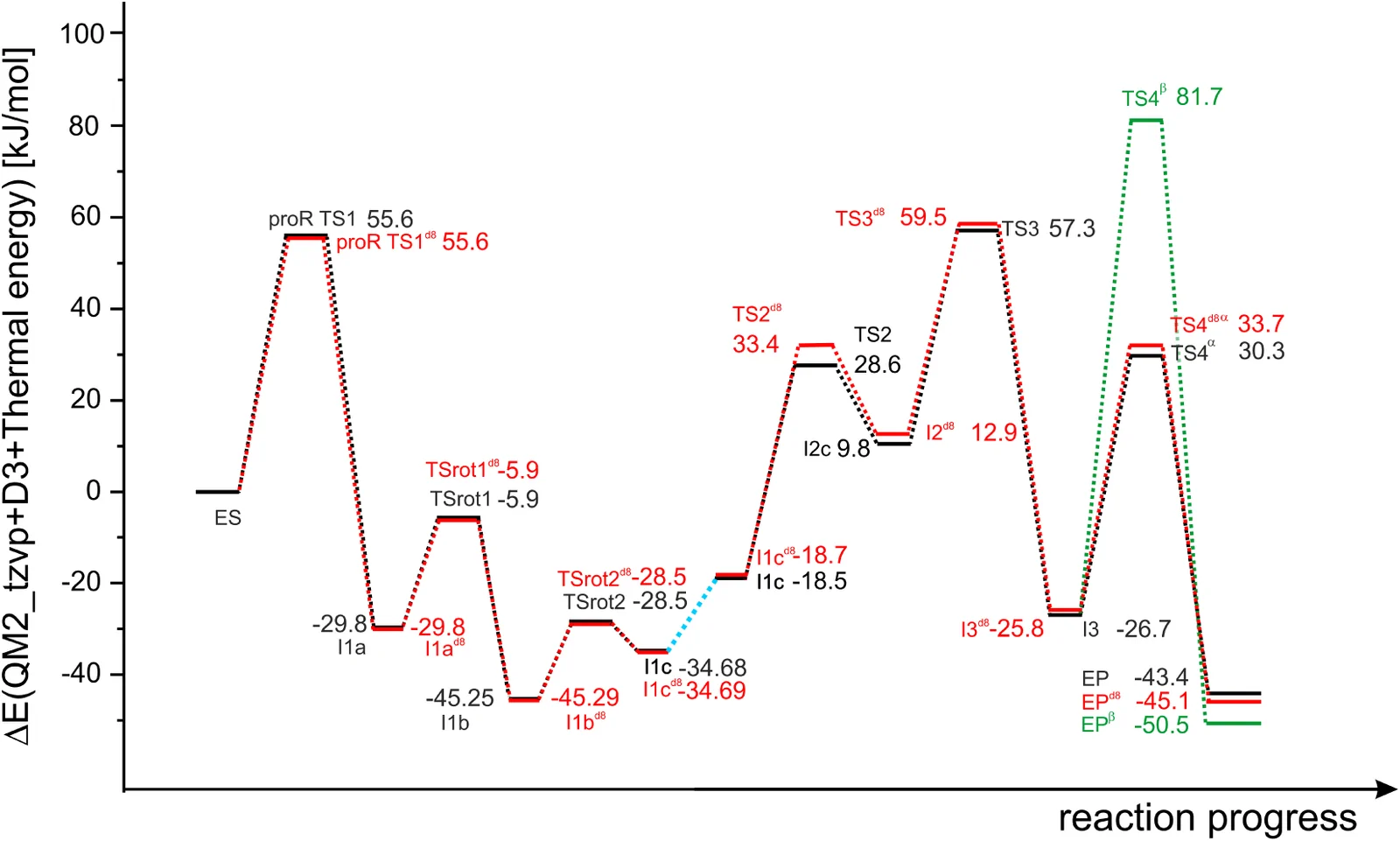
Energy diagram for the mechanism. The energy profiles have been calculated for toluene (black profiles and numbers), for d^8^-toluene (red profiles and numbers), and for an alternative radical benzylsuccinate quenching pathway (green profile). The blue dotted line indicates a necessary conformational change that enables reaction progress but shifts the energy of I1c relative to that of I1a (see ref. 6).

The proposed mechanism also describes the enantioselectivity of the quenching step of the benzylsuccinyl radical (TS4). The mechanism indicates a clear kinetic preference for *syn* addition (TS4^α^) of the H atom with respect to the newly formed C–C bond, but the energy barrier for *anti*-addition is not so much higher as to completely exclude this pathway (TS4^β^). This issue is especially important for elucidating the observed H/D exchange patterns.

### Kinetic Isotope Effect

One way to test mechanistic hypotheses is to compare experimental data with the results of modeling. As the formation of benzylsuccinate from fumarate and toluene involves multiple transfers of an H atom, an experiment with deuterium-labeled toluene as the substrate could manifest a kinetic isotope effect (KIE). It was previously demonstrated that a significant KIE is observed with fully deuterated toluene, although reports were inconclusive regarding its precise value.^2,39^ However, modeling provides a means of calculating intrinsic reaction rates (i.e., for a particular molecular step of the reaction), and this can be used to estimate *k*_*cat*_. Calculation of the ratios of such rate constants, for unlabeled and labeled substrates, yields the predicted values of KIE’s that should be directly comparable to those obtained from experiments. Interestingly, such a method was previously applied in the study of the BSS mechanism by Li and Marsh,^2^, where the experiment was compared with barriers computed for a gas-phase model,^51^ but it did not take into account the influence of the rest of the enzyme beyond the active site.

We used the same approach to establish initial reaction conditions, assuming, however, that the irreversible step is the quenching of the benzylsuccinyl radical, followed by release of the product, rather than the C–C bond-formation step, as suggested by the gas-phase model.^1^ This is because quenching of the benzylsuccinyl radical is associated with the lowest rate constant (*k*_7_) of all partial steps of the mechanism in the forward direction (see Tables 1 and S5-6) when calculated with respect to the preceding stationary points. We also assumed saturation of the enzyme by the respective substrate, thus eliminating dependence on E:S complex formation (*k*_1_ and *k*_–1_) in the kinetic equation.

**Table 1:** The Kinetic Constants (*k*_*i*_) Calculated from Thermal Energy Barriers in QM/MM for the pro*R* Pathway with Toluene and d_8_-Toluene; Γ­(*T*)*k_*i*_ -Rates Corrected by Wigner’s Tunneling Correction, iKIE – Intrinsic Kinetic Isotope Effect, iKIE^t^ Intrinsic Kinetic Isotope Effect Including Wigner’s Tunneling Correction

	Δ ***E* (QM2GD3 + Thermal_En** ^**303K**^ **) [kJ/mol]**		***k***_***i***_ **[s** ^**–1**^]	Γ(*T*)*k_***i***_ **[s** ^**–1**^]	^***D***^ ***k***_***i***_ **[s** ^**–1**^]	Γ(*T*)* *^**D**^ **k***_***i***_ **[s** ^**–1**^]		
	h_8_-toluene	d_8_-toluene	h_8_-toluene	h_8_-toluene	d_8_-toluene	d_8_-toluene	iKIE	iKIE^t^
*k*_2_	55.61	55.58	1635	3104	1658	3146.7	1.0	1.0
*k*_–2_	85.39	85.38	1.20 × 10^–2^	2.29 × 10^–2^	1.21 × 10^–2^	2.29 × 10^–2^	1.0	1.0
*k*_3_	23.89	23.90	4.8 × 10^8^	4.81 × 10^8^	4.79 × 10^8^	4.80 × 10^8^	1.0	1.0
*k*_–3_	39.35	39.39	1.04 × 10^6^	1.04 × 10^6^	1.02 × 10^6^	1.02 × 10^6^	1.0	1.0
*k*_4_	16.78	16.79	8.1 × 10^9^	8.13 × 10^9^	8.05 × 10^9^	8.09 × 10^9^	1.0	1.0
*k*_–4_	6.20	6.19	5.38 × 10^11^	5.40 × 10^11^	5.42 × 10^11^	5.44 × 10^11^	1.0	1.0
*k*_5_	47.08	52.14	4.83 × 10^4^	1.01 × 10^5^	6.49 × 10^3^	1.06 × 10^4^	7.4	9.6
*k*_–5_	18.73	20.49	3.73 × 10^9^	7.83 × 10^9^	1.85 × 10^9^	3.03 × 10^9^	2.0	2.6
*k*_6_	47.47	46.54	4.15 × 10^4^	4.87 × 10^4^	6.00 × 10^4^	7.04 × 10^4^	0.7	0.7
*k*_–6_	84.04	85.31	2.06 × 10^–2^	2.42 × 10^–2^	1.24 × 10^–2^	1.46 × 10^–2^	1.7	1.7
*k*_7_	57.07	59.57	917	2324	340	637	2.7	3.6
*k*_–7_	73.79	78.86	1.2	3.0	0.16	0.30	7.5	10
*k*_9_	2.3^a^	n.d.	2.5 × 10^12^	7.78 × 10^12^	n.d.	n.d.	-	-
*k*_–9_	78.3^a^	n.d.	0.2	0.62	n.d.	n.d.		-
*k*_*9*’_	96.20	96.18	1.64 × 10^–4^	2.98 × 10^–4^	1.66 × 10^–4^	3.0 × 10^–4^	1.0	1.0
*k*_–9_^’^	54.70	54.67	2351	4256	2378	4305	1.0	1.0
Kinetic constants fitted to eq S12								
*k*_1_			4.66640 [M^–1^ s^–1^]	3.25829 [M^–1^ s^–1^]	4.6728 [M^–1^ s^–1^]	3.2638 [M^–1^ s^–1^]	0.999	0.998
*k*_–1_			1	1	1	1	1.0	1.0
*k*_8_			1.978 × 10^–2^ [s^–1^]	2.341 × 10^–2^ [s^–1^]	1.978 × 10^–2^ [s^–1^]	2.341 × 10^–2^ [s^–1^]	1.0	1.0

a Barriers calculated for the apoenzyme in Szaleniec et al.,^5^ n.d. not determined.

Assuming that quenching of the product radical is the rate-limiting step, the rate of the overall reaction should depend only on the concentration of the enzyme containing Cys493 (carrying an SH group) and the benzylsuccinyl radical [BSS-C:BS^.^]:

9 $$V=k_{5}\left[\mathrm{BSS}‐C:BS^{\cdot }\right]$$

We assume that the reverse reaction (i.e., binding of benzylsuccinate and its conversion to the radical) can be neglected in the initial stage of the reaction due to the low concentration of the available product, which results in a low probability of its rebinding. With these assumptions, we derived an equation describing the reaction velocity using the King-Altman algorithm^49^ (see SI for full derivation, eqs S1–S10). As a result, the kinetic equation eq 9 describes all internal kinetic steps of the reaction without substrate binding or product release.

10 $$\frac{V}{E_{T}}=\frac{k_{2}k_{3}k_{4}k_{5}k_{6}k_{7}}{\begin{array}{l} k_{2}k_{3}k_{4}k_{5}k_{6}+k_{2}k_{3}k_{4}k_{5}k_{7}+k_{2}k_{3}k_{4}k_{5}k_{‐6}+k_{2}k_{3}k_{4}k_{6}k_{7}+k_{2}k_{3}k_{4}k_{7}k_{‐5}+k_{2}k_{3}k_{4}k_{‐5}k_{‐6}+k_{2}k_{3}k_{5}k_{6}k_{7}\\ +k_{2}k_{3}k_{6}k_{7}k_{‐4}+k_{2}k_{3}k_{7}k_{‐4}k_{‐5}+k_{2}k_{3}k_{‐4}k_{‐5}k_{‐6}+k_{2}k_{4}k_{5}k_{6}k_{7}+k_{2}k_{5}k_{6}k_{7}k_{‐3}+k_{2}k_{6}k_{7}k_{‐3}k_{‐4}\\ +k_{2}k_{7}k_{‐3}k_{‐4}k_{‐5}+k_{2}k_{‐3}k_{‐4}k_{‐5}k_{‐6}+k_{3}k_{4}k_{5}k_{6}k_{7}+k_{4}k_{5}k_{6}k_{7}k_{‐2}+k_{5}k_{6}k_{7}k_{‐2}k_{‐3}+k_{6}k_{7}k_{‐2}k_{‐3}k_{‐4}\\ +k_{7}k_{‐2}k_{‐3}k_{‐4}k_{‐5}+k_{‐2}k_{‐3}k_{‐4}k_{‐5}k_{‐6} \end{array}}$$

Using this approach, we calculated *k*_*cat*_ using kinetic constants of elementary steps derived from our QM/MM modeling for toluene and d_8_-toluene. The predicted *k_cat_* for toluene was 7.94 × 10^–3^ s^–1^ while that for d_8_-toluene was 3.07 × 10^–3^ s^–1^, which resulted in a predicted KIE of 2.59. To estimate the margins of error of our analysis, we calculated KIE’s for assumed transition barrier deviations of ±12.96 kJ mol^–1^ (3.11 kcal mol^–1^), based on a recently estimated mean absolute deviation of barriers in HAT reactions computed with B3LYP.^52^ We varied each barrier independently of the other, changing the electronic energies for the forward and reverse steps (i.e., *k_i_* and *k*_–*i*_) by the same amount. The analysis (see Figure 4) showed that the predicted KIE varied from 2.587 to 2.604, and the predicted value was most sensitive to an increase of the barrier connected with the *k*_6_/*k*_–6_ constant (TS3), which could unmask the observed KIE if the barrier were lower. This step is associated with the formation of the C–C bond and exhibits an inverse KIE in the forward direction and a relatively low KIE in the reverse direction. As expected, increasing the barriers associated with the transfer of H/D from toluene and to the benzylsuccinyl radical (*k*_5_/*k*_–5_ and *k*_7_/*k*_–7_, respectively) resulted in an increase in the predicted KIE.

**Figure 4:**
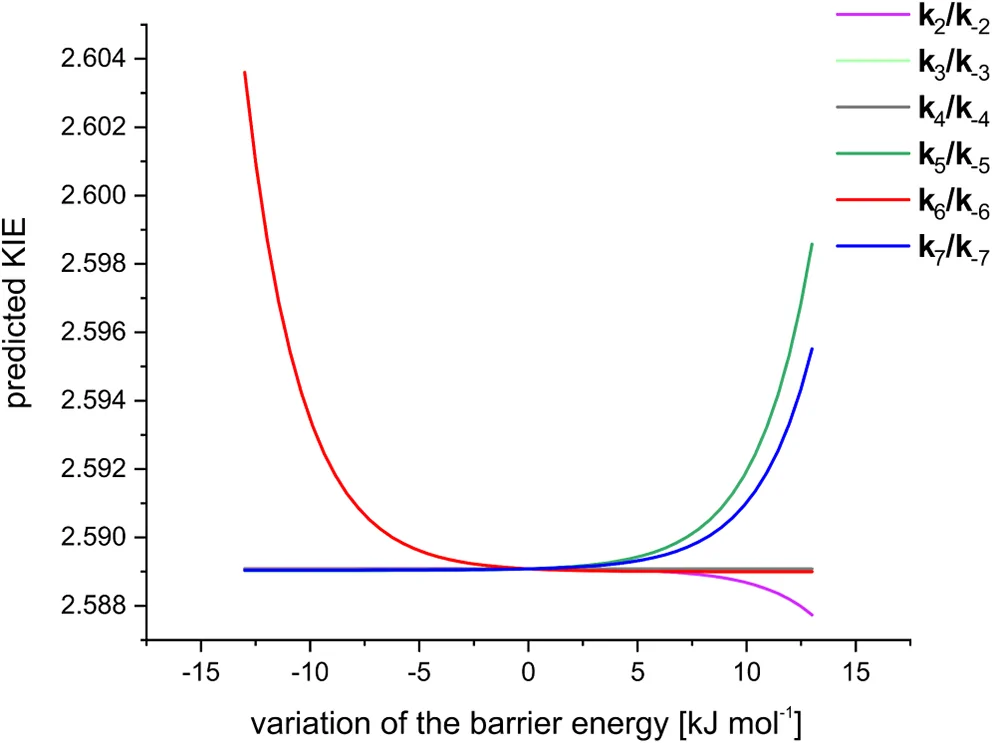
Error estimation for the predicted KIE based on a microkinetic model assuming a ±12.96 kJ/mol error range for each energy barrier.

The predicted value of KIE is in good agreement with the experimentally determined value of 2.13 ± 0.1 for the kinetic isotope effect on *V*_max_ determined in a direct assay (Figure 5A).

**Figure 5:**
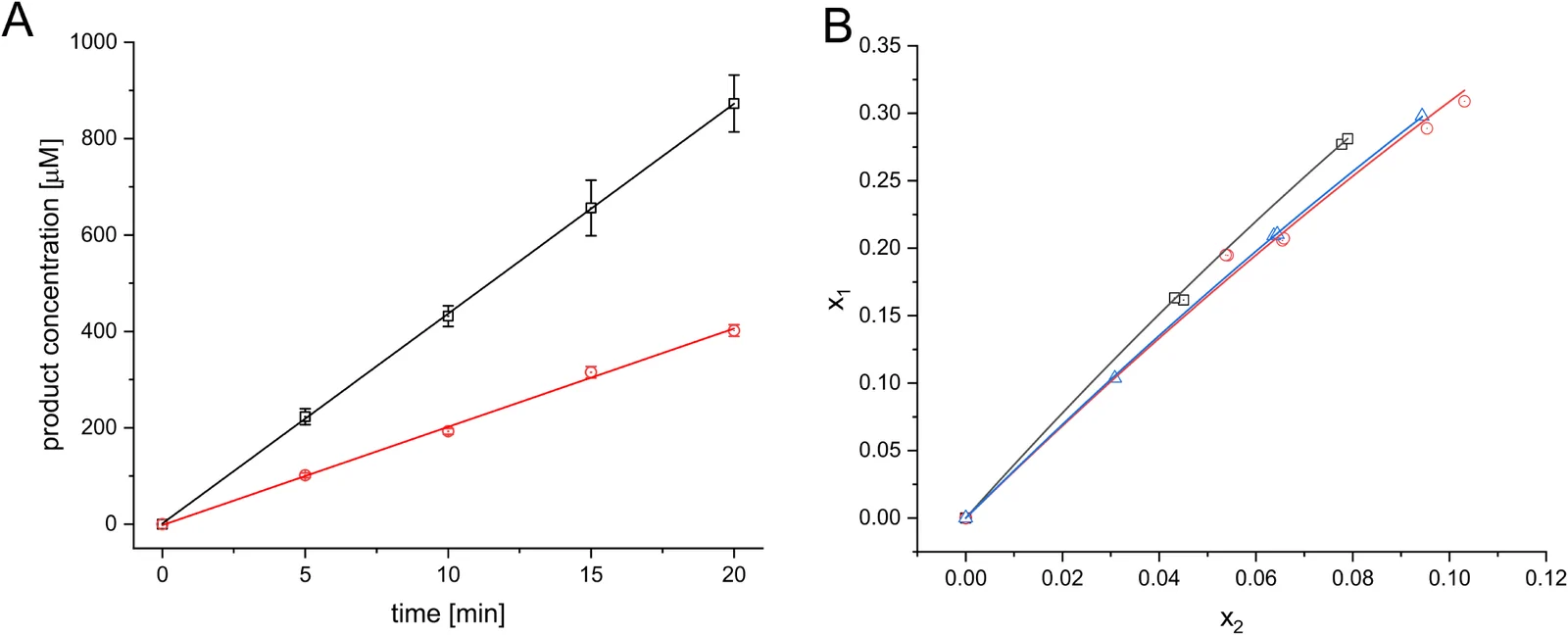
Kinetic isotope effect experiments: A) direct measurement with a kinetic curve for toluene (black, squares) and d_8_-toluene (red, circles); error bars depict the standard deviation from three experiments, B) competitive measurement—three independent replicates.

Values of KIE on (VK)D obtained in the competitive experiment (Figure 5B) from three independent reactors were 4.01, 3.5, and 3.56, yielding an average value of 3.69 ± 0.16.

As the (VK)D value turned out to be higher than that of the direct KIE, we investigated which conditions may lead to greater suppression of the intrinsic kinetic isotope effect than the (VK)D value. To achieve this, we derived the equation for ^*D*^(*V*/*K*) assuming that *K_m_* is approximated by eq 8, due to the relatively high concentration of the second substrate, fumarate (see eqs S11–S12).

11 $$K_{m}=\frac{k_{-1}+k_{cat}}{k_{1}}$$

12 $$\frac{k_{cat}}{K_{m}}=\frac{1}{k_{‐1}+k_{cat}}\frac{k_{1}k_{2}k_{3}k_{4}k_{5}k_{6}k_{7}}{\begin{array}{l} k_{2}k_{3}k_{4}k_{5}k_{6}+k_{2}k_{3}k_{4}k_{5}k_{7}+k_{2}k_{3}k_{4}k_{5}k_{‐6}+k_{2}k_{3}k_{4}k_{6}k_{7}+k_{2}k_{3}k_{4}k_{7}k_{‐5}+k_{2}k_{3}k_{4}k_{‐5}k_{‐6}+k_{2}k_{3}k_{5}k_{6}k_{7}\\ +k_{2}k_{3}k_{6}k_{7}k_{‐4}+k_{2}k_{3}k_{7}k_{‐4}k_{‐5}+k_{2}k_{3}k_{‐4}k_{‐5}k_{‐6}+k_{2}k_{4}k_{5}k_{6}k_{7}+k_{2}k_{5}k_{6}k_{7}k_{‐3}+k_{2}k_{6}k_{7}k_{‐3}k_{‐4}\\ +k_{2}k_{7}k_{‐3}k_{‐4}k_{‐5}+k_{2}k_{‐3}k_{‐4}k_{‐5}k_{‐6}+k_{3}k_{4}k_{5}k_{6}k_{7}+k_{4}k_{5}k_{6}k_{7}k_{‐2}+k_{5}k_{6}k_{7}k_{‐2}k_{‐3}+k_{6}k_{7}k_{‐2}k_{‐3}k_{‐4}\\ +k_{7}k_{‐2}k_{‐3}k_{‐4}k_{‐5}+k_{‐2}k_{‐3}k_{‐4}k_{‐5}k_{‐6} \end{array}}$$

Since we had already obtained predicted values of *k_cat_* from eq 9 (7.94 × 10^–3^ s^–1^ and 3.07 × 10^–3^ s^–1^ for toluene and d_8_-toluene, respectively), we only needed to estimate the potential values of *k*_1_ and *k*_–1_ for both substrates so that the final value of *K_m_* matched the apparent experimental value (*K_m_* = 0.216 ± 0.07 mM—see Figure S3). This value is matched at a *K_d_* of 0.214 (*k*_–1_/*k*_1_), indicating strong binding of the substrate to the enzyme. Unfortunately, we do not have data on the *K_m_* for d^8^-toluene. When we assume the same value for ^*D*^ *k*_–1_ and ^*D*^ *k*_1_ as for the unlabeled substrate, the value of ^*D*^(*V*/*K*) cannot be higher than that of the KIE, regardless of the values of the fitted constants or the values of the internal kinetic constant. Therefore, we decided to determine the conditions under which the ^*D*^(*V*/*K*) value equals the experimentally determined value (3.69), using the predicted KIE (2.58) and the known experimental *K_m_* for unlabeled toluene. Since the resulting system of equations is underdetermined, we assumed equal values of *k^H^*_–1_ and *k^D^*_–1_ as 1 s^–1^. For such initial values, the predicted *k^H^*_1_ equals 4.6664 s^–1^ M^–1^ and *k^D^*_1_ equals 3.2583 s^–1^ M^–1^, yielding ^*D*^ *K*_*d*_ and ^*D*^ *K*_*m*_ values of 0.307 and 0.308 mM, respectively. The value of ^*D*^(*V*/*K*) can only become higher than that of the KIE if the value of ^*D*^ *K*_*m*_ is higher than that of *K_m_* (i.e., 1.42-fold higher). Based on those results, one can calculate the difference in the ΔG of binding between toluene and d^8^-toluene using the relation with the equilibrium constant. The calculated difference between Δ*G* for toluene (−21.27 kJ/mol) and d^8^-toluene (−20.38 kJ/mol) shows preferential binding of toluene by only 0.89 kJ/mol, which seems a probable value associated with the substitution of all protium atoms by deuterons.

### Effect of Tunneling on the Prediction of KIE

Hydrogen atom transfers are usually quite sensitive to quantum tunneling effects, resulting in facilitated reactions proceeding “under the barrier”. We investigated this issue qualitatively using Wigner’s corrections.^48^ Table 1 summarizes all kinetic rate constants (*k_i_*) with the corrections for tunneling (Γ­(*T*)*k_i_), as well as values of intrinsic KIE (iKIE), and tunneling-corrected iKIE^t^. The data indicate a significant acceleration of toluene activation (*k*_3_, KIE increased from 7.4 to 9.6) and the opposite effect on the quenching of the radical product (*k*_–7_ KIE increased from 7.5 to 10). When using our kinetic model assuming radical quenching as an essentially irreversible step, the predicted rates for the toluene and d_8_-toluene conversions were 9.33 × 10^–3^ s^–1^ and 3.6 × 10^–3^ s^–1^, respectively, which do not change the KIE value predicted without tunneling (2.59). Similarly, assuming that only a slight correction of the estimated values of ^*H*^ *k*_1_ and ^*D*^ *k*_1_ (4.6728 s^–1^ M^–1^ and 3.2638 s^–1^ M^–1^) is needed to obtain *K_m_* for toluene at the experimental value of 0.216 mM, the predicted ^*D*^(*V*/*K*) is 3.69. These results demonstrate that tunneling effects increase the overall reaction rates by approximately 17% (precisely 17.5% and 17.2%, respectively, for toluene and d^8^-toluene) but do not influence the observed values of KIE and ^*D*^(*V*/*K*) at 30 °C.

### Effects of the Transmission Coefficient on the Prediction of KIE

The semiclassical transition state theory used in this study to predict the rate of the reaction assumes that the transmission coefficient κ for each microkinetic step equals 1. This implies that upon reaching the transition state, the atom always travels to the product basin and does not revert to its initial state. Although this was not the main objective of our study, we decided to evaluate the potential influence of the recrossing process on the predicted value of KIE. We assumed a range of κ values based on the curvature of the transition state (derived from the imaginary frequency associated with the particular transition state) in the range of 0.4 to 0.95, assuming slightly higher values (by 0.05) for the transfer of deuterium relative to the protium atom (see Figure S4 and Table S9 for details). The final rate constant was the product of the transmission factor, Wigner’s correction for tunneling, and the exponential term that depends on energy corrected by thermal energy (κ*Γ­(*T*)*k_i_). As expected, our equation predicts a slight lowering of the overall reaction rates to 6.5 × 10^–3^ s^–1^ and 2.7 × 10^–3^ s for toluene and d_8_-toluene, respectively, which results in a slight reduction of the predicted KIE from 2.58 to 2.41. To evaluate the error of the estimate, we conducted a sensitivity analysis of KIE to assumed values of κ by varying the transmission coefficients by amounts in the range of −0.1 to 0.1. The variation was introduced either uniformly to all microkinetic constants or individually to each step (i.e., influencing forward and reverse steps by the same degree). The analysis showed that the value of KIE is relatively insensitive to the value of κ, as it varies over the narrow range of 2.38–2.43 (Figure S5). The analysis conducted for individual steps revealed that for most barriers the dependence is minimal (below 10^–5^) with one significant exception of the C–C bond formation step (*k*_6_/*k*_–6_), which is sensitive to those changes (see Figure S6).

### Estimation of the Rate of Product Release

Using the same approach as described above, we investigated whether the model also allows the prediction of the kinetic constant for product release, and how its value influences the predicted KIE. To achieve this, we derived a kinetic equation assuming that the last irreversible step is the release of the product from the enzyme, with Cys493 still in the thiyl radical state (see the SI for the derivation, eq S13, and Scheme S2). Accordingly, the equation involves a step, *k*_8_, for which we do not have any theoretical or experimental data; we can only use it to fit the value of *k*_8_ to reproduce the experimentally observed value of KIE. As an approximation, we assumed that *k*_8_ and ^*D*^ *k*_8_ were equal and fitted both to 1.978 × 10^–2^ s^–1^. With this assumption, the model reproduced exactly the experimental KIE of 2.13. When Wigner tunneling is taken into account, a value of 2.341 × 10^–2^ s^–1^ for *k*_8_ and ^D^k_8_ would be consistent with the KIE value measured in the experiment.

### H/D Exchange

Recently, we have discovered that BSS catalyzes a slow isotope exchange of one D atom for an H during the reaction between d_8_-toluene and fumarate in H_2_O, as well as an exchange of one or two H atom(s) of benzylsuccinate for D when incubated in D_2_O (Figure S7).^5^ Within the BSS active site, the only residue capable of supporting such an exchange is Cys493 (with H or D transferred from either toluene or benzylsuccinate; Figure 1B). The S–H bond of Cys is polarized enough to allow isotope exchange with the solvent (most likely involving Gln707, which forms a hydrogen bond with Cys493). Due to the instability of BSS and its very high propensity for deactivation by oxygen deactivation, it has not been possible to directly measure the H/D exchange rate at Cys493, e.g., via NMR-based methods.^53^ Despite this, we used our calculations to estimate the most probable mechanism underlying the observed H/D exchange reaction that leads to the formation of both d_1_- and d_2_-substituted benzylsuccinate during incubation in 40% D_2_O. To approach this problem, we have analyzed the potential pathways leading to the formation of d_1_- and d_2_-substituted benzylsuccinate (Figure 6).

**Figure 6:**
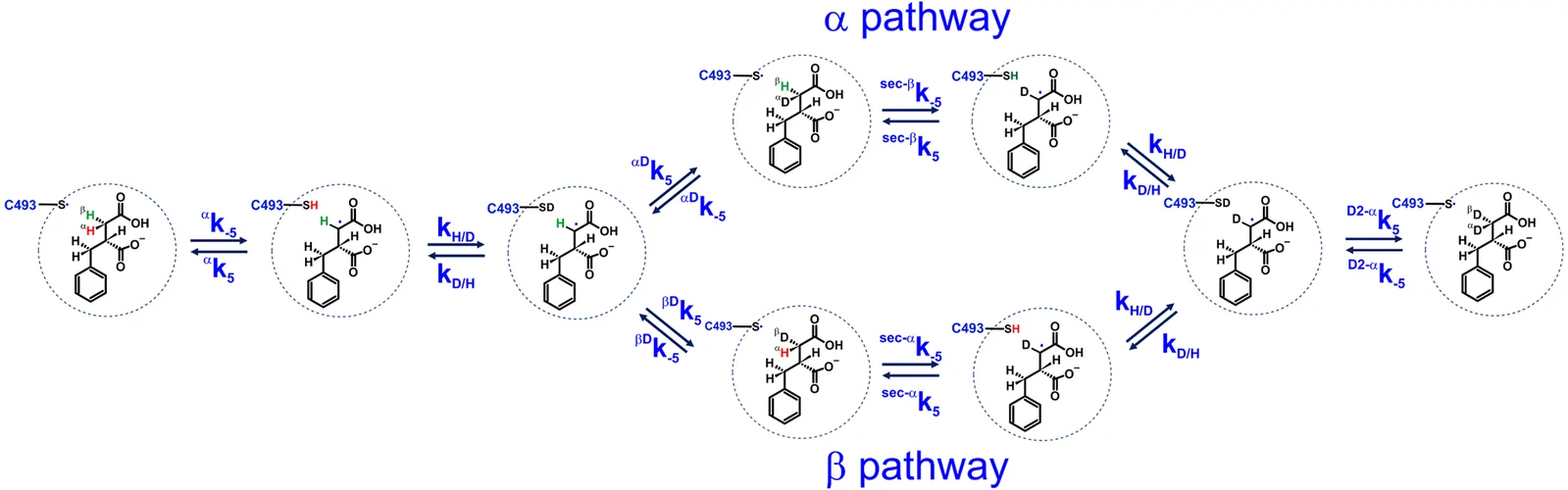
Two potential pathways leading to the formation of the d_2_-benzylsuccinate product.

First, we examined the pathway for the formation of d_1_-benzylsuccinate from benzylsuccinate. Such a process requires the removal of an H atom from C2 of the product at the kinetically preferred α position, at rate ^α^ *k*_–5_, followed by *H*/*D* exchange at the Cys-SH group and final back-transfer of the D atom to the benzylsuccinyl radical at rate ^α*D*^ *k*_5_ for the α pathway or ^β*D*^ *k*_5_ for the β pathway (Figure 6, Table S10). Here, we do not consider the removal of the initial H atom from the kinetically unfavorable β position, as the ratio of rate constants is almost 10 orders of magnitude in favor of ^α^ *k*_–5._ To derive the kinetic equation describing the rate of formation of d_1_-benzylsuccinate we made the following assumptions: (i) formation of d_1_-benzylsuccinate is irreversible due to a surplus of unlabeled substrate and very low concentrations of the deuterated product, (ii) the enzyme is in equilibrium with benzylsuccinate, so the binding or release of the product does not affect the observed exchange rate, (iii) *k*_*H/D*_ is a product of *k* ^*HDX*^ and the concentration of *D* ^+^ in the enzyme, while *k*_*D/H*_ is a product of *k* ^*HDX*^ and the concentration of *H* ^+^ in the enzyme. The latter assumption stems from the fact that the enzyme is in equilibrium with the solvent, and because we do not know the molecular mechanism of *H*/*D* or *D*/*H* exchange at Cys493, we assume that the availability of protons/deuterons in the active site is the only relevant parameter. With these assumptions, we constructed Schemes S3 and S4 of the reaction according to the King–Altman procedure (derivation, eqs S14–S25) and therefrom derived:

13 Vαd1=k7αDk−7αkH/D[ET]k−7αkH/D+kH/Dk7αD+k7αDk7α+kD/Hk7α+k7αDk−7α+kD/Hk−7α

14 Vβd1=k7βDk−7αkH/D[ET]k−7αkH/D+kH/Dk7βD+k7βDk7α+kD/Hk7α+k7βDk−7α+kD/Hk−7α

where *V* ^α*d*1^ or *V* ^β*d*1^ are the reaction velocities of α-*d*_1_-benzylsuccinate ((2R,3R)-2-benzyl­(3-^2^H_1_)­butanedioic acid) or β-d_1_-benzylsuccinate ((2R,3S)-2-benzyl­(3-^2^H_1_)­butanedioic acid) formation, respectively, and [*E*_*T*_] is the total concentration of the enzyme. The predicted reaction velocities clearly indicate that the preferential mechanism proceeds along the kinetically preferred α pathway, with *V* ^α*d*1^ being 6 × 10^8^ times higher than *V* ^β*d*1^. Therefore, we assume that the formation of the initial benzylsuccinyl radical through the β pathway is negligible.

In addition to the detected d_1_-benzylsuccinate we also detected a d_2_-labeled product formed by H/D exchange in D_2_O. Therefore, we examined the most probable pathway for the formation of d_2_-benzylsuccinate. Such a product can potentially be obtained via two alternative H/D exchange mechanisms, as depicted in Figure 6. After the formation of the benzylsuccinyl radical and H/D exchange at Cys493, the first deuteron can be transferred either to the α position (i.e., TS4^α^, kinetic constant ^α*D*^ *k*_5_) or to the β position (i.e., TS4^β^, kinetic constant ^β*D*^ *k*_5_) of the benzylsuccinyl radical. In the first case, the reaction continues via removal of the β H atom (kinetic constant ^sec‑β^ *k*_–5_), which is influenced by a secondary KIE introduced by the D atom at the same C atom, while in the other case, the α H atom will be removed at a rate (kinetic constant ^sec‑α^ *k*_–5_) that is also modulated by secondary KIE. Both pathways lead to the formation of a d_1_-benzylsuccinyl radical as an intermediate. The d_2_-product can then be obtained after another H/D exchange at Cys493 and transfer of the D atom to the d_1_-benzylsuccinyl radical via the kinetically preferred α pathway at a rate (^*D*2α^ *k*_5_) that is influenced by primary and secondary KIE. Here again, we discard the theoretically possible pathway for the second deuteron transfer along the β pathway, as ^*D*2β^ *k*_5_ is 7 × 10^8^ times lower than ^*D*2α^ *k*_5_, i.e., such a transfer would not contribute significantly to the overall product. The calculated kinetic constants for the two remaining plausible pathways (α and β) are collected in Table S10. In Schemes S5 and S6, we assumed that the last step of the process is irreversible due to the extremely low initial concentration of the products compared with the concentration of unlabeled benzylsuccinate. We derived the overall equations for reaction velocities of d_2_-benzylsuccinate formation (*V* ^*d*2^) for both pathways using the King-Altman method^49^ (see eqs S26–S41 as well as Schemes S5–S6).

In order to compare the values of both *V* ^*d*1^/[*E_T_*] and *V* ^*d*2^/[*E_T_*], we need to assume the value of *k^HDX^* from which the *k_H/D_* values could be derived. As in the case of *k*_8_, we have no way to measure this rate, and the mechanism of the H/D exchange in BSS has not been investigated. To estimate the range of potential values of the kinetic constant for H/D exchange, we first approximated the rate of d_1_-benzylsuccinate formation observed in the experiment. The average rate of formation of d_1_-benzylsuccinate was 1.34 × 10^–10^ M s^–1^ (1.94 μM in 4 h).^5^ The process was catalyzed by 9.8 mg of cell extract (total protein), which consists of BSS to approximately 10%, yielding a BSS concentration of 4.3 × 10^–9^ M^54^ (molecular mass of the α_2_β_2_γ _2_ holoenzyme of 226 kDa). Assuming that only one active site per BSS heterohexamer is active, an estimated rate of d_1_-production of 0.06 s^–1^ is obtained. Assuming a 4:6 ratio of *k_H/D_*:*k_D/H_* based on the 40% D_2_O concentration used in the experiment, we estimated *k_H/D_* at 111 s^–1^ and *k_D/H_* at 167 s^–1^. These values were obtained assuming a value for *k^HDX^* of 5 s^–1^ M^–1^, and 40% D_2_O to be 22 M of D_2_O and 33 M of H_2_O. With these assumptions, we obtained a value for *V* ^α*d*1^/[*E_T_*] of 0.06 s^–1^, or *V* ^β*d*1^/[*E_T_*] of 1 × 10^–10^ s^–1^ while that of *V* ^*d*2^/[*E_T_*] was 5.9 × 10^–11^ s^–1^ or 1.37 × 10^–10^ s^–1^ for pathway α or β, respectively. These data indicate that the double exchange via the β pathway is 2.3 times faster than via the α pathway, which indicates that i) the β pathway is the preferential mechanism for the formation of d_2_-benzylsuccinate and ii) the d_1_- and d_2_-products are being formed in a different manner. Furthermore, the analysis of relative species concentrations along the d_2_-pathways indicates the highest concentration of α-D-benzylsuccinate for the α pathway (65%), suggesting that at this point the monosubstituted product has a chance of dissociating from the enzyme. This is due to the fact that the next step, the removal of the H atom from the β position, is the rate-limiting step of the whole pathway. In contrast, along the β pathway, the highest concentrations are predicted for [Cys-S^.^:BS] and [Cys-SH:BS^.^] (approx. 50%), and the concentration of d_1_-product is negligible. It should be underlined, however, that experimental concentrations of d_2_-product were far higher than predicted by the microkinetic analysis (only 4.4-fold lower than for the d_1_-product). This indicates that the barrier for exchange of the β H atom may be significantly overestimated, and in reality, the process has to proceed faster to yield products in experimentally determined ratios.

### Effect of Tunneling on the HDX Process

When we included Wigner’s tunneling correction in the H/DX analysis, the experimentally observed rate of d_1_-product formation could not be obtained for equal values of *k_H/D_* and *k_D/H_*, and the limit of the predicted rate when *k^HDX^* approaches infinity was 0.0284 s^–1^. To reproduce the experimental value of *V* ^*d*1^, we had to assume *k^HDX^* of 61.5 s^–1^ while leaving *k^DHX^* at 5 s^–1^. This change resulted in an increase in *k_H/D_* to 1366 s^–1^ and in *k*_*D/H*_ only to 166.7 s^–1.^ As a consequence of tunneling acceleration and an increase in *k_H/D_* the rate of *V* ^β*d*1^ increased by 6-fold, *V* ^α^ *d* ^2^ by by 3.6-fold, and *V* ^β*d*2^ by 23-fold. However, the relative order of the pathways’ velocities remained the same as in modeling without the tunneling effect.

## Discussion

### Kinetic Isotope Effect

In this paper, we have demonstrated a rigorous way to conduct joint theoretical and experimental analysis of a complex reaction system, namely benzylsuccinate synthase. We developed a microkinetic equation that includes as many steps as possible to reliably describe the reaction using a theoretical approach. Then, we verified the equation’s robustness by benchmarking its predictive power against experimental data and performing sensitivity analysis. Finally, we used the same kinetic-equation framework to explain discrepancies and estimate values of kinetic constants that we could not measure or estimate using the selected modeling method. This approach can be applied to any chemical reaction for which a molecular-level mechanistic hypothesis is known.

The experimental results obtained from two independent experiments in this study are consistent with previous observations by Li and Marsh on BSS from *Thauera aromatica* T1^2^. The observed (VK)D from the competitive method (3.7 ± 0.3) was higher than that recorded for *V*_max_ determinations (2.1 ± 0.1), similarly as reported by Li and Marsh (values of 2.9 ± 0.1 and 1.7 ± 0.2, respectively). The KIE observed in the direct experiment was twofold lower than in our previous experiments,^39^ which may be explained by the improved analytical method used in this study. The value of the KIE predicted by theoretical calculation (2.58) is in reasonably good agreement with both experimental results. Our analysis shows that KIE can attain lower values than (VK)D only when binding of toluene proceeds faster than that of d_8_-toluene. We use this fact and experimental values to estimate binding kinetic constants. Furthermore, we have shown that product release can, to some extent, mask the predicted KIE, which allows a plausible explanation of the experimental results. As an additional assurance of the robustness of our microkinetic model, we conducted a sensitivity analysis for the prediction of theoretical KIE, demonstrating its resistance to inevitable errors introduced by the modeling. The sensitivity analysis presented in Figure 4 demonstrates that the predicted KIE value is not strongly affected by errors in barrier height estimation at least within a range of ±12.96 kJ mol^–1^. An overestimation of the barrier associated with HAT between Cys and Gly (a step not involved in shifting any isotopically labeled H atom) could mask the overall KIE when it becomes rate-limiting. Lowering the barrier associated with C–C bond formation resulted in an increase in the KIE value (due to unmasking of the effect), while a significant increase in barriers associated with HAT of the isotopically labeled H atom (activation of toluene and quenching the benzylsuccinyl radical) had only a minor influence on the increase of the KIE by 0.01. The overestimation of the KIE value with respect to the experiment may also be due to the assumed unity of the transmission coefficients used in our approach. Our estimative analysis showed, however, that including κ (together with a correction for tunneling) in the prediction would only yield a small decrease in the KIE, up to 0.2. The overall ratio of reaction rates is only noticeably affected by the reaction step associated with C–C bond formation. Overall, the good agreement between calculated and experimental values indicates that the model has reasonable predictive capability for the proposed reaction pathway and its barriers. We also estimated that the approximate tunneling effect may accelerate the observed rate by 17% relative to that predicted by classical TST theory, but does not materially change the estimated KIE values.

The kinetic model described by eq 9 is similar to that used by Li and Marsh in a previous study,^2^ but takes into account all internal chemical steps of benzylsuccinate formation, instead of assuming C–C bond formation as an irreversible step, as predicted by gas-phase modeling. Furthermore, our analysis is based on the kinetic constants derived from QM/MM modeling in the presence of the BSS catalytic subunit, while the previous analysis used the gas-phase energy profile^1^ that is based only on predicting the reaction in the absence of the enzyme (treated there as a model of the uncatalyzed reaction). The analysis of both kinetic models (eqs 9 and 11) derived in this study shows that the experimentally observed suppression of the KIE with respect to ^*D*^(*V*/*K*) can be caused by a higher value of *K_m_* for deuterated toluene and by masking of the KIE due to product release. The difference in binding constant of deuterated and nondeuterated substrates to the BSS active site may stem from different vibrational modes of the deuterated compound and altered interactions with both the solvent and the binding cavity of the enzyme. These two factors enable the complete reproduction of the experimentally observed trends in isotope effects.

Using kinetic models, we were also able to estimate the magnitude of the product release rate. Although this analysis is inevitably based on several guesses (such as exact values of rates describing substrate binding and release), we propose that the product release rate would be on the order of 0.02 s^–1^. Such a rate would be comparable to the overall rate of the reaction (0.008–0.009 s^–1^) and, as a result, would contribute to some extent to the observed suppression of the KIE.

### HDX

Finally, we propose a mechanism through which the observed low-level HDX effects occur during the reaction with d_8_-toluene or during the incubation of benzylsuccinate in D_2_O. Making several assumptions, we used the measured rates of d_1_-benzylsuccinate formation and the rate equation based on QM/MM calculations to estimate the potential rate of H/D exchange in the enzyme. The estimated microkinetic rates of the H/D and D/H exchanges were in the range of 5 s^–1^ M^–1^, which, assuming the potential concentration of protons and deuterons in the enzyme is the same as in the bulk solution, translates to an exchange rate in the range of 100–160 s^–1^. This would make the exchange process significantly slower than the rate of C–C bond formation but comparable to that of radical quenching (300–900 s^–1^ – 3–9 times slower). These estimates qualitatively agree with the experimental results observed, i.e., a 16-fold slower production of d_7_-benzylsuccinate compared to d_8_-benzylsuccinate.^5^ We also show that the β pathway would be preferred for the formation of d_2_-substituted benzylsuccinate, although the experimentally observed concentration of the product exceeded the significantly slower predicted rate for either pathway. Including the tunneling effect differentiated the estimated rates of H/D exchange at Cys493 and D/H exchange; consequently, the H/D exchange was 8.2 times faster, and together with the acceleration of HAT processes by tunneling, we predicted the most significant acceleration of the β pathway leading to d_2_-benzylsuccinate (by 23-fold). Therefore, we speculate that, to explain the experimentally detected quantities of d_2_-product, the slowest step of the β pathway, i.e., removal of the β-H atom and its exchange for deuterium, should proceed faster, indicating an overestimation of the associated barrier in our QM/MM modeling.

## Summary

In this study, we describe microkinetic modeling of the benzylsuccinate synthase reaction based on QM/MM-derived reaction profiles that yield predictions of kinetic isotope effects. The analysis of the microkinetic equations allows correlation with kinetic experiments and enables estimation of experimentally inaccessible processes (or those difficult to measure for a radical enzyme), such as H/D exchange rates and rates of substrate binding and product release. We have shown that the predicted KIEs may not be very sensitive to inevitable errors in the prediction of transition-state barriers when the mechanism comprises many steps with barriers of similar heights. We have also shown that transition-state theory still provides a reasonable approximation for predicting KIE values, as the transmission coefficient has its greatest influence on the last reversible step of the reaction.

We also show that microkinetic analysis can be used to explore which mechanistic hypotheses make the most sense in light of experimental results. This approach provides a framework for planning experiments that can more reliably test mechanistic hypotheses as well as pinpoint critical parts of the mechanisms for which modeling produced implausible estimates, such as an excessively high barrier for β-H atom removal in the BSS H/D exchange mechanism.

## Supplementary Material





## Notes

Jupyter files with equation derivation, fits, and sensitivity analysis (ZIP)
Additional information on methods: LC-MS/MS methods; NGS sequencing; QM/MM modeling; derivation of kinetic equations; BSS sequence; Michaelis–Menten model fit to kinetic data; results on QM/MM modeling, results on the recrossing approximation; tables of kinetic constants; experimental results on HDX (PDF)
