# Supplementary material for: The Origin of the Kinetic Isotope Effect and Mechanism of H/D Exchange in Radical C–C Bond Formation Catalyzed by Benzylsuccinate Synthase: Experiments and Microkinetic Modeling
Source: J Phys Chem B. 2026 Aug 28;130(36):9045–57. doi: 10.1021/acs.jpcb.6c01533 (PMC13573558; doi:10.1021/acs.jpcb.6c01533)
Supplement: Supplementary file 1 [file jp6c01533_si_002.pdf]

Supporting information

The origin of the kinetic isotope effect and mechanism of H/D exchange in radical C-C bond formation catalysed by benzylsuccinate synthase: experiment and microkinetic modelling

Maciej Szaleniec[1*], Gabriela Oleksy[1,2], Ivana Aleksic[1], Kai Krämer[2], Tomasz Borowski[1], Johann Heider[2,3*]

1 - Jerzy Haber Institute of Catalysis and Surface Chemistry, Polish Academy of Sciences, Kraków 31-201, Poland; email: maciej.szaleniec@ikifp.edu.pl
2 - Department of Biology, Laboratory for Microbial Biochemistry, Philipps University Marburg, 35043 Marburg, Germany; email: heider@staff.uni-marburg.de
3 - SYNMIKRO Research Center, 35043 Marburg, Germany

Index

METHODS 2
LC-MS/MS methods........ 2
Calibration curves for LC-MS/MS and LC-DAD benzylsuccinate quantitation 3
Short-read NGS sequencing 3
Long-read nanopore sequencing........ 3
Genome assembly and annotation 3
QMMM model -- QM1 and QM2 4
Derivation of the rate equations 5
Whole reaction 5
The whole reaction extended model 9
HDX leading to d_1-benzylsuccinate – alpha pathway 11
HDX leading to d_1-benzylsuccinate – beta pathway 12
HDX leading to d_2-benzylsuccinate – alpha pathway 14
HDX leading to d_2-benzylsuccinate – beta pathway 16
RESULTS 19
BSS sequence........ 19
QM:MM energies 21
Kinetic isotope effect........ 24
Approximation of transmission coefficients 28
H/D Exchange 29

METHODS

LC-MS/MS methods

Table S1 Parameters of jet-stream ESI ion source used in the LC-MS/MS analysis.

Parameter	Value (+)	Value (-)
**Gas Temp [°C]**	300	300
**Gas Flow [L/min]**	10	10
**Nebulizer [psi]**	45	45
**Sheath Gas Heater [°C]**	300	300
**Sheath Gas Flow [L/min]**	10	10
**Capillary [V]**	3500	3500
**V Charging [V]**	500	1000

Table S2. Parameters of MRM method for the analysis of benzylsuccinate, d[7]-benzylsuccinate and d[8]-benzylsuccinate.

Compound	Precursor ion [m/z]	Product ion [m/z]	Dwell [ms]	Fragmentor [V]	Collision energy [v]	Cell accelerator [V]	Polarity
**benzylsuccinate**	207.1	163.0	200	107	10	4	negative
**d_8-benzylsuccinate**	215.0	171.0	200	107	10	4	negative
**d_7-benzylsuccinate**	214.0	170.0	200	107	10	4	negative

Table S3. Parameters for analysis of product ions of benzylsuccinate 207, 209, 209 m/z signals (respectively, $[M-H]^-$, $[M+1-H]^-$ and $[M+2-H]^-$).

Precursor ion [m/z]	MS2 from	MS2 to	Scan time [ms]	Fragmentor [V]	Collision energy [v]	Cell accelerator [V]	Polarity
207.0	150.0	212.0	500	107	10	4	negative
208.0	150.0	212.0	500	107	10	4	negative
209.0	150.0	212.0	500	107	10	4	negative

Calibration curves for LC-MS/MS and LC-DAD benzylsuccinate quantitation

A) B)

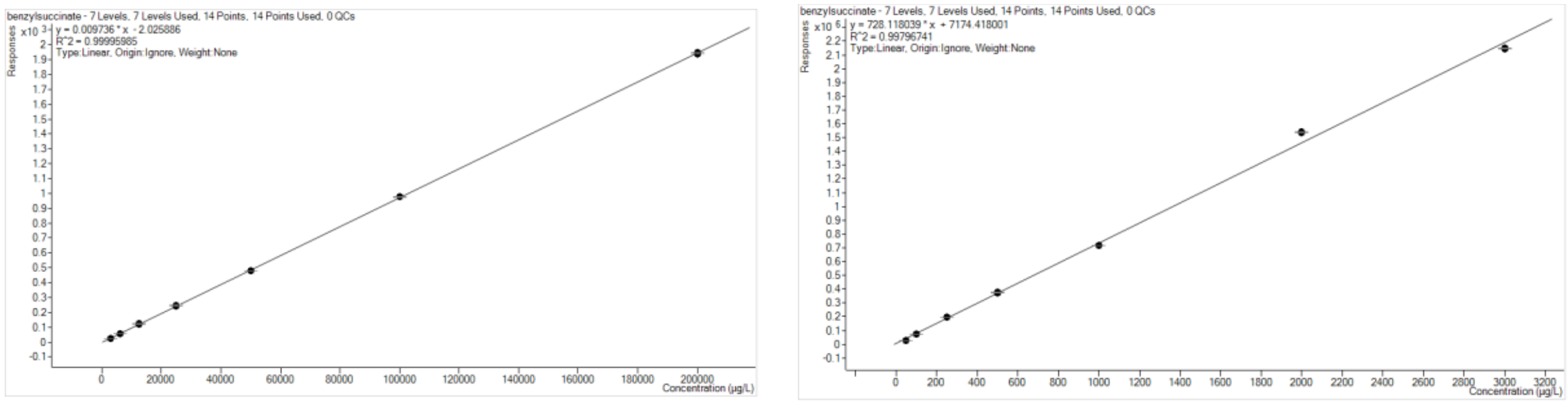

Figure S1. Calibration curves for LC-MS/MS quantitation of A) LC-DAD quantitation of benzylsuccinate; B) benzylsuccinate in SIM mode. Concentrations are given in µg/L.

Short-read NGS sequencing

Genomic DNA was extracted from bacterial cells using the Thermo Scientific™ GeneJET™ Genomic DNA Purification Kit, following the manufacturer’s instructions. Briefly, cell samples were resuspended in the digestion solution with the addition of Proteinase K and incubated at 56°C for 30 minutes. Afterwards, RNase A solution was added, followed by Lysis solution. The DNA was then purified using 50% ethanol and a purification column, where the DNA binds to the silica membrane. The final DNA was resuspended in the elution buffer, quantified using an Epoch Microplate Spectrophotometer, and assessed for quality using gel electrophoresis.

The whole genome library was prepared with the Nextera XT DNA Library Preparation Kit (Illumina) according to the manufacturer's protocol, using 1 ng of DNA as the input material. The DNA was enzymatically fragmented and tagged with adapter sequences. The tagmented fragments were subsequently amplified using the IDT for Illumina DNA/RNA UD Indexes (Illumina) and purified with the Illumina Purification beads (Illumina). The library concentration was measured with the Qubit dsDNA HS assay kit (Invitrogen) and fragment size distribution was assessed on the 2100 Bioanalyzer using the High Sensitivity DNA Kit (Agilent). Sequencing was performed on the NextSeq 2000 platform (Illumina) using the P1 300-cycles reagents kit (Illumina).

Long-read nanopore sequencing

The whole genome library for nanopore sequencing was prepared from 400 ng of DNA using the Native Barcoding Kit 24 V14 (Oxford Nanopore Technologies) following the producer's instructions. First, the DNA was end-prepped and repaired. Following the Native barcodes ligation, the library was cleaned with AMPure XP beads (Beckman Coulter) and adapter-ligated. The library was loaded on the MinION R10.4.1 flowcell (ONT) and sequencing was performed on the MinION Mk1b device (ONT) with the MinKNOW ver. 22.12.5 used for data acquisition.

Genome assembly and annotation

To obtain a high-quality genome assembly, a hybrid approach integrating both Illumina and Oxford Nanopore Technologies (ONT) reads was employed. This strategy utilized the high accuracy of short

Illumina reads for error correction and the long-read capability of ONT for enhanced contiguity. Hybrid genome assembly was conducted using Nanophase (version 0.2.3), with Flye used for assembling ONT long reads and Pilon for polishing with Illumina short reads. Genome completeness and accuracy were assessed using CheckM and QUAST. The final assembly resulted in a single bin with 99.9% completeness and only two contigs, indicating a nearly complete and highly contiguous genome. The final sequence reported by Nanophase was further annotated using the Prokaryotic Genome Annotation Pipeline (PGAP, version 2023-10-03.build7061), which predicts genes, identifies protein-coding sequences, assigns functional annotations, and detects RNA features such as tRNAs and rRNAs, ensuring a standardized and high-quality genome annotation.

QMMM model -- QM1 and QM2

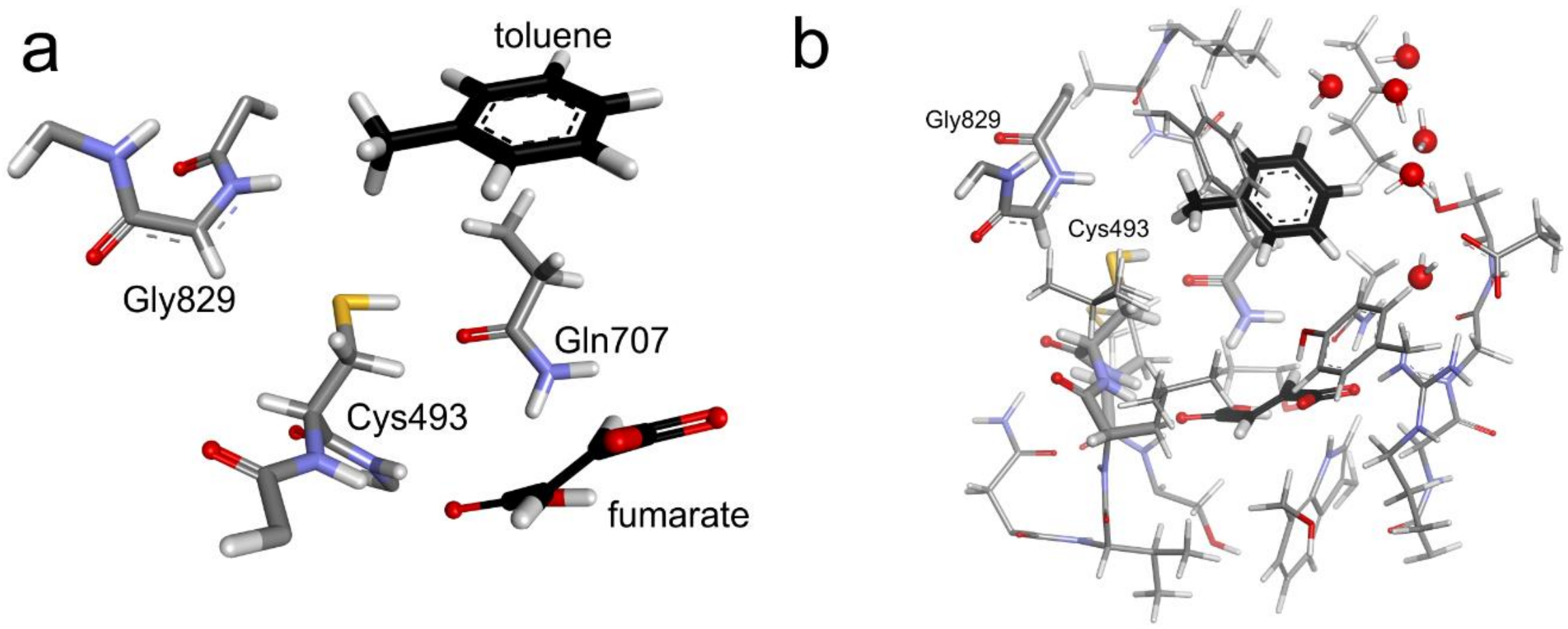

Figure S2. The QM (HL) part of the QM:MM BSS models: a) a small one (QM1) used for geometry optimization and vibrational corrections and b) a big one (QM2) used for single point energy corrections.

Derivation of the rate equations

Whole reaction

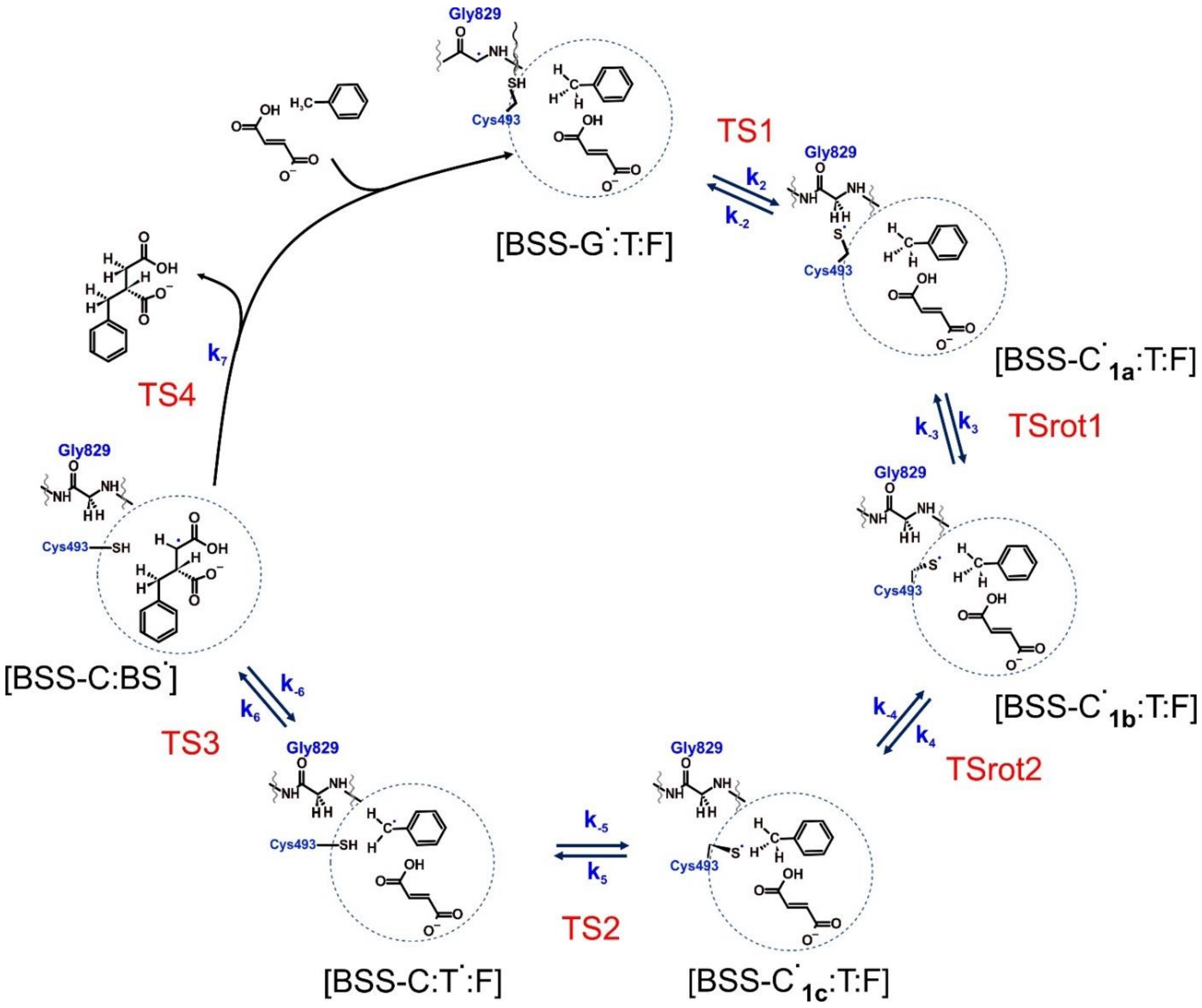

Scheme S1. The schematic representation of the whole reaction leading to the formation of benzylsuccinate assuming the irreversibility of the benzylsuccinate radical quenching step and no kinetic limitation from substrates binding/product release steps. [BSS-G·:T:F] – BSS with glycyl radical in complex with toluene and fumarate, [BSS-C·$_{1a}$:T:F], [BSS-C·$_{1b}$:T:F], [BSS-C·$_{1c}$:T:F] BSS in complex with toluene and fumarate with cysteinyl radical in conformations 1a toward Gly829, 1b toward active site , 1c toward active site corresponding to the lowest energy toluene activation, [BSS-C:T·:F] – BSS with cysteine in complex with benzyl radical and fumarate, [BSS-C:BS·] – BSS with cysteine in complex with benzylsuccinyl radical

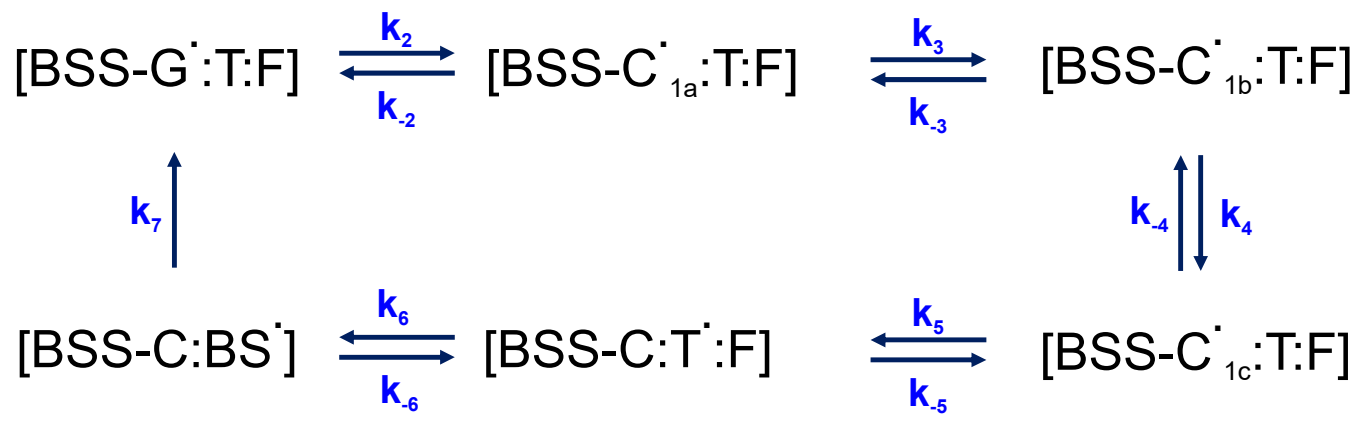

I

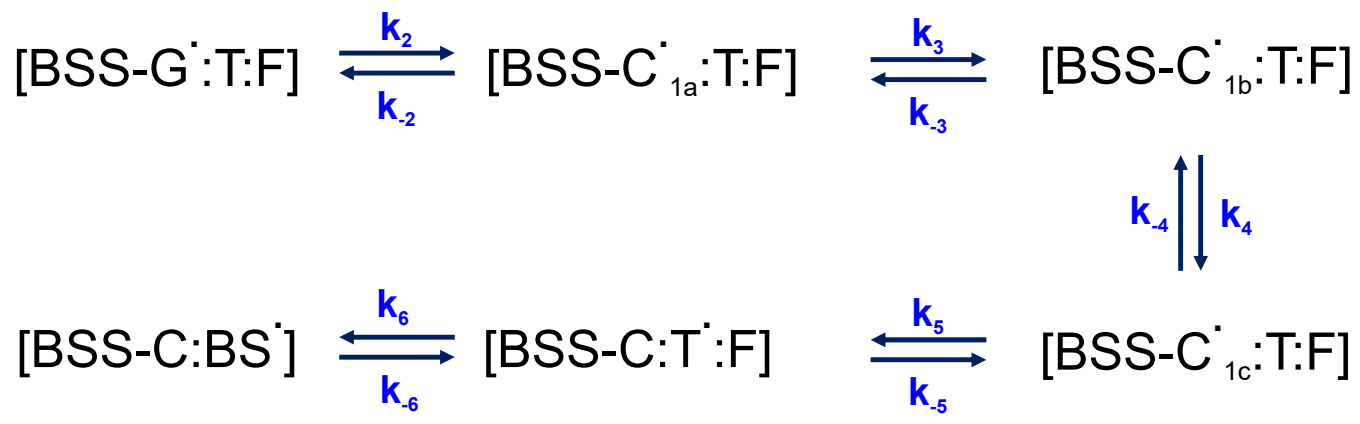

II

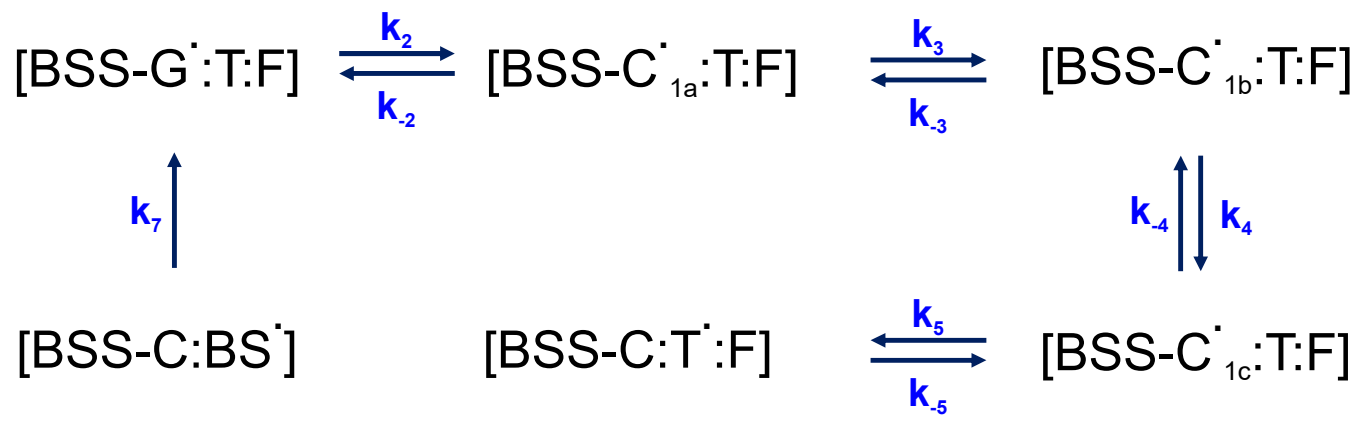

III

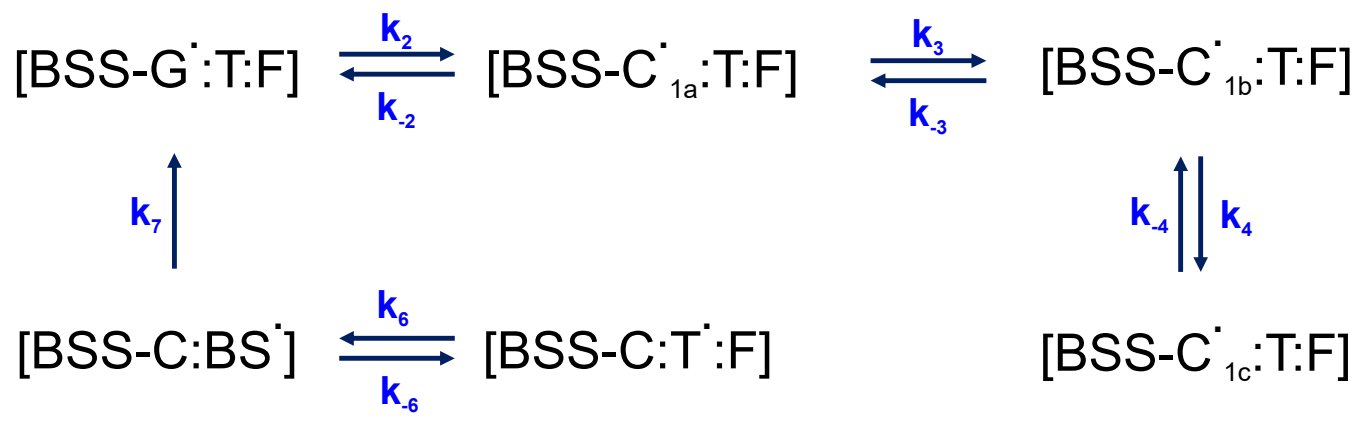

IV

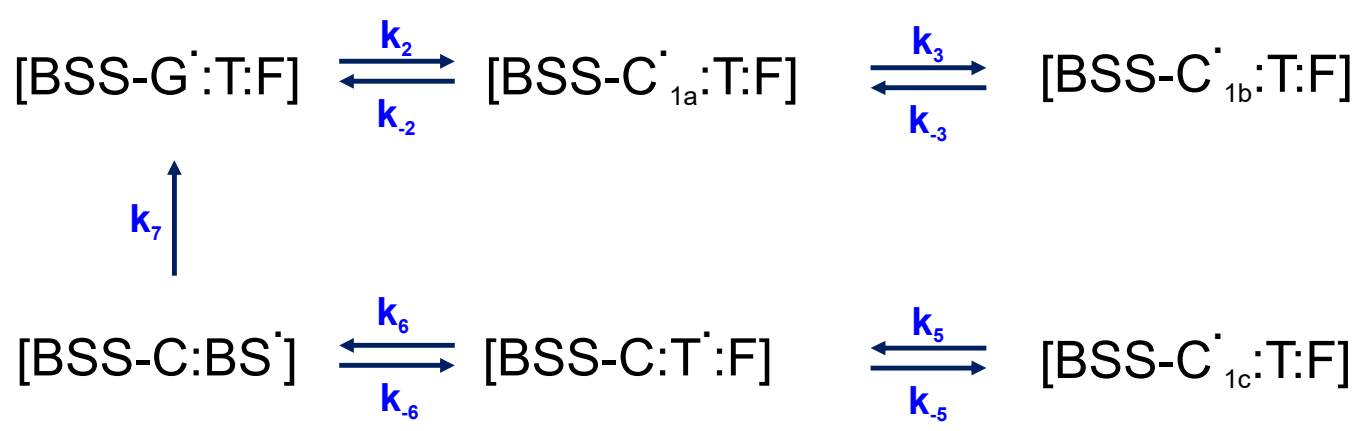

V

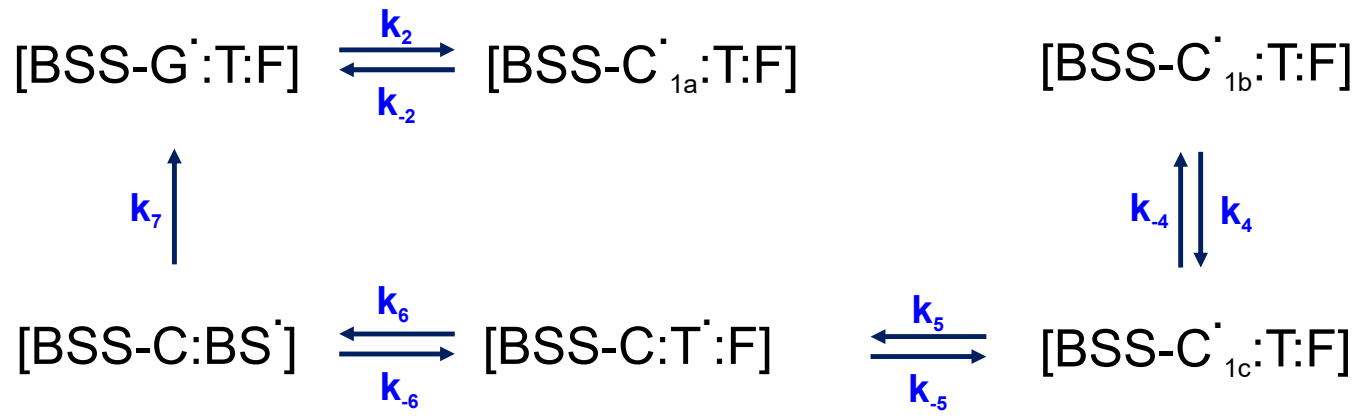

VI

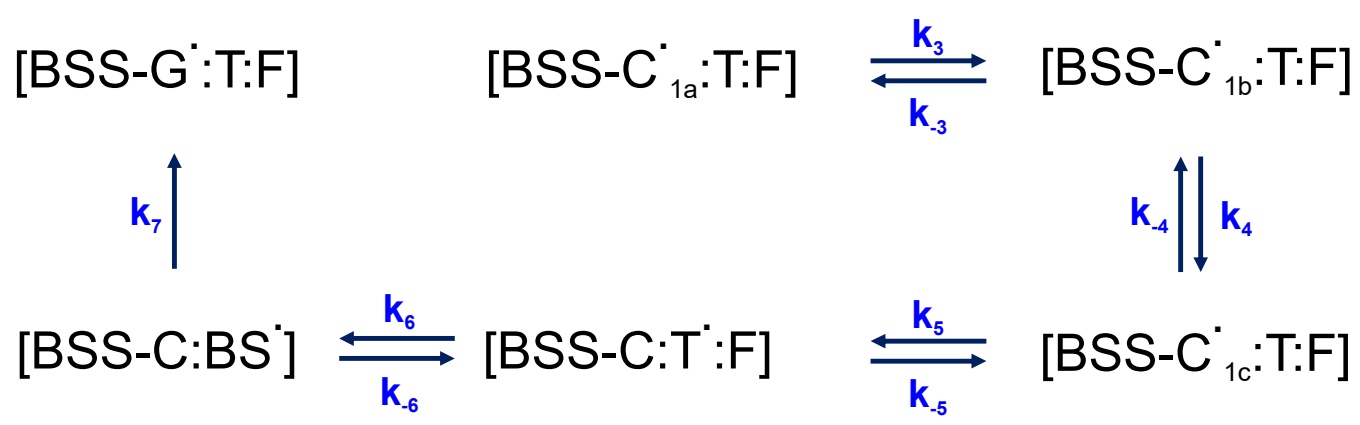

From the diagram describing internal enzyme reactions (at substrate saturation conditions) with irreversible radical quenching step (k_5) we construct four different subdiagrams (I-IV) without one transition. In each case when the pathway involves an irreversible step (k_{-5}) we multiply the product by 0 (thus removing such terms from the equation). We construct equations describing the relative concentration of each enzyme species by multiplying microkinetic constants leading to that species but not involving it. We add terms for each diagram and introduce it to the nominator of the equation.

Eq. S1 ES

$$\frac{[B-G\cdot:T:F]}{E_T} = \frac{k_{-2}k_{-3}k_{-4}k_6k_7 + k_{-2}k_{-3}k_5k_6k_7 + k_{-2}k_4k_5k_6k_7 + k_3k_4k_5k_6k_7 + k_7k_{-2}k_{-3}k_{-4}k_{-5} + k_{-2}k_{-3}k_{-4}k_{-5}k_{-6}}{\Delta}$$

Eq. S2 P1

$$\frac{[B-C_{1a}:T:F]}{E_T} = \frac{k_2k_6k_7k_{-3}k_{-4} + k_2k_{-3}k_5k_6k_7 + k_2k_4k_5k_6k_7 + k_2k_7k_{-3}k_{-4}k_{-5} + k_2k_{-3}k_{-4}k_{-5}k_{-6} + 0\cdot k_{-3}k_{-4}k_{-5}k_{-6}}{\Delta}$$
$$= \frac{k_2k_6k_7k_{-3}k_{-4} + k_2k_{-3}k_5k_6k_7 + k_2k_4k_5k_6k_7 + k_2k_7k_{-3}k_{-4}k_{-5} + k_2k_{-3}k_{-4}k_{-5}k_{-6}}{\Delta}$$

Eq. S3 P2

$$\frac{[B-C_{1b}:T:F]}{E_T} = \frac{k_2k_3k_5k_6k_7 + k_2k_3k_6k_7k_{-4} + k_2k_3k_7k_{-4}k_{-5} + k_2k_3k_{-4}k_{-5}k_{-6} + 0\cdot k_3k_{-6}k_{-5}k_{-4} + 0\cdot k_{-2}k_{-4}k_{-5}k_{-6}}{\Delta}$$
$$= \frac{k_2k_3k_5k_6k_7 + k_2k_3k_6k_7k_{-4} + k_2k_3k_7k_{-4}k_{-5} + k_2k_3k_{-4}k_{-5}k_{-6}}{\Delta}$$

Eq. S4 P3

$$\frac{[B-C_{1c}:T:F]}{E_T} = \frac{k_2k_3k_5k_6k_7 + k_2k_3k_4k_7k_{-5} + k_2k_3k_4k_{-5}k_{-6} + 0\cdot k_{-3}k_{-2}k_{-5}k_{-6} + 0\cdot k_{-2}k_{-6}k_{-5}k_4 + 0\cdot k_3k_4k_{-5}k_{-6}}{\Delta}$$
$$= \frac{k_2k_3k_5k_6k_7 + k_2k_3k_4k_7k_{-5} + k_2k_3k_4k_{-5}k_{-6}}{\Delta}$$

Eq. S5 P4

$$\frac{[B-C{:}T^{\cdot}{:}F]}{E_T} = \frac{k_2k_3k_5k_6k_7 + k_2k_3k_4k_5k_{-6} + 0\cdot k_{-4}k_{-3}k_{-2}k_{-6} + 0\cdot k_{-3}k_{-2}k_{-6}k_5 + 0\cdot k_{-2}k_{-6}k_4k_5 + 0\cdot k_{-6}k_3k_4k_5}{\Delta} = \frac{k_2k_3k_5k_6k_7 + k_2k_3k_4k_5k_{-6}}{\Delta}$$

Eq. S6 P5

$$\frac{[B-C{:}BS^{\cdot}]}{E_T} = \frac{k_2k_3k_4k_5k_6 + 0\cdot k_{-5}k_{-4}k_{-3}k_{-2} + 0\cdot k_6k_{-4}k_{-3}k_{-2} + 0\cdot k_5k_6k_{-3}k_{-2} + 0\cdot k_6k_5k_4k_{-2} + 0\cdot k_3k_4k_5k_6}{\Delta} = \frac{k_2k_3k_4k_5k_6}{\Delta}$$

As at the substrate saturation conditions, the total enzyme concentration equals the sum of the individual species we can show that Δ is a sum of all nominators of all four equations.

Eq. S7

$$\frac{[B-G^{\cdot}{:}T{:}F]}{E_T} + \frac{[B-C_{1a}{:}T{:}F]}{E_T} + \frac{[B-C_{1b}{:}T{:}F]}{E_T} + \frac{[B-C_{1c}{:}T{:}F]}{E_T} + \frac{[B-C{:}T^{\cdot}{:}F]}{E_T} + \frac{[B-C{:}BS^{\cdot}]}{E_T} = 1$$

Eq. S8

$$\Delta = k_2k_3k_4k_5k_6 + k_2k_3k_4k_5k_7 + k_2k_3k_4k_5k_{-6} + k_2k_3k_4k_6k_7 + k_2k_3k_4k_7k_{-5} + k_2k_3k_4k_{-5}k_{-6} + k_2k_3k_5k_6k_7 + k_2k_3k_6k_7k_{-4} + k_2k_3k_7k_{-4}k_{-5} + k_2k_3k_{-4}k_{-5}k_{-6} + k_2k_4k_5k_6k_7 + k_2k_5k_6k_7k_{-3} + k_2k_6k_7k_{-3}k_{-4} + k_2k_{73}k_{-3}k_{-4}k_{-5} + k_2k_{-3}k_{-4}k_{-5}k_{-6} + k_3k_4k_5k_6k_7 + k_4k_5k_6k_7k_{-2} + k_5k_6k_7k_{-2}k_{-3} + k_6k_7k_{-2}k_{-3}k_{-4} + k_7k_{-2}k_{-3}k_{-4}k_{-5} + k_{-2}k_{-3}k_{-4}k_{-5}k_{-6}$$

The observed in the experiment reaction velocity is the rate of product formation. In that case (assuming a low concentration of the product) this will depend on the concentration of the enzyme before the irreversible step:

Eq. S9

$$V = k_7[BSS-C{:}BS^{\cdot}]$$

Combining Eq 7 with Eq. 4 we get:

Eq. S10

$$V = \frac{k_2k_3k_4k_5k_6k_7E_T}{\begin{gathered}k_2k_3k_4k_5k_6 + k_2k_3k_4k_5k_7 + k_2k_3k_4k_5k_{-6} + k_2k_3k_4k_6k_7 + k_2k_3k_4k_7k_{-5} + k_2k_3k_4k_{-5}k_{-6} + k_2k_3k_5k_6k_7 \\ +k_2k_3k_6k_7k_{-4} + k_2k_3k_7k_{-4}k_{-5} + k_2k_3k_{-4}k_{-5}k_{-6} + k_2k_4k_5k_6k_7 + k_2k_5k_6k_7k_{-3} + k_2k_6k_7k_{-3}k_{-4} \\ +k_2k_7k_{-3}k_{-4}k_{-5} + k_2k_{-3}k_{-4}k_{-5}k_{-6} + k_3k_4k_5k_6k_7 + k_4k_5k_6k_7k_{-2} + k_5k_6k_7k_{-2}k_{-3} + k_6k_7k_{-2}k_{-3}k_{-4} \\ +k_7k_{-2}k_{-3}k_{-4}k_{-5} + k_{-2}k_{-3}k_{-4}k_{-5}k_{-6}\end{gathered}}$$

The obtained equation yields the correct unit of the reaction rate [M s^{-1}]

The V/E_T value can be calculated using the kinetic constants derived from pro*R* reaction pathway with unlabelled or labelled toluene thus enabling estimation of the relative rates and calculation of the kinetic isotope effect.

In order to estimate the $^D(V/K)$ effect coming from competitive isotope experiment one has to divide the derived equation for V by K_m, which is assumed to be:

Eq. S11

$$K_m = \frac{k_{-1} + k_{cat}}{k_1}$$

Eq. S12

$$\frac{k_{cat}}{K_m} = \frac{1}{(k_{-1} + k_{cat})} \frac{k_1k_2k_3k_4k_5k_6k_7}{\begin{gathered}k_2k_3k_4k_5k_6 + k_2k_3k_4k_5k_7 + k_2k_3k_4k_5k_{-6} + k_2k_3k_4k_6k_7 + k_2k_3k_4k_7k_{-5} + k_2k_3k_4k_{-5}k_{-6} + k_2k_3k_5k_6k_7 \\ +k_2k_3k_6k_7k_{-4} + k_2k_3k_7k_{-4}k_{-5} + k_2k_3k_{-4}k_{-5}k_{-6} + k_2k_4k_5k_6k_7 + k_2k_5k_6k_7k_{-3} + k_2k_6k_7k_{-3}k_{-4} \\ +k_2k_7k_{-3}k_{-4}k_{-5} + k_2k_{-3}k_{-4}k_{-5}k_{-6} + k_3k_4k_5k_6k_7 + k_4k_5k_6k_7k_{-2} + k_5k_6k_7k_{-2}k_{-3} + k_6k_7k_{-2}k_{-3}k_{-4} \\ +k_7k_{-2}k_{-3}k_{-4}k_{-5} + k_{-2}k_{-3}k_{-4}k_{-5}k_{-6}\end{gathered}}$$

$$= \frac{k_1k_2k_3k_4k_5k_6k_7}{k_2k_3k_4k_5k_6k_7 + k_{-1}\left(\begin{gathered}k_2k_3k_4k_5k_6 + k_2k_3k_4k_5k_7 + k_2k_3k_4k_5k_{-6} + k_2k_3k_4k_6k_7 + k_2k_3k_4k_7k_{-5} + k_2k_3k_4k_{-5}k_{-6} + k_2k_3k_5k_6k_7 \\ +k_2k_3k_6k_7k_{-4} + k_2k_3k_7k_{-4}k_{-5} + k_2k_3k_{-4}k_{-5}k_{-6} + k_2k_4k_5k_6k_7 + k_2k_5k_6k_7k_{-3} + k_2k_6k_7k_{-3}k_{-4} \\ +k_2k_7k_{-3}k_{-4}k_{-5} + k_2k_{-3}k_{-4}k_{-5}k_{-6} + k_3k_4k_5k_6k_7 + k_4k_5k_6k_7k_{-2} + k_5k_6k_7k_{-2}k_{-3} + k_6k_7k_{-2}k_{-3}k_{-4} \\ +k_7k_{-2}k_{-3}k_{-4}k_{-5} + k_{-2}k_{-3}k_{-4}k_{-5}k_{-6}\end{gathered}\right)}$$

The whole reaction extended model

Assumption:

The last irreversible step is product release. The binding of the substrates does not influence the k_{cat}.

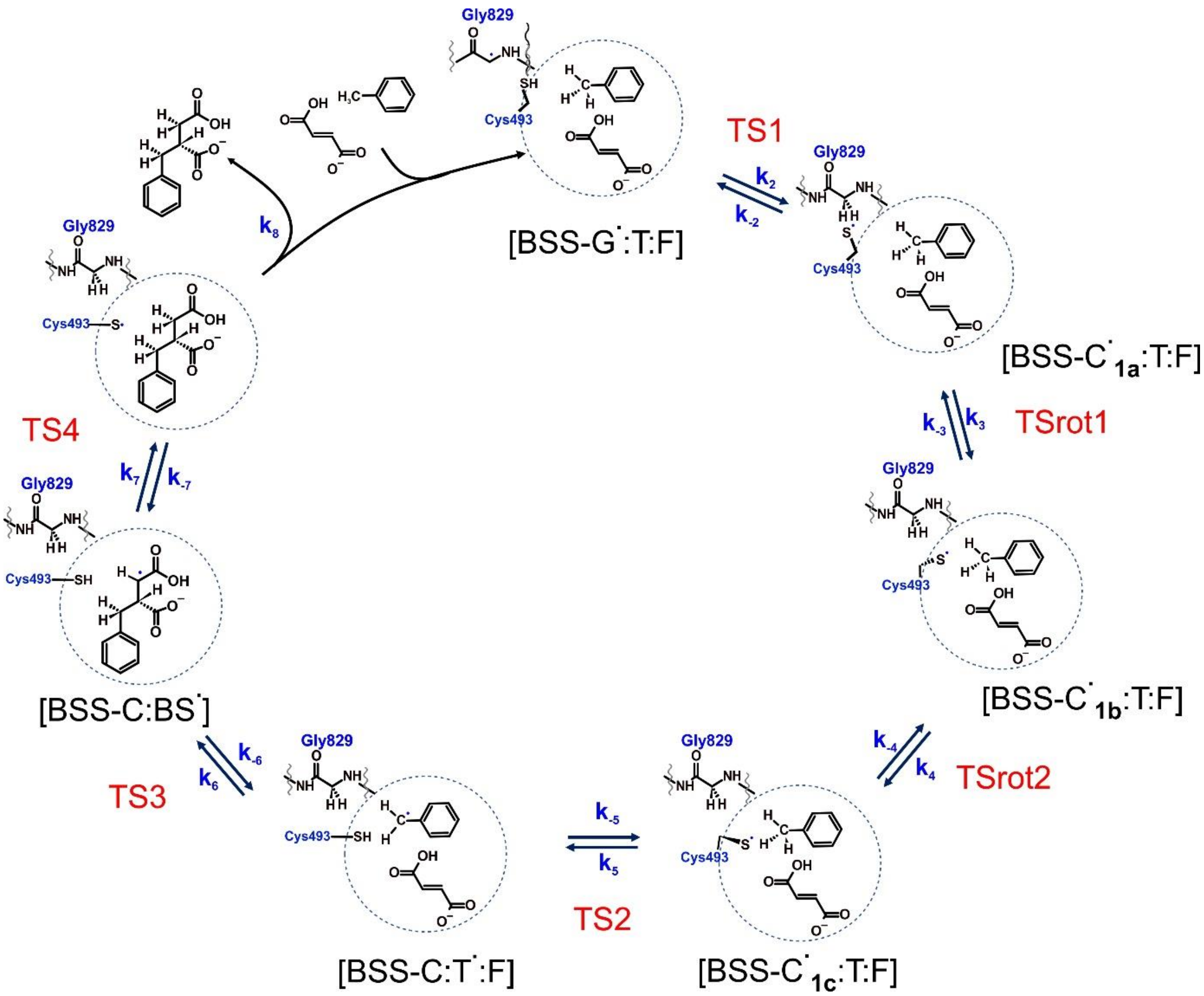

Scheme S2. The schematic representation of the whole reaction leading to the formation of benzylsuccinate assuming the irreversibility of the benzylsuccinate radical quenching step and no kinetic limitation from substrates binding/product release steps. [BSS-G·:T:F] – BSS with glycyl radical in complex with toluene and fumarate, [BSS-C·:T:F] – BSS with cysteinyl radical in complex with toluene and fumarate, [BSS-C:T·:F] – BSS with cysteine in complex with benzyl radical and fumarate, [BSS-C:BS·] – BSS with cysteine in complex with benzylsuccinyl radical, [BSS-C·:BS] with cysteine radical in complex with benzylsuccinate

The equation was derived using algebraic method in Jupiter notebook attached as SI.

Eq. S13

$$V = k_8[B - C^{\cdot}: BS] =$$

$$= \frac{k_2k_3k_4k_5k_6k_7k_8E_T}{\begin{gathered}k_2k_3k_4k_5k_6k_7 + k_2k_3k_4k_5k_6k_8 + k_2k_3k_4k_5k_6k_{-7} + k_2k_3k_4k_5k_7k_8 + k_2k_3k_4k_5k_8k_{-6} + k_2k_3k_4k_5k_{-6}k_{-7} + k_2k_3k_4k_6k_7k_8 \\ +k_2k_3k_4k_7k_8k_{-5} + k_2k_3k_4k_8k_{-5}k_{-6} + k_2k_3k_4k_{-5}k_{-6}k_{-7} + k_2k_3k_5k_6k_7k_8 + k_2k_3k_6k_7k_8k_{-4} + k_2k_3k_7k_8k_{-4}k_{-5} \\ +k_2k_3k_8k_{-4}k_{-5}k_{-6} + k_2k_3k_{-4}k_{-5}k_{-6}k_{-7} + k_2k_4k_5k_6k_7k_8 + k_2k_5k_6k_7k_8k_{-3} + k_2k_6k_7k_8k_{-3}k_{-4} + k_2k_7k_8k_{-3}k_{-4}k_{-5} \\ +k_2k_8k_{-3}k_{-4}k_{-5}k_{-6} + k_2k_{-3}k_{-4}k_{-5}k_{-6}k_{-7} + k_3k_4k_5k_6k_7k_8 + k_4k_5k_6k_7k_8k_{-2} + k_5k_6k_7k_8k_{-2}k_{-3} + k_6k_7k_8k_{-2}k_{-3}k_{-4} + \\ +k_7k_8k_{-2}k_{-3}k_{-4}k_{-5} + k_8k_{-2}k_{-3}k_{-4}k_{-5}k_{-6} + k_{-2}k_{-3}k_{-4}k_{-5}k_{-6}k_{-7}\end{gathered}}$$

HDX leading to d_1-benzylsuccinate – alpha pathway

Assumption:

Upon formation of d_1- benzylsuccinate (BS) it is released to the solvent and due to its very low concentration with respect to the unsubstituted BS the reaction is irreversible. The BS concentration is so high that the enzyme saturated by the product and binding of the product does not influence the overall kinetics.

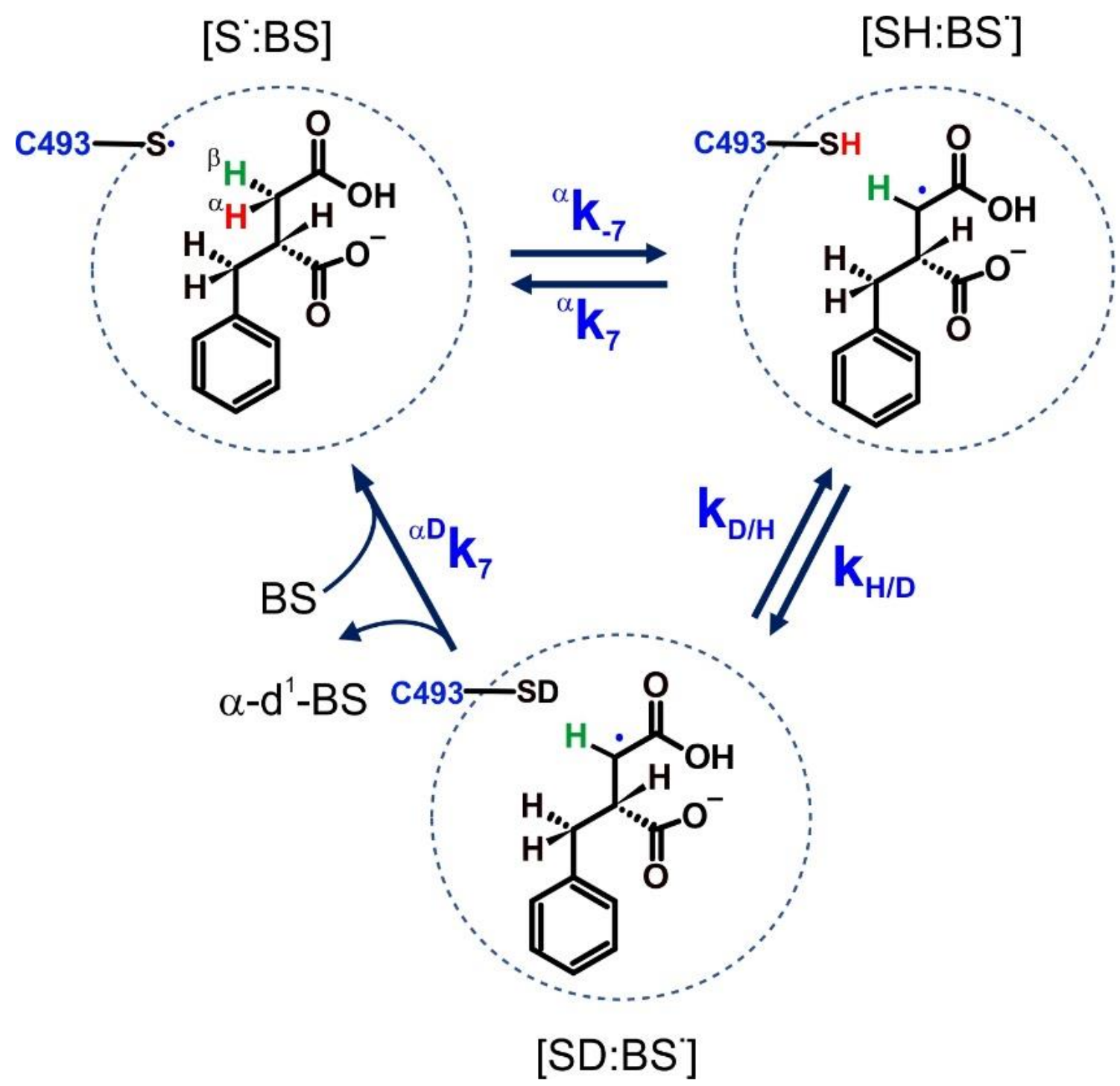

Scheme S3. The schematic representation of H/D exchange in the BSS:BS complex during incubation in D_2O leading to the production of d_1-benzylsuccinate assuming irreversibility of the D transfer to benzylsuccinyl radical and no kinetic limitation by product binding/release. [S˙:BS] – BSS with radical Cys in complex with benzylsuccinate, [SH:BS˙] – BSS with Cys-SH in complex with benzylsuccinyl radical, [SD:BS˙] – BSS with Cys-SD in complex with benzylsuccinyl radical

Eq. S14

$$\frac{[SD:BS^{\cdot}]}{E_T} = \frac{0 \cdot k_{H/D} + 0 \cdot {}^{\alpha}k_7 + {}^{\alpha}k_{-7}k_{H/D}}{\Delta} = \frac{{}^{\alpha}k_{-7}k_{H/D}}{\Delta}$$

Eq. S15

$$\frac{[S^{\cdot}:BS]}{E_T} = \frac{k_{H/D}{}^{\alpha D}k_7 + {}^{\alpha D}k_7{}^{\alpha}k_7 + k_{D/H}{}^{\alpha}k_7}{\Delta}$$

Eq. S16

$$\frac{[SH:BS^{\cdot}]}{E_T} = \frac{0 \cdot k_{D/H} + {}^{\alpha D}k_7{}^{\alpha}k_{-7} + k_{D/H}{}^{\alpha}k_{-7}}{\Delta}$$

Eq. S17

$$\Delta = 0 \cdot k_{H/D} + 0 \cdot {}^{\alpha}k_7 + {}^{\alpha}k_{-7}k_{H/D} + k_{H/D}{}^{\alpha D}k_7 + {}^{\alpha D}k_7{}^{\alpha}k_7 + k_{D/H}{}^{\alpha}k_7 + 0 \cdot k_{\frac{D}{H}} + {}^{\alpha D}k_7{}^{\alpha}k_{-7} + k_{\frac{D}{H}}{}^{\alpha}k_{-7} = $$
$$= {}^{\alpha}k_{-7}k_{H/D} + k_{H/D}{}^{\alpha D}k_7 + {}^{\alpha D}k_7{}^{\alpha}k_7 + k_{D/H}{}^{\alpha}k_7 + {}^{\alpha D}k_7{}^{\alpha}k_{-7} + k_{D/H}{}^{\alpha}k_{-7}$$

Eq. S18

$$V^{\alpha d1} = {}^{\alpha D}k_7[SD:BS^{\cdot}]$$

Eq. S19

$$V^{\alpha d1} = \frac{{}^{\alpha D}k_7{}^{\alpha}k_{-7}k_{H/D}[E_T]}{{}^{\alpha}k_{-7}k_{H/D} + k_{H/D}{}^{\alpha D}k_7 + {}^{\alpha D}k_7{}^{\alpha}k_7 + k_{D/H}{}^{\alpha}k_7 + {}^{\alpha D}k_7{}^{\alpha}k_{-7} + k_{D/H}{}^{\alpha}k_{-7}}$$

HDX leading to d_1-benzylsuccinate – beta pathway

Assumption:

Upon formation of d_1- benzylsuccinate (BS) it is released to the solvent and due to its very low concentration with respect to the unsubstituted BS the reaction is irreversible. The BS concentration is so high that the enzyme saturated by the product and binding of the product does not influence the overall kinetics.

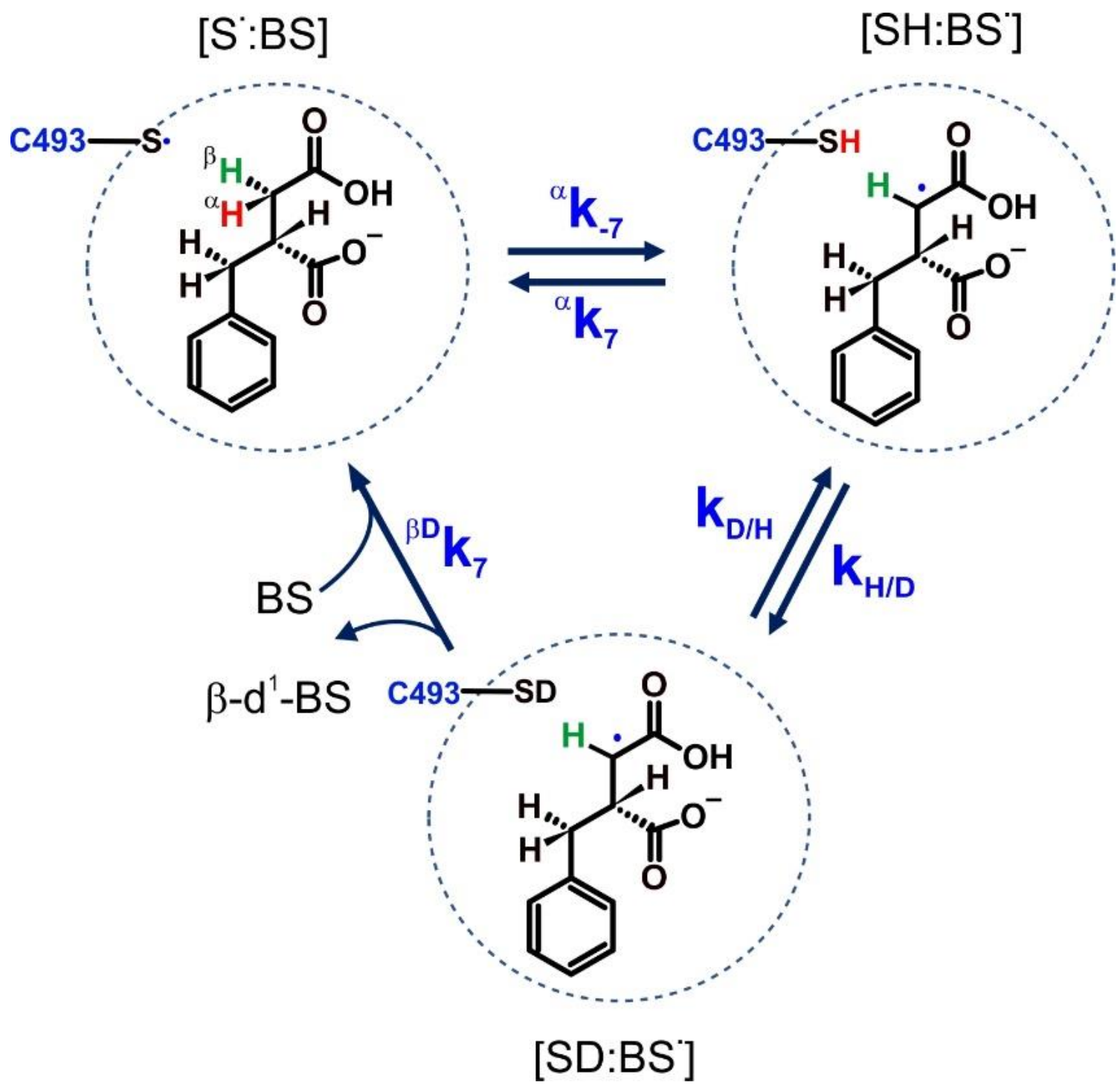

Scheme S4. The schematic representation of H/D exchange in the BSS:BS complex during incubation in D_2O leading to the production of d_1-benzylsuccinate assuming irreversibility of the D transfer to benzylsuccinyl radical and no kinetic limitation by product binding/release. [S·:BS] – BSS with radical Cys in complex with benzylsuccinate, [SH:BS·] – BSS with Cys-SH in complex with benzylsuccinyl radical, [SD:BS·] – BSS with Cys-SD in complex with benzylsuccinyl radical

Eq. S20

$$\frac{[SD{:}BS^{\cdot}]}{E_T} = \frac{0 \cdot k_{H/D} + 0 \cdot {}^{\alpha}k_7 + {}^{\alpha}k_{-7}k_{H/D}}{\Delta} = \frac{{}^{\alpha}k_{-7}k_{H/D}}{\Delta}$$

Eq. S21

$$\frac{[S^{\cdot}{:}BS]}{E_T} = \frac{k_{H/D}{}^{\beta D}k_7 + {}^{\beta D}k_7{}^{\alpha}k_7 + k_{D/H}{}^{\alpha}k_7}{\Delta}$$

Eq. S22

$$\frac{[SH{:}BS^{\cdot}]}{E_T} = \frac{0 \cdot k_{D/H} + {}^{\beta D}k_7{}^{\alpha}k_{-7} + k_{D/H}{}^{\alpha}k_{-7}}{\Delta}$$

Eq. S23

$$\Delta = 0 \cdot k_{H/D} + 0 \cdot {}^{\alpha}k_7 + {}^{\alpha}k_{-7}k_{H/D} + k_{H/D}{}^{\beta D}k_7 + {}^{\beta D}k_7{}^{\alpha}k_7 + k_{D/H}{}^{\alpha}k_7 + 0 \cdot k_{\frac{D}{H}} + {}^{\beta D}k_7{}^{\alpha}k_{-7} + k_{\frac{D}{H}}{}^{\alpha}k_{-7} =$$
$$= {}^{\alpha}k_{-7}k_{H/D} + k_{H/D}{}^{\beta D}k_7 + {}^{\beta D}k_7{}^{\alpha}k_7 + k_{D/H}{}^{\alpha}k_7 + {}^{\beta D}k_7{}^{\alpha}k_{-7} + k_{D/H}{}^{\alpha}k_{-7}$$

Eq. S24

$$V^{\beta d1} = {}^{\beta D}k_7[SD\!:BS^{\cdot}]$$

Eq. S25

$$V^{\beta d1} = \frac{{}^{\beta D}k_7\,{}^{\alpha}k_{-7}k_{H/D}[E_T]}{{}^{\alpha}k_{-7}k_{H/D} + k_{H/D}{}^{\beta D}k_7 + {}^{\beta D}k_7\,{}^{\alpha}k_7 + k_{D/H}{}^{\alpha}k_7 + {}^{\beta D}k_7\,{}^{\alpha}k_{-7} + k_{D/H}{}^{\alpha}k_{-7}}$$

HDX leading to d_2-benzylsuccinate – alpha pathway

Assumption:

Upon formation of d_2- benzylsuccinate it is released to the solvent and due to its very low concentration with respect to the unsubstituted benzylsuccinate, the reaction is irreversible. The benzylsuccinate concentration is so high that the enzyme is saturated by the product and binding of the product does not influence the overall kinetics.

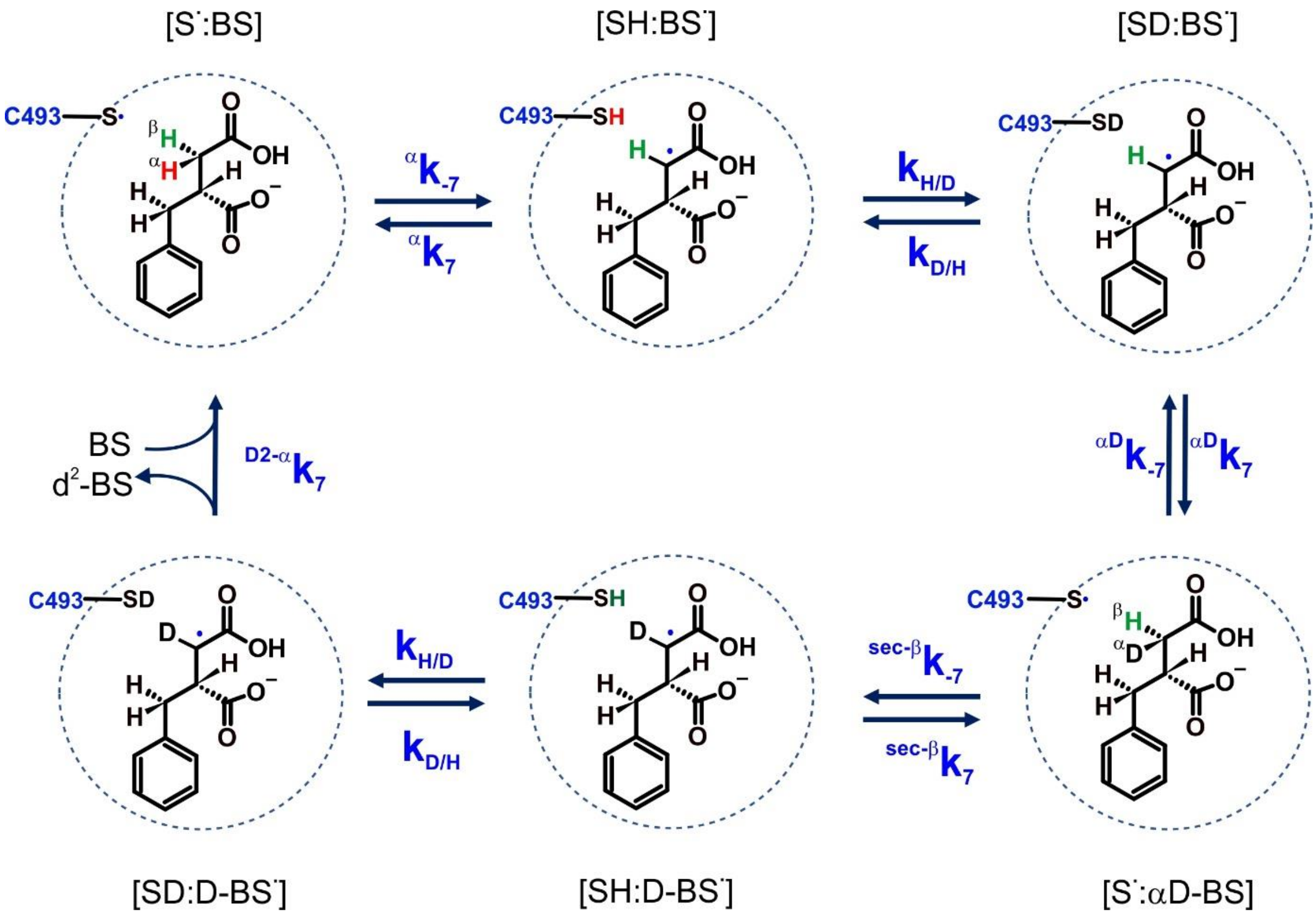

Scheme S5. The schematic representation of α pathway for H/D exchange in the BSS:BS complex during incubation in D_2O leading to the production of d_2-benzylsuccinate assuming irreversibility of the D transfer to benzylsuccinyl radical and no kinetic limitation by product binding/release. [S˙:BS] – BSS with radical Cys in complex with benzylsuccinate, [SH:BS˙] – BSS with Cys-SH in complex with benzylsuccinyl radical, [SD:BS˙] – BSS with Cys-SD in complex with benzylsuccinyl radical, [S˙:αD-BS] BSS

with radical Cys in complex with α-d_1-benzylsuccinate, [SH:D-BS$^{\cdot}$] - BSS with Cys-SH in complex with d_1-benzylsuccinyl radical, [SD:D-BS$^{\cdot}$] - BSS with Cys-SD in complex with d_1-benzylsuccinyl radical

Eq. S26

$$V = {}^{D2\alpha}k_7[SD{:}D - BS^{\cdot}]$$

Eq. S27

$$\frac{[SD{:}D{-}BS^{\cdot}]}{E_T} = \\ = \frac{0\cdot k_{\frac{H}{D}}^2\,{}^{\alpha D}k_7\,{}^{sec\beta}k_{-7} + {}^{\alpha D}k_7\,{}^{sec\beta}k_{-7}k_{\frac{H}{D}}\,{}^{\alpha}k_7\cdot 0 + {}^{sec\beta}k_{-7}k_{\frac{H}{D}}k_{\frac{D}{H}}\,{}^{\alpha}k_7\cdot 0 + k_{\frac{H}{D}}\,{}^{\alpha D}k_{-7}k_{\frac{D}{H}}\,{}^{\alpha}k_7\cdot 0 + {}^{\alpha}k_{-7}k_{\frac{H}{D}}^2\,{}^{\alpha D}k_7\,{}^{sec\beta}k_{-7}}{\Delta} \\ = \frac{{}^{\alpha}k_{-7}k_{H/D}^2\,{}^{\alpha D}k_7\,{}^{sec\beta}k_{-7}}{\Delta}$$

Eq. S28

$$\frac{[S^{\cdot}{:}BS]}{E_T} = \frac{k_{H/D}^2\,{}^{\alpha D}k_7\,{}^{sec\beta}k_{-7}\,{}^{D2\alpha}k_7 + {}^{\alpha}k_7\,{}^{\alpha D}k_7\,{}^{sec\beta}k_{-7}k_{H/D}\,{}^{D2\alpha}k_7 + k_{D/H}\,{}^{\alpha}k_7\,{}^{sec\beta}k_{-7}k_{H/D}\,{}^{D2\alpha}k_7}{\Delta} + \\ + \frac{{}^{\alpha D}k_{-7}k_{D/H}\,{}^{\alpha}k_7k_{H/D}\,{}^{D2\alpha}k_7 + {}^{sec\beta}k_7\,{}^{\alpha D}k_{-7}k_{D/H}\,{}^{\alpha}k_7\,{}^{D2\alpha}k_7 + k_{D/H}^2\,{}^{sec\beta}k_7\,{}^{\alpha D}k_{-7}\,{}^{\alpha}k_7}{\Delta}$$

Eq. S29

$$\frac{[SH{:}BS^{\cdot}]}{E_T} = \frac{k_{D/H}^2\,{}^{sec\beta}k_7\,{}^{\alpha D}k_{-7}\cdot 0 + {}^{\alpha D}k_7\,{}^{sec\beta}k_{-7}k_{H/D}\,{}^{D2\alpha}k_7\,{}^{\alpha}k_{-7} + k_{D/H}\,{}^{sec\beta}k_{-7}k_{H/D}\,{}^{D2\alpha}k_7\,{}^{\alpha}k_{-7}}{\Delta} + \\ \frac{{}^{\alpha D}k_{-7}k_{D/H}k_{H/D}\,{}^{D2\alpha}k_7\,{}^{\alpha}k_{-7} + {}^{sec\beta}k_7\,{}^{\alpha D}k_{-7}k_{D/H}\,{}^{D2\alpha}k_7\,{}^{\alpha}k_{-7} + {}^{\alpha}k_{-7}k_{D/H}^2\,{}^{sec\beta}k_7\,{}^{\alpha D}k_{-7}}{\Delta}$$

Eq. S30

$$\frac{[SD{:}BS^{\cdot}]}{E_T} = \frac{k_{H/D}\cdot 0\cdot k_{D/H}\,{}^{sec\beta}k_7\,{}^{\alpha D}k_{-7} + {}^{\alpha}k_7\cdot 0\cdot k_{D/H}\,{}^{sec\beta}k_7\,{}^{\alpha D}k_{-7} + {}^{sec\beta}k_{-7}k_{H/D}^2\,{}^{D2\alpha}k_7\,{}^{\alpha}k_{-7}}{\Delta} + \\ \frac{{}^{\alpha D}k_{-7}k_{H/D}^2\,{}^{D2\alpha}k_7\,{}^{\alpha}k_{-7} + {}^{sec\beta}k_7\,{}^{\alpha D}k_{-7}\,{}^{D2\alpha}k_7\,{}^{\alpha}k_{-7}k_{H/D} + {}^{\alpha}k_{-7}k_{H/D}k_{D/H}\,{}^{sec\beta}k_7\,{}^{\alpha D}k_{-7}}{\Delta}$$

$$\frac{[S^{\cdot}{:}\alpha DBS]}{E_T} = \frac{k_{H/D}\,{}^{\alpha D}k_7\cdot 0\cdot k_{D/H}\,{}^{sec\beta}k_7 + {}^{\alpha D}k_7\cdot 0\cdot k_{D/H}\,{}^{sec\beta}k_7 + k_{D/H}^2\,{}^{\alpha}k_7\cdot 0\cdot {}^{sec\beta}k_7}{\Delta} + \\ \frac{k_{H/D}^2\,{}^{D2\alpha}k_7\,{}^{\alpha}k_{-7}\,{}^{\alpha D}k_7 + {}^{sec\beta}k_7\,{}^{D2\alpha}k_7\,{}^{\alpha}k_{-7}k_{H/D}\,{}^{\alpha D}k_7 + {}^{\alpha}k_{-7}k_{H/D}\,{}^{\alpha D}k_7k_{D/H}\,{}^{sec\beta}k_7}{\Delta} \\ = \frac{k_{H/D}^2\,{}^{D2\alpha}k_7\,{}^{\alpha}k_{-7}\,{}^{\alpha D}k_7 + {}^{sec\beta}k_7\,{}^{D2\alpha}k_7\,{}^{\alpha}k_{-7}k_{H/D}\,{}^{\alpha D}k_7 + {}^{\alpha}k_{-7}k_{H/D}\,{}^{\alpha D}k_7k_{D/H}\,{}^{sec\beta}k_7}{\Delta}$$

$$\frac{[SH:DBS^{\cdot}]}{E_T} = \frac{0 \cdot k_{H/D}{}^{\alpha D}k_7{}^{sec\beta}k_{-7}k_{D/H} + {}^{\alpha D}k_7{}^{sec\beta}k_{-7}{}^{\alpha}k_7 \cdot 0 \cdot k_{D/H} + k_{D/H}{}^{\alpha}k_7 \cdot 0 \cdot {}^{sec\beta}k_{-7}k_{D/H}}{\Delta} + \frac{{}^{\alpha D}k_{-7}k_{D/H}{}^{\alpha}k_7 \cdot 0 \cdot k_{D/H} + {}^{D2\alpha}k_7{}^{\alpha}k_{-7}k_{H/D}{}^{\alpha D}k_7{}^{sec\beta}k_{-7} + k_{D/H}{}^{\alpha}k_{-7}k_{H/D}{}^{\alpha D}k_7{}^{sec\beta}k_{-7}}{\Delta}$$

$$= \frac{{}^{D2\alpha}k_7{}^{\alpha}k_{-7}k_{H/D}{}^{\alpha D}k_7{}^{sec\beta}k_{-7} + k_{D/H}{}^{\alpha}k_{-7}k_{H/D}{}^{\alpha D}k_7{}^{sec\beta}k_{-7}}{\Delta}$$

Eq. S31

$$\Delta = {}^{\alpha}k_{-7}k_{H/D}^2{}^{\alpha D}k_7{}^{sec\beta}k_{-7} + k_{H/D}^2{}^{\alpha D}k_7{}^{sec\beta}k_{-7}{}^{D2\alpha}k_7 + {}^{\alpha}k_7{}^{\alpha D}k_7{}^{sec\beta}k_{-7}k_{H/D}{}^{D2\alpha}k_7 + k_{D/H}{}^{\alpha}k_7{}^{sec\beta}k_{-7}k_{H/D}{}^{D2\alpha}k_7 + {}^{\alpha D}k_{-7}k_{D/H}{}^{\alpha}k_7k_{H/D}{}^{D2\alpha}k_7 + {}^{sec\beta}k_7{}^{\alpha D}k_{-7}k_{D/H}{}^{\alpha}k_7{}^{D2\alpha}k_7 + k_{D/H}^2{}^{sec\beta}k_7{}^{\alpha D}k_{-7}{}^{\alpha}k_7 + {}^{\alpha D}k_7{}^{sec\beta}k_{-7}k_{H/D}{}^{D2\alpha}k_7{}^{\alpha}k_{-7} + k_{D/H}{}^{sec\beta}k_{-7}k_{H/D}{}^{D2\alpha}k_7{}^{\alpha}k_{-7} + {}^{\alpha D}k_{-7}k_{D/H}k_{H/D}{}^{D2\alpha}k_7{}^{\alpha}k_{-7} + {}^{sec\beta}k_7{}^{\alpha D}k_{-7}k_{D/H}{}^{D2\alpha}k_7{}^{\alpha}k_{-7} + {}^{\alpha}k_{-7}k_{D/H}^2{}^{sec\beta}k_7{}^{\alpha D}k_{-7} + {}^{sec\beta}k_{-7}k_{H/D}^2{}^{D2\alpha}k_7{}^{\alpha}k_{-7} + {}^{\alpha D}k_{-7}k_{H/D}^2{}^{D2\alpha}k_7{}^{\alpha}k_{-7} + {}^{sec\beta}k_7{}^{\alpha D}k_{-7}{}^{D2\alpha}k_7{}^{\alpha}k_{-7}k_{H/D} + {}^{\alpha}k_{-7}k_{H/D}k_{D/H}{}^{sec\beta}k_7{}^{\alpha D}k_{-7} + k_{H/D}^2{}^{D2\alpha}k_7{}^{\alpha}k_{-7}{}^{\alpha D}k_7 + {}^{sec\beta}k_7{}^{D2\alpha}k_7{}^{\alpha}k_{-7}k_{H/D}{}^{\alpha D}k_7 + {}^{\alpha}k_{-7}k_{H/D}{}^{\alpha D}k_7k_{D/H}{}^{sec\beta}k_7 + {}^{D2\alpha}k_7{}^{\alpha}k_{-7}k_{H/D}{}^{\alpha D}k_7{}^{sec\beta}k_{-7} + k_{D/H}{}^{\alpha}k_{-7}k_{H/D}{}^{\alpha D}k_7{}^{sec\beta}k_{-7}$$

Eq. S32

$$V^{d2-\alpha} = \frac{{}^{\alpha}k_{-7}k_{H/D}^2{}^{\alpha D}k_7{}^{sec\beta}k_{-7}{}^{D2\alpha}k_7}{\Delta}E_T$$

HDX leading to d_2-benzylsuccinate – beta pathway

Assumption:

Upon the formation of d^2-benzylscuccinate (BS) it is released to the solvent and due to its very low concentration with respect to the unsubstituted BS the reaction is irreversible. The BS concentration is so high that the enzyme saturated by the product and binding of the product does not influence the overall kinetics.

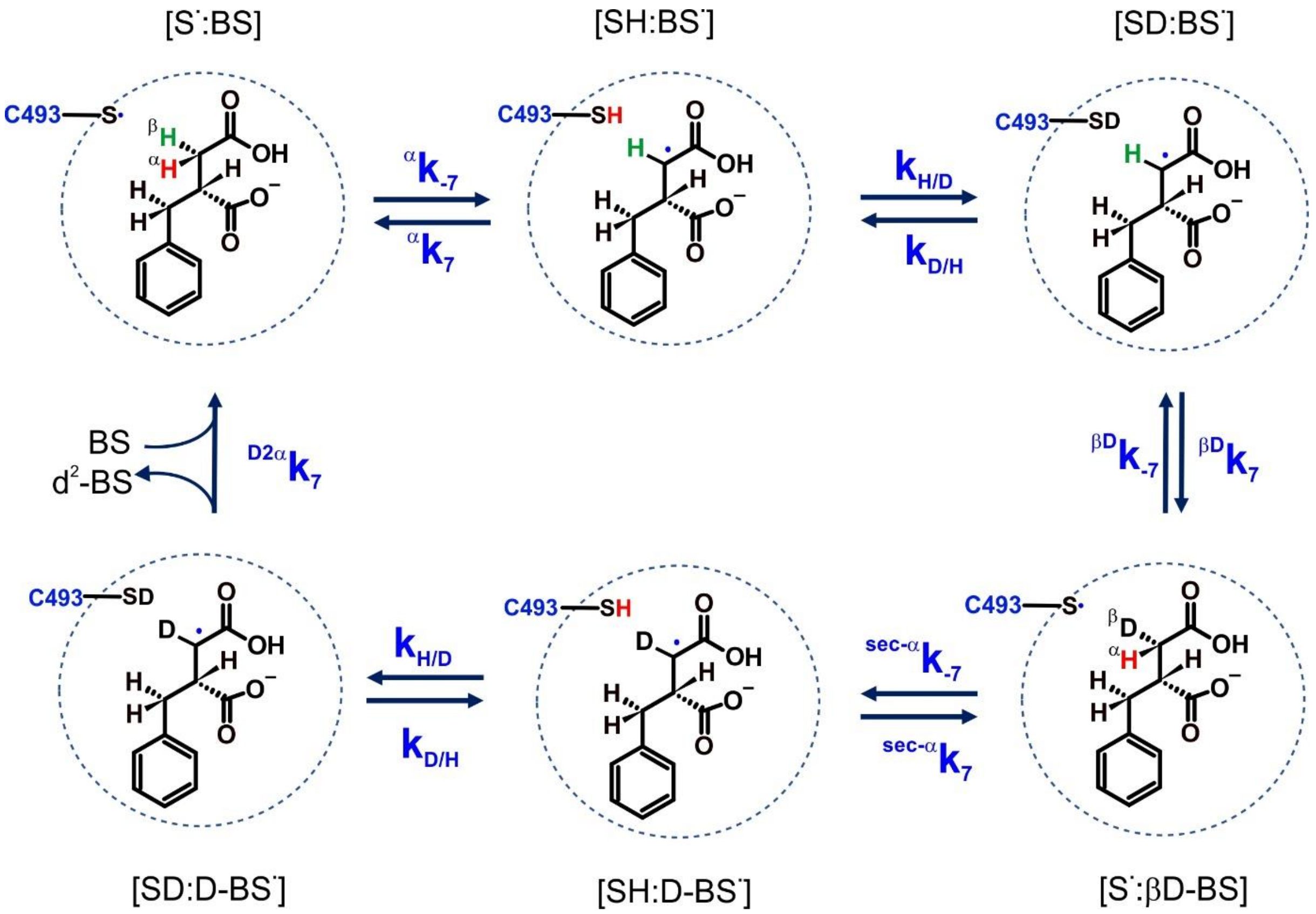

Scheme S6. The schematic representation of β pathway for H/D exchange in the BSS:BS complex during incubation in D_2O leading to the production of d_2-benzylsuccinate assuming irreversibility of the D transfer to benzylsuccinyl radical and no kinetic limitation by product binding/release. [S˙:BS] – BSS with radical Cys in complex with benzylsuccinate, [SH:BS˙] – BSS with Cys-SH in complex with benzylsuccinyl radical, [SD:BS˙] – BSS with Cys-SD in complex with benzylsuccinyl radical, [S˙:βD-BS] BSS with radical Cys in complex with β-d_1-benzylsuccinate, [SH:D-BS˙] - BSS with Cys-SH in complex with d_1-benzylsuccinyl radical, [SD:D-BS˙] - BSS with Cys-SD in complex with d_1-benzylsuccinyl radical

Eq. S33

$$V^{d2-\beta} = {}^{D2\alpha}k_7[SD\!:D - BS^{\cdot}]$$

Eq. S34

$$\frac{[SD\!:D-BS^{\cdot}]}{E_T} = \\ = \frac{0 \cdot k_{\frac{H}{D}}^2\, {}^{\beta D}k_7\, {}^{sec\alpha}k_{-7} + {}^{\beta D}k_7\, {}^{sec\alpha}k_{-7} k_{\frac{H}{D}}\, {}^{\alpha}k_7 \cdot 0 + {}^{sec\alpha}k_{-7} k_{\frac{H}{D}} k_{\frac{D}{H}}\, {}^{\alpha}k_7 \cdot 0 + k_{\frac{H}{D}}\, {}^{\beta D}k_{-7} k_{\frac{D}{H}}\, {}^{\alpha}k_7 \cdot 0 + {}^{\alpha}k_{-7} k_{H/D}^2\, {}^{\beta D}k_7\, {}^{sec\alpha}k_{-7}}{\Delta} \\ = \frac{{}^{\alpha}k_{-7} k_{H/D}^2\, {}^{\beta D}k_7\, {}^{sec\alpha}k_{-7}}{\Delta}$$

Eq. S35

$$\frac{[S^{\cdot}:BS]}{E_T} = \frac{k_{H/D}^2 {}^{\beta D}k_7 {}^{sec\alpha}k_{-7} {}^{D2\alpha}k_7 + {}^{\alpha}k_7 {}^{\beta D}k_7 {}^{sec\alpha}k_{-7} k_{H/D} {}^{D2\alpha}k_7 + k_{D/H} {}^{\alpha}k_7 {}^{sec\alpha}k_{-7} k_{H/D} {}^{D2\alpha}k_7}{\Delta} + $$
$$+ \frac{{}^{\beta D}k_{-7} k_{D/H} {}^{\alpha}k_7 k_{H/D} {}^{D2\alpha}k_7 + {}^{sec\alpha}k_7 {}^{\beta D}k_{-7} k_{D/H} {}^{\alpha}k_7 {}^{D2\alpha}k_7 + k_{D/H}^2 {}^{sec\alpha}k_7 {}^{\beta D}k_{-7} {}^{\alpha}k_7}{\Delta}$$

Eq. S36

$$\frac{[SH:BS^{\cdot}]}{E_T} = \frac{0 \cdot k_{D/H}^2 {}^{sec\alpha}k_7 {}^{\beta D}k_{-7} + {}^{\beta D}k_7 {}^{sec\alpha}k_{-7} k_{H/D} {}^{D2\alpha}k_7 {}^{\alpha}k_{-7} + k_{D/H} {}^{sec\alpha}k_{-7} k_{H/D} {}^{D2\alpha}k_7 {}^{\alpha}k_{-7}}{\Delta} +$$
$$\frac{{}^{\beta D}k_{-7} k_{D/H} k_{H/D} {}^{D2\alpha}k_7 {}^{\alpha}k_{-7} + {}^{sec\alpha}k_7 {}^{\beta D}k_{-7} k_{D/H} {}^{D2\alpha}k_7 {}^{\alpha}k_{-7} + {}^{\alpha}k_{-7} k_{D/H}^2 {}^{sec\alpha}k_7 {}^{\beta D}k_{-7}}{\Delta}$$

Eq. S37

$$\frac{[SD:BS^{\cdot}]}{E_T} = \frac{k_{H/D} \cdot 0 \cdot k_{D/H} {}^{sec\alpha}k_7 {}^{\beta D}k_{-7} + {}^{\alpha}k_7 \cdot 0 \cdot k_{D/H} {}^{sec\alpha}k_7 {}^{\beta D}k_{-7} + {}^{sec\alpha}k_{-7} k_{H/D}^2 {}^{D2\alpha}k_7 {}^{\alpha}k_{-7}}{\Delta} +$$
$$\frac{{}^{\beta D}k_{-7} k_{H/D}^2 {}^{D2\alpha}k_7 {}^{\alpha}k_{-7} + {}^{sec\alpha}k_7 {}^{\beta D}k_{-7} {}^{D2\alpha}k_7 {}^{\alpha}k_{-7} k_{H/D} + {}^{\alpha}k_{-7} k_{H/D} k_{D/H} {}^{sec\alpha}k_7 {}^{\beta D}k_{-7}}{\Delta}$$

Eq. S38

$$\frac{[S^{\cdot}:\beta DBS]}{E_T} = \frac{k_{H/D} {}^{\beta D}k_7 \cdot 0 \cdot k_{D/H} {}^{sec\alpha}k_7 + {}^{\alpha}k_7 \cdot 0 \cdot {}^{\beta D}k_7 k_{D/H} {}^{sec\alpha}k_7 + k_{H/D} {}^{\alpha}k_7 \cdot 0 \cdot k_{D/H} {}^{sec\alpha}k_7}{\Delta} +$$
$$\frac{k_{H/D}^2 {}^{D2\alpha}k_7 {}^{\alpha}k_{-7} {}^{\beta D}k_7 + {}^{sec\alpha}k_7 {}^{D2\alpha}k_7 {}^{\alpha}k_{-7} k_{H/D} {}^{\beta D}k_7 + {}^{\alpha}k_{-7} k_{H/D} {}^{\beta D}k_7 k_{D/H} {}^{sec\alpha}k_7}{\Delta}$$

Eq. S39

$$\frac{[SH:DBS^{\cdot}]}{E_T} = \frac{0 \cdot k_{H/D} {}^{\beta D}k_7 {}^{sec\alpha}k_{-7} k_{D/H} + {}^{\alpha}k_7 \cdot 0 {}^{\beta D}k_7 {}^{sec\alpha}k_{-7} k_{D/H} + k_{H/D} {}^{\alpha}k_7 \cdot 0 \cdot {}^{sec\alpha}k_{-7} k_{D/H}}{\Delta} +$$
$$\frac{{}^{\beta D}k_{-7} k_{H/D} {}^{\alpha}k_7 \cdot 0 \cdot k_{D/H} + {}^{D2\alpha}k_7 {}^{\alpha}k_{-7} k_{H/D} {}^{\beta D}k_7 {}^{sec\alpha}k_{-7} + k_{D/H} {}^{\alpha}k_{-7} k_{H/D} {}^{\beta D}k_7 {}^{sec\alpha}k_{-7}}{\Delta}$$
$$= \frac{{}^{D2\alpha}k_7 {}^{\alpha}k_{-7} k_{H/D} {}^{\beta D}k_7 {}^{sec\alpha}k_{-7} + k_{D/H} {}^{\alpha}k_{-7} k_{H/D} {}^{\beta D}k_7 {}^{sec\alpha}k_{-7}}{\Delta}$$

Eq. S40

$$V^{d2-\beta} = \frac{{}^{\alpha}k_{-7} k_{H/D}^2 {}^{\beta D}k_7 {}^{sec\alpha}k_{-7} {}^{D2\alpha}k_7}{\Delta} E_T$$

Eq. S41

$$\Delta = {}^{\alpha}k_{-7} k_{\frac{H}{D}}^2 {}^{\beta D}k_7 {}^{sec\alpha}k_{-7} +$$
$$k_{\frac{H}{D}}^2 {}^{\beta D}k_7 {}^{sec\alpha}k_{-7} {}^{D2\alpha}k_7 + {}^{\alpha}k_7 {}^{\beta D}k_7 {}^{sec\alpha}k_{-7} k_{\frac{H}{D}} {}^{D2\alpha}k_7 + k_{\frac{D}{H}} {}^{\alpha}k_7 {}^{sec\alpha}k_{-7} k_{\frac{H}{D}} {}^{D2\alpha}k_7 +$$
$$+ {}^{\beta D}k_{-7} k_{\frac{D}{H}} {}^{\alpha}k_7 k_{\frac{H}{D}} {}^{D2\alpha}k_7 + {}^{sec\alpha}k_7 {}^{\beta D}k_{-7} k_{\frac{D}{H}} {}^{\alpha}k_7 {}^{D2\alpha}k_7 + k_{\frac{D}{H}}^2 {}^{sec\alpha}k_7 {}^{\beta D}k_{-7} {}^{\alpha}k_7$$

$$+{}^{\beta D}k_7{}^{sec\alpha}k_{-7}k_{\frac{H}{D}}{}^{D2\alpha}k_7{}^{\alpha}k_{-7} + k_{\frac{D}{H}}{}^{sec\alpha}k_{-7}k_{\frac{H}{D}}{}^{D2\alpha}k_7{}^{\alpha}k_{-7} + {}^{\beta D}k_7{}^{sec\alpha}k_{-7}k_{\frac{H}{D}}{}^{D2\alpha}k_7{}^{\alpha}k_{-7}$$
$$+k_{\frac{D}{H}}{}^{sec\alpha}k_{-7}k_{\frac{H}{D}}{}^{D2\alpha}k_7{}^{\alpha}k_{-7} +$$
$$+{}^{sec\alpha}k_{-7}k_{H/D}^2{}^{D2\alpha}k_7{}^{\alpha}k_{-7} + {}^{\beta D}k_{-7}k_{H/D}^2{}^{D2\alpha}k_7{}^{\alpha}k_{-7} + {}^{sec\alpha}k_7{}^{\beta D}k_{-7}{}^{D2\alpha}k_7{}^{\alpha}k_{-7}k_{H/D}$$
$$+{}^{\alpha}k_{-7}k_{H/D}k_{D/H}{}^{sec\alpha}k_7{}^{\beta D}k_{-7} +$$
$$+k_{H/D}^2{}^{D2\alpha}k_7{}^{\alpha}k_{-7}{}^{\beta D}k_7 + {}^{sec\alpha}k_7{}^{D2\alpha}k_7{}^{\alpha}k_{-7}k_{H/D}{}^{\beta D}k_7 + {}^{\alpha}k_{-7}k_{H/D}{}^{\beta D}k_7k_{D/H}{}^{sec\alpha}k_7$$
$$+k_{H/D}^2{}^{D2\alpha}k_7{}^{\alpha}k_{-7}{}^{\beta D}k_7 + {}^{sec\alpha}k_7{}^{D2\alpha}k_7{}^{\alpha}k_{-7}k_{H/D}{}^{\beta D}k_7 + {}^{\alpha}k_{-7}k_{H/D}{}^{\beta D}k_7k_{D/H}{}^{sec\alpha}k_7$$
$$+{}^{D2\alpha}k_7{}^{\alpha}k_{-7}k_{H/D}{}^{\beta D}k_7{}^{sec\alpha}k_{-7} + k_{D/H}{}^{\alpha}k_{-7}k_{H/D}{}^{\beta D}k_7{}^{sec\alpha}k_{-7}$$

RESULTS

BSS sequence

MNDIASAKVLEYKGKTLKFTPEDPAEAKIPADELHEHLQKPSTARTKRLKERCRWKHASAGEFIEKSVTAGIERMRYL
TEAHKASEGQPEAIRRALGLANVLNKSTLVLQEDEFIVGYHAEDPNMFPLYPELSHMAVQDYLRSDYSPQPADEAA
AINEYWKPHSLQSKCQPYFDPADLGRMYQVSSMEAPSFASGYNSIVPPYETVLEDGLLARIRLAEKHIAEAQAEMST
FPWDGTKGLDNIAKIDNWKAMVIACKAVISWARRQGRLCKIVAENFETDPKRQADLLEIADICQRVPAEPCKGLKD
AMQAKFFTFLICHAIERYASGYAQKEDTLLWPYYKASVVDKKFQPMDHMGAVELVEMERLKISEHGAGKSRAYREI
FPGSNDLFILTVGGTNAKGEDACNDMTDAILEAAKRIRTAEPSIVFRYSQKNRDKTLRWVFECIRDGLGYPSIKHDEI
GTEQMKEYAKFSLNGNGATDEEAHNWVNVLCMSPGIHGRRKTQKTRSEGGGSIFPAKLLEITLNDGYDWSYADM
QLGPKTGDLSSLKTFDDVWEAFRKQYQYAINLCISSKDVSRYFEQRFLQMPFVSAIDDGCMELGKDACALSEQPNG
WHNPITTIVAANSLVAIKKLVFEEKKYTLEKLSQALKANWEGFEEMRVDFKRAPKWGNDDDYADGIITRFYEEIIGGE
MRKITNYSGGPVMPTGQAVGLYMEVGSRTGPTPDGRFGGEAADDGGISPYMGTDKKGPTAVLRSVSKVQKNQK
ANLLNQRLSVPIMRSAHGFEIWNSYMKTWHDLNIDHVQFNVVSTDEMRAAQREPEKHHDLIVRVSGYSARFVDIP
TYGQNTIIARQEQDFSASDLEFLNVEI

BSS beta subunit

MQAEKSLQNQPHTEAGTARPCRSCKWQTPDPTDPHRGQCTANRHAMGGVWKRWLRDVENTTCARHEEGKL

BSS gamma subunit

METTTCKQCANFFPVPPGADDYEAGKADCVRQKEDEKGKYWLSRPIFENSTQCEAFCAKRSFRDHV

BSS activating enzyme

MKIPLVTEIQRFSLQDGPGIRTTIFLKGCPLRCPWCHNPETQDVRQEFYYYPARCVGCGRCVAVCPAGTSRLALNSD
GRTTVEIDRTDCQRCMRCVAACLTDARTAVGQRMSVEEILREVLSDSLFYRNSGGGVTISGGDPLFHPAFTLELARR
IKEHDVHVAIETSCFPKNWAVIEPLLDYVDLFIVDLKSLNSKKHEEVVGWPLQPILDNIEHLIQSRANIRIHIPVIPGFN
DSSRDFDDYISYLNRHATQLDGVDILNYHVYGEGKYLSLGREKEYQYLGVEENPPETVLPLAKGLKLAGIKSVTIGGLV
GITTDGHETIRNAGTGCIT

BSS kinetics with toluene

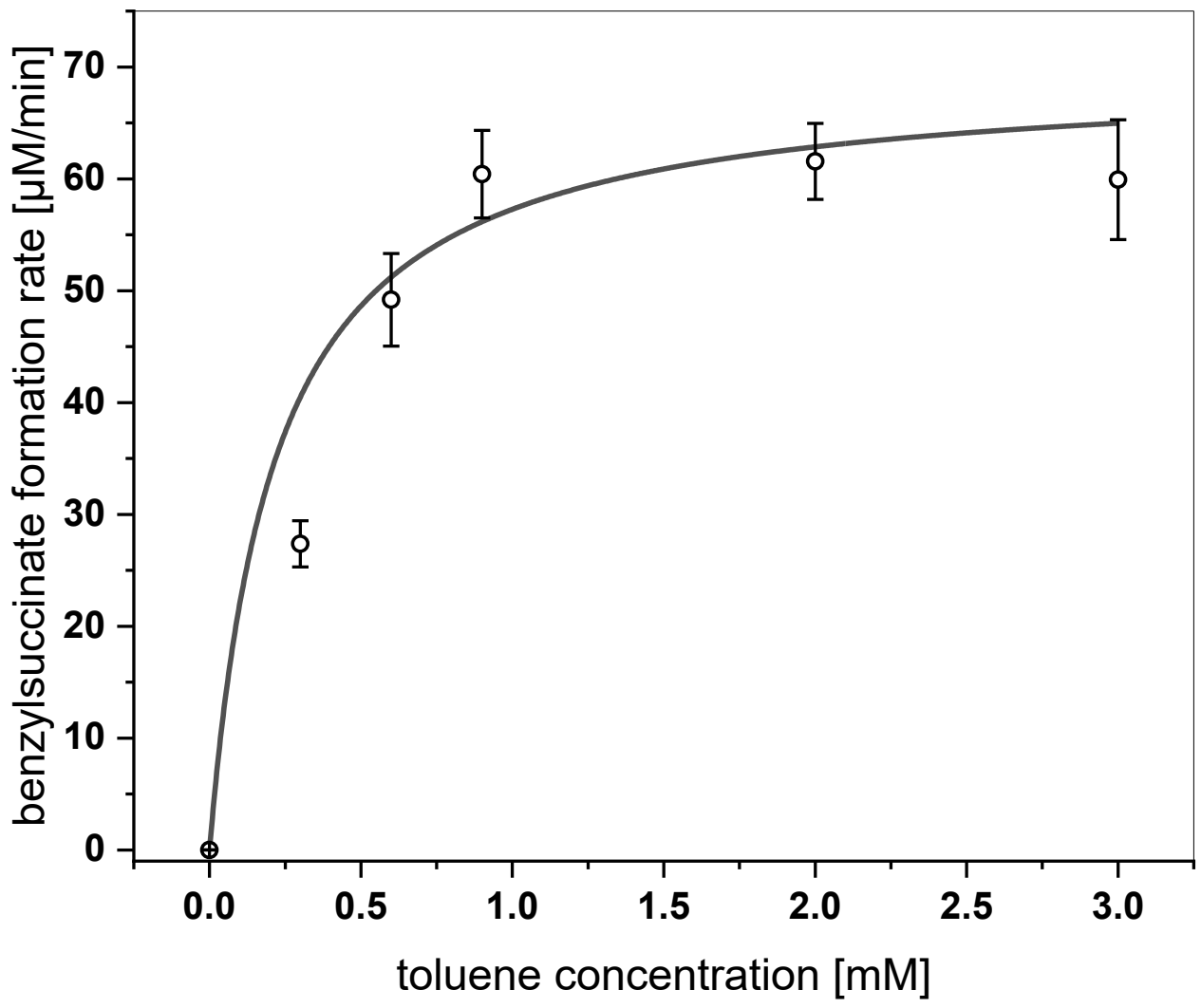

Figure S3. Benzylsuccinate synthase activity dependence on toluene concentration, at the constant concentration of sodium fumarate; data fitted to the Michaelis-Menten model.

V_{max} [µM min^{-1}]:	69.65183 ± 4.86822
K_m [mM]:	0.21604 ± 0.06782
Adj. R^2	0.90258

QM:MM energies

Table S4. Energies and vibration corrections (303 K, 1 atm., scaling factor 0.9806).

	E^{QM1} dzvp [Ha]	E^{QM1} tzvp [Ha]	E^{QM2} dzvp [Ha]	E^{QM2} tzvp [Ha]	ZPE [Ha]	Thermal [Ha]	H [Ha]	G [Ha]
**Step 1 – activation of Cys493**								
**ES**	-2365.55824493968	-2366.20961790382	-8363.75303975607	-8366.11453637253	24.0605620	25.64704	25.648	22.48973
**TS1**	-2365.53795032585	-2366.19090709356	-8363.73073384547	-8366.09067216491	24.059419	25.64436	25.64532	22.49091
**I1**	-2365.58193794600	-2366.23301809200	-8363.76789923912	-8366.13003621295	24.065249	25.6512	25.65216	22.4947
**Step 2 – rotation of Cys493**								
**I1a**	-2365.57973303777	-2366.23058662424	-8363.76536993320	-8366.12753031643	24.065646	25.651458	25.652417	22.496367
**TSrot1**	-2365.57973303777	-2366.23058662424	-8363.76536993320	-8366.12753031643	24.065426	25.650330	25.651290	22.497210
**I1b**	-2365.57348427022	-2366.22620228654	-8363.75321749086	-8366.11730157776	24.066847	25.651931	25.652890	22.498611
**TSrot2**	-2365.57683090189	-2366.22951329371	-8363.76295837319	-8366.12499923352	24.064214	25.649431	25.650391	22.494955
**I1c**	-2365.57827999697	-2366.23055406862	-8363.76600888837	-8366.12806032555	24.038382	25.625512	25.626472	22.467087
**Step 3 – activation of toluene**								
**I1a**	-2365.56870369175	-2366.22015505998	-8363.76724805284	-8366.12678460707	24.068848	25.65372	25.65468	22.49945
**I1c**	-2365.56646282554	-2366.21885058225	-8363.76453764535	-8366.12173925949	24.069516	25.65296	25.65392	22.50493
**TS2**	-2365.54112015292	-2366.19014262673	-8363.73779947531	-8366.09838162977	24.064383	25.64754	25.64849	22.50063
**I2**	-2365.55023224354	-2366.20132139113	-8363.74813172313	-8366.10911066041	24.066995	25.65113	25.65209	22.50072
**Step 4 - C-C bond formation**								
**I2**	-2365.45194508504	-2366.10068922705	-8363.65402355762	-8366.03305887327	24.071611	25.65317	25.65413	22.51325
**TS3**	-2365.42852539412	-2366.07341173620	-8363.63936883283	-8366.01487514838	24.07282	25.65307	25.65403	22.51626
**I3**	-2365.48143694255	-2366.11856395130	-8363.67678717914	-8366.04930408459	24.076855	25.65549	25.65645	22.52454
**Step 5 – quenching the radical benzylsuccinate intermediate**								
**$I3^{\alpha}$**	-2365.50700866888	-2366.14346375052	-8363.70579042290	-8366.07779821857	24.076265	25.655491	25.656451	22.521895
**$TS4^{\alpha}$**	-2365.48588258744	-2366.11938122718	-8363.68242424925	-8366.05242786375	24.073973	25.651857	25.652816	22.522758
**$E:P^{\alpha}$**	-2365.52336320995	-2366.15670556527	-8363.71865395157	-8366.08816044918	24.081349	25.659483	25.660443	22.528735

**I3$^{\beta}$**	-2365.52576542669	-2366.16263312239	-8363.73225598623	-8366.10008982158	24.079342	25.657178	25.658137	22.526298
**TS4 $^{\beta}$**	-2365.47538833495	-2366.11195044818	-8363.68210748019	-8366.05308283158	24.072954	25.651457	25.652417	22.518557
**E:P$^{\beta}$**	-2365.53603896205	-2366.16686854738	-8363.74803588762	-8366.11246640593	24.083004	25.660489	25.661448	22.530834
**Step 6 – transfer of the H atom between Gly and Cys for the E:P complex.**								
**E:Prot**	-2365.52559254147	-2366.15729078714	-8363.71785348512	-8366.08581950566	24.081333	25.659544	25.660504	22.528759
**E:P'**	-2365.52384603110	-2366.15562248325	-8363.71442505103	-8366.08357385082	24.08247	25.660248	25.661208	22.531462
**TS5**	-2365.47814153330	-2366.11109418045	-8363.67055202975	-8366.03996412632	24.076352	25.65328	25.654239	22.527184
**E:P-Gly$^{\cdot}$**	-2365.49737337207	-2366.13196334355	-8363.69288270337	-8366.06449495651	24.078949	25.656978	25.657937	22.527532

Table S5. Pro*R* pathway: differences in energies calculated for QM2 at B3LYP/6-311g+(2d,2p)/GD3:AMBER level of theory without (ΔE^{QM2} tzvp) or with thermal energy corrections ($\Delta E^{QM2+thermal}$ tzvp) calculated with respect to ES geometry; corrections – energy correction introduced to the reaction energy profile matching corresponding stationary points after conformational changes of the model; final energy profile ($\Delta E^{QM2+thermal}$ tzvp corrected)

[kJ/mol]	ΔE^{QM2} tzvp	$\Delta E^{QM2+thermal}$ tzvp	corrections	$\Delta E^{QM2+thermal}$ tzvp corrected
**Step 1 – activation of Cys493**				
**ES**	0.00	0.0	0	0.0
**TS1**	62.66	55.6	0	55.6
**I1**	-40.69	-29.8	0	-29.8
**Step 2 – rotation of Cys493**				
**I1a**	-34.1	-20.8	-7.26	-29.8
**TSrot1**	-7.3	8.2	-7.26	-5.9
**I1b**	-50.8	-40.4	-7.26	-45.2
**TSrot2**	-27.5	-19.8	-7.26	-28.5
**I1c**	-35.5	-33.26	-7.26	-34.7
**Step 2 – activation of toluene**				
**I1a**	-32.16	-14.6	-15.2	-29.8
**I1c**	-6.0	-3.4	-15.2	-18.5
**TS2**	46.3	43.7	-15.2	28.6
**I2**	31.8	25.0	-15.2	9.8
**Step 3 - C-C bond formation**				
**I2**	213.9	230.03	-220.20	9.8
**TS3**	261.7	277.49	-220.20	57.2
**I3**	171.3	193.46	-220.20	-26.7
**Step 4 – quenching the radical benzylsuccinate intermediate**				
**$I3^{\alpha}$**	96.5	118.6	-145.39	-26.7
**$TS4^{\alpha}$**	163.1	175.7	-145.39	30.3
**$I4^{\alpha}$**	69.2	101.9	-145.39	-43.5
**$I3^{\beta}$**	37.9	64.5	-91.30	-26.7
**$TS4^{\beta}$**	161.3	172.9	-91.30	81.7
**E:P**	5.4	40.7	-91.30	-50.5
**Step 5 – transfer of the H atom between Gly and Cys for the E:P complex.**				
**E:P_rot**	81.29	116.0	-153.1	-37.2
**TS5**	195.79	212.2	-153.1	59.0
**$E:P\text{-}Gly^{\cdot}$**	131.38	157.5	-153.1	4.3

Kinetic isotope effect

Table S6. Vibrational corrections calculated for toluene and d_8-toluene at 303 K, 1 atm. and a scaling factor of 0.9806.

	ZPE [Ha]	Thermal E [Ha]	H [Ha]	G [Ha]
**ES**	24.060562	25.647039	25.647999	22.489731
**ES d_8**	24.034850	25.622422	25.623382	22.462949
**TS1**	24.059419	25.644356	25.645315	22.490913
**TS1 d_8**	24.033692	25.619726	25.620686	22.464119
**I1**	24.065249	25.651198	25.652157	22.494700
**I1 d_8**	24.039525	25.626570	25.627529	22.467910
**I1a**	24.065646	25.651458	25.652417	22.496367
**I1a d_8**	24.039939	25.626845	25.627805	22.469590
**TSrot1**	24.065426	25.650330	25.651290	22.497210
**TSrot1 d_8**	24.039723	25.625719	25.626679	22.470434
**I1b**	24.066847	25.651931	25.652890	22.498611
**I1b**	24.066847	25.651931	25.652890	22.498611
**I1b d_8**	24.041121	25.627302	25.628261	22.471818
**TSrot2**	24.064214	25.649431	25.650391	22.494955
**TSrot2 d_8**	24.038476	25.624807	25.625766	22.468172
**I1c**	24.064108	25.650129	25.651089	22.493868
**I1c d_8**	24.038382	25.625512	25.626472	22.467087
**I1c***	24.069516	25.652960	25.653919	22.504934
**I1c d_8**	24.043781	25.628323	25.629283	22.478131
**TS2**	24.064383	25.647535	25.648494	22.500633
**TS2 d_8**	24.040425	25.624824	25.625784	22.475778
**I2**	24.066995	25.651131	25.652090	22.500720
**I2 d_8**	24.042313	25.627749	25.628709	22.474893
**I2**	24.071611	25.653174	25.654133	22.513249
**I2 d_8**	24.046967	25.629833	25.630793	22.487452
**TS3**	24.072820	25.653069	25.654028	22.516258
**TS3 d_8**	24.047858	25.629374	25.630333	22.490216
**I3**	24.076855	25.655490	25.656450	22.524540
**I3 d_8**	24.051479	25.631310	25.632269	22.498184
**I3**	24.076265	25.655491	25.656451	22.521895
**I3 d_8**	24.050888	25.631305	25.632264	22.495519
**TS4**	24.073973	25.651857	25.652816	22.522758
**TS4 d_8**	24.049555	25.628623	25.629582	22.497554

**E:P**	24.081349	25.659483	25.660443	22.528735
**E:P d_8**	24.055046	25.634321	25.635280	22.501628
**E:Prot**	24.082470	25.660248	25.661208	22.531462
**E:Prot d_8**	24.056155	25.635076	25.636035	22.504331
**TS5**	24.076352	25.653280	25.654239	22.527184
**TS5 d_8**	24.050029	25.628098	25.629058	22.500049
**E:P-Gly$^{\cdot}$**	24.078949	25.656978	25.657937	22.527532
**E:P-Gly$^{\cdot}$ d_8**	24.052637	25.631807	25.632767	22.500403

*I1c derived from TS2, different conformation from I1c from TSrot2

Table S7. Vibrational corrections calculated for benzylsuccinate, d_1-benzylsuccinate substituted at syn (α) or anti (β) position with respect to the phenyl ring, obtained at 303 K, 1 atm. and a scaling factor of 0.9806.

Step	ZPE [Ha]	Thermal E [Ha]	H [Ha]	G [Ha]
**Alpha transfer**				
**I3**	24.076265	25.655491	25.656451	22.521895
**d_1-I3**	24.073860	25.653270	25.654230	22.519200
**$d_{1\text{-sec}}$-I3**	24.073175	25.652586	25.653545	22.518667
**d_2-I3**	24.070771	25.650365	25.651324	22.515972
**TS4**	24.073973	25.651857	25.652816	22.522758
**d_1-TS4**	24.072575	25.650628	25.651588	22.521269
**$d_{1\text{-sec}}$-TS4**	24.070769	25.648816	25.649775	22.519446
**d_2-TS4**	24.069372	25.647589	25.648549	22.517960
**E:P**	24.080770	25.659112	25.660071	22.527795
**d_1- E:P**	24.077413	25.655886	25.656845	22.524342
**$d_{1\text{-sec}}$- E:P**	24.078103	25.656383	25.657342	22.525385
**d_1- E:P**	24.074723	25.653125	25.654084	22.521915
**Beta transfer**				
**β-I3**	24.079342	25.657178	25.658137	22.526298
**d_1-β-I3**	24.076930	25.654963	25.655923	22.523608
**$d_{1\text{-sec}}$-β-I3**	24.076270	25.654289	25.655249	22.523077
**d_2-β-I3**	24.073858	25.652075	25.653035	22.520388
**β-TS4**	24.072954	25.651457	25.652417	22.518557
**d_1-β-TS4**	24.071561	25.650237	25.651196	22.517070
**$d_{1\text{-sec}}$-β-TS4**	24.069722	25.648378	25.649337	22.515231
**d_2-β-TS4**	24.068334	25.647163	25.648123	22.513750
**β- E:P**	24.083004	25.660489	25.661448	22.530834
**d_1-β- E:P**	24.079764	25.657394	25.658353	22.527486
**$d_{1\text{-sec}}$-β- E:P**	24.079665	25.657281	25.658240	22.527395
**d_1-β-E:P**	24.076403	25.654156	25.655116	22.524033

Table S8. Kinetic constants and intrinsic kinetic isotope effects (iKIE) calculated for each step of the pro*R* reaction pathway for the highest level of theory and thermal energy corrections.

	**Δ(E^QM2 tzvp+Therm) [kJ/mol]**		**k [s⁻¹]**	**Γ(T)*kᵢ [s⁻¹]**	**iKIE**	**Δ(E^QM2 tzvp+Therm) [kJ/mol]**		**k [s⁻¹]**	**Γ(T)*kᵢ [s⁻¹]**	**iKIE**
		forward					reverse			
**ES**	0.00					29.78				
**ES d_8**	0.00					29.80				
**TS1**	55.61	k_2	1635	3104	**0.99**	85.39	k_{-2}	$1.20*10^{-2}$	$2.29*10^{-2}$	**1.0**
**TS1 d_8**	55.58	$^Dk_2$	1657	3146.7		85.38	$^Dk_{-2}$	$1.21*10^{-2}$	$2.29*10^{-2}$	
**I1**	-29.78					0.00				
**I1 d_8**	-29.80					0.00				
**I1a**	0.00					15.45				
**I1a d_8**	0.00					15.49				
**TSrot1**	23.89	k_3	$4.8*10^8$	$4.81*10^8$	**1.002**	39.35	k_{-3}	$1.04*10^6$	$1.04*10^6$	**1.02**
**TSrot1 d_8**	23.90	$^Dk_3$	$4.79*10^8$	$4.80*10^8$		39.39	$^Dk_{-3}$	$1.02*10^6$	$1.02*10^6$	
**I1b**	-15.45					0.00				
**I1b d_8**	-15.49					0.00				
**I1b**	0.00					-10.57				
**I1b d_8**	0.00					-10.60				
**TSrot2**	16.78	k_4	$8.10*10^9$	$8.13*10^9$	**1.01**	6.20	k_{-4}	$5.38*10^{11}$	$5.40*10^{11}$	**0.99**
**TSrot2 d_8**	16.79	$^Dk_4$	$8.05*10^9$	$8.09*10^9$		6.19	$^Dk_{-4}$	$5.42*10^{11}$	$5.44*10^{11}$	
**I1c**	10.57					0.00				
**I1c d_8**	10.60					0.00				
**I1c**	0.00					-28.35				
**I1c d_8**	0.00					-31.65				
**TS2**	47.08	k_5	$4.83*10^4$	$1.01*10^5$	**7.44**	18.73	k_{-5}	$3.73*10^9$	$7.83*10^9$	**2.0**
**TS2 d_8**	52.14	$^Dk5$	$6.49*10^3$	$1.06*10^4$		20.49	$^Dk_{-5}$	$1.85*10^9$	$3.03*10^9$	
**I2**	28.35					0.00				
**I2 d_8**	31.65					0.00				
**I2**	0.00					36.57				
**I2 d_8**	0.00					38.77				
**TS3**	47.47	k_6	$4.15*10^4$	$4.87*10^4$	**0.69**	84.04	k_{-6}	$2.06*10^{-2}$	$2.42*10^{-2}$	**1.7**
**TS3 d_8**	46.54	$^Dk_6$	$6.00*10^4$	$7.04*10^4$		85.31	$^Dk_{-6}$	$1.24*10^{-2}$	$1.46*10^{-2}$	
**I3**	-36.57					0.00				
**I3 d_8**	-38.77					0.00				
**I3**	0.00					16.73				
**I3 d8**	0.00					19.29				
**TS4**	57.07	k_7	917	2324	**2.70**	73.8	k_{-7}	1.2	3.0	**7.5**
**TS4 d_8**	59.57	$^Dk_7$	340	637		78.9	$^Dk_{-7}$	0.16	0.30	
**E:P**	-4.68					0.00				
**E:P d_8**	-7.25					0.00				
**E:P**	0.00					-41.51				
**E:P d_8**	0.00					-41.51				
**TS5**	96.20	k'_9	$1.64*10^{-4}$		**0.99**	54.70	k'_{-9}	2351		1.0
**TS5 d_8**	96.18	$^Dk'_9$	$1.66*10^{-4}$			54.67	$^Dk'_{-9}$	2378		
**E:P-Gly·**	41.51					0.00				
**E:P-Gly· d_8**	41.51					0.00				

Fitted values from the extended model eq. 18: k_1 4.66640 $M^{-1}\,s^{-1}$, $^{D}k_1$ 3.25829 $M^{-1}\,s^{-1}$, k_{-1} 1 s^{-1}, $^{D}k_{-1}$ 1.s^{-1} , k_8 2.341*10^{-2} s^{-1}, $^{D}k_8$ 2.341*10^{-2} s^{-1}

Approximation of transmission coefficients

The values of transmission coefficients (κ) were estimated based on the curvature of the transition state, which was approximated by the value of imaginary frequency (ν) associated with the particular transition state. We decided to assume κ values in the range of 0.4-0.95 with a fairly linear relationship between ν and κ. Furthermore, the processes associated with the transfer of deuteron were assumed to proceed with κ higher by the factor of 0.05

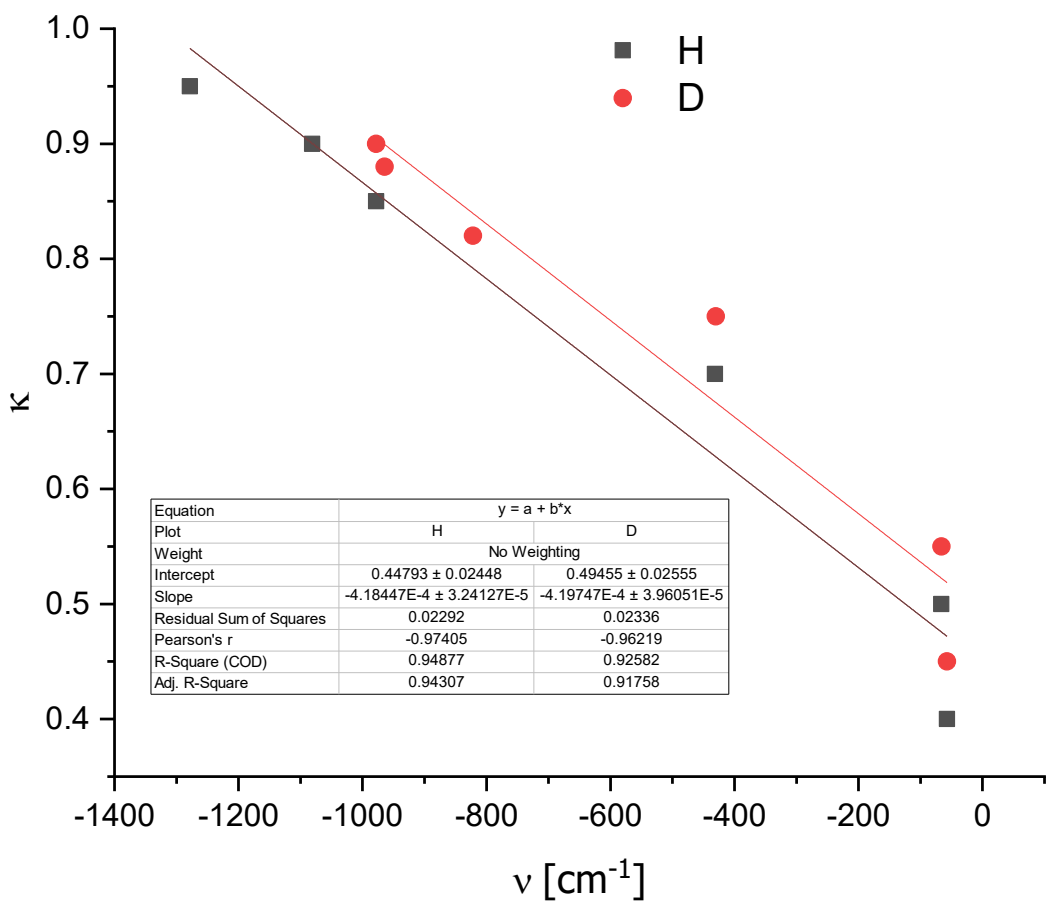

Figure S4. Relation of frequency at the transition state and the approximated transmission coefficient. The imaginary frequency was shown as a negative value.

Table S9. Values of transmission coefficients κ assumed for different kinetic constants based on the imaginary frequency of the transition state and type of nucleus being transferred.

Kinetic constant	ν H [cm^{-1}]	ν D[cm^{-1}]	κ H	κ D
k_2	-977.9	-977.9	0.85	0.90
k_{-2}	-977.9	-977.9	0.85	0.90
k_3	-57.2494	-57.2418	0.40	0.45
k_{-3}	-57.2494	-57.2418	0.40	0.45
k_4	-66.2945	-66.2109	0.50	0.55
k_{-4}	-66.2945	-66.2109	0.50	0.55
k_5	-1081.2	-821.6	0.90	0.82
k_{-5}	-1081.2	-821.6	0.90	0.82
k_6	-431.584	-429.91	0.70	0.75
k_{-6}	-431.584	-429.91	0.70	0.75
k_7	-1278.27	-964.138	0.95	0.88

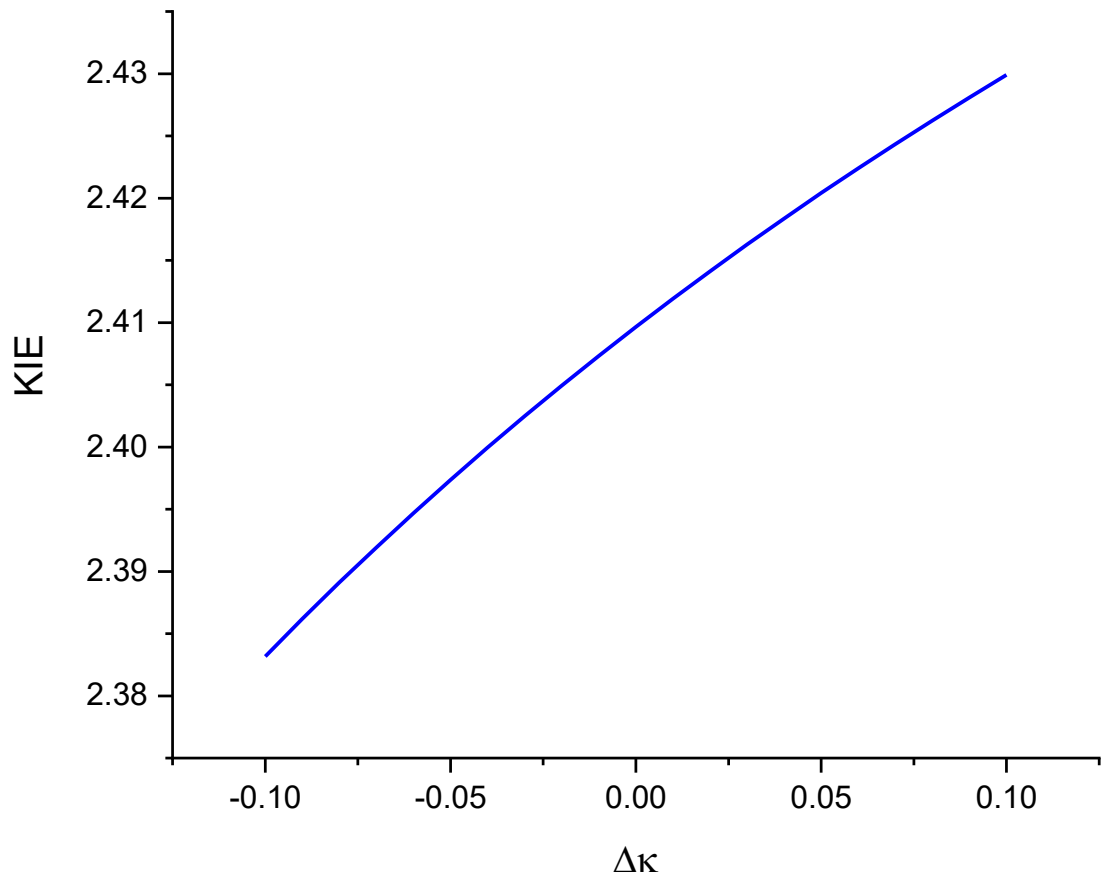

Figure S5. Influence of uniform variation of transmission coeficients on KIE.

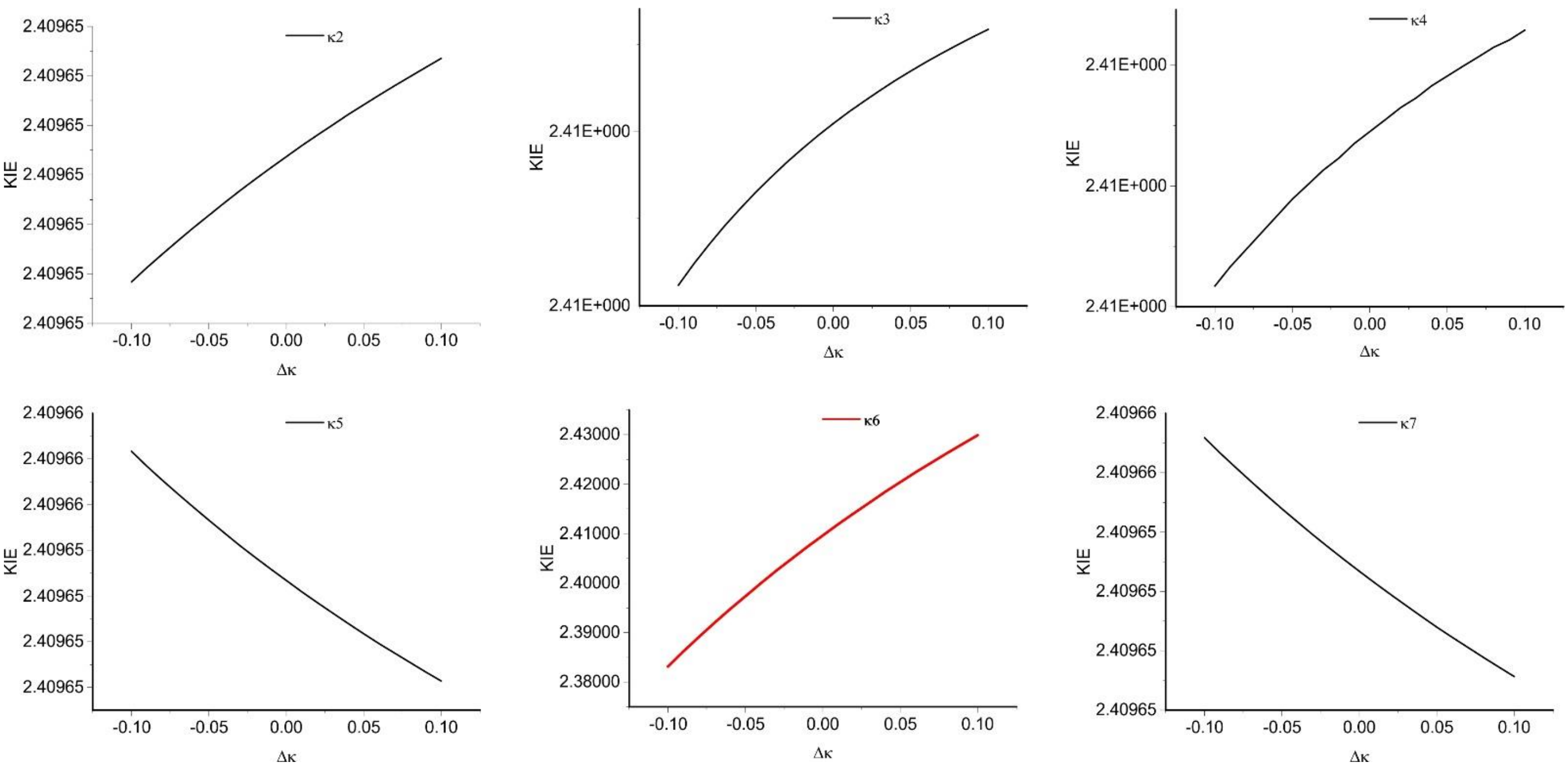

Figure S6. Influence of variation of individual transmission coefficients on KIE. The major influence is colored red.

H/D Exchange

Table S10. Kinetic constants and intrinsic kinetic isotope effects (iKIE) calculated for the H/D exchange of the *R*-benzylsuccinate for the highest level of theory and thermal energy corrections. Γ(T)*k_i provides values of kinetic constants with Wigner's correction for tunnelling.

	**$\Delta(E^{QM2}$ tzvp+Therm) [kJ/mol]**		**k [s^{-1}]**	**Γ(T)*k_i [s^{-1}]**	**iKIE**	**$\Delta(E^{QM2}$ tzvp+Therm) [kJ/mol]**		**k [s^{-1}]**	**Γ(T)*k_i [s^{-1}]**	**iKIE**
**I3**	0.00					17.7				
**$^{\alpha}d_1$-I3**	0.00					20.3				
**$^{\beta}d_1$-I3**	0.00					17.2				

**d_2-I3**	0.00					20.0				
**TS4$^{\alpha}$**	57.1	$^{\alpha}k_5$	917	2 324	1.0	74.8	$^{\alpha}k_{-5}$	97	2.1	1.0
**$^{\alpha}d_1$-TS4$^{\alpha}$**	59.7	$^{\alpha D}k_5$	326	611	2.8	80.0	$^{\alpha D}k_{-5}$	12	0.2	8.0
**$^{\beta}d_1$-TS4$^{\alpha}$**	62.5	$^{sec\text{-}\alpha}k_5$	104	262	8.8	73.9	$^{sec\text{-}\alpha}k_{-5}$	134	2.8	0.7
**d_2-TS4$^{\alpha}$**	59.3	$^{D2\alpha}k_5$	375	698	2.4	79.3	$^{D2\alpha}k_{-5}$	16	0.3	6.0
**EP**	-5.7					0.0				
**$^{\alpha}d_1$-EP**	-8.3					0.0				
**$^{\beta}d_1$-EP**	-5.2					0.0				
**d_2-EP**	-7.9					0.0				
**I3**	0.00					23.8				
**$^{\alpha}d_1$-I3**	0.00					26.1				
**$^{\beta}d_1$-I3**	0.00					24.6				
**d_2-I3**	0.00					27.0				
**TS4$^{\beta}$**	108.4	$^{\beta}k_5$	$1.3*10^{-6}$	$3.2*10^{-6}$	1.0	132.2	$^{\beta}k_{-5}$	$1.0*10^{-10}$	$2.6*10^{-10}$	1.0
**$^{\beta}d_1$-TS4$^{\beta}$**	111.0	$^{\beta D}k_5$	$4.6*10^{-7}$	$8.6*10^{-7}$	2.8	137.1	$^{\beta D}k_{-5}$	$1.5*10^{-11}$	$2.7*10^{-11}$	7.1
**$^{\alpha}d_1$-TS4$^{\beta}$**	107.9	$^{sec\text{-}\beta}k_5$	$1.6*10^{-6}$	$3.9*10^{-6}$	0.8	132.5	$^{sec\text{-}\beta}k_{-5}$	$9*10^{-11}$	$2.2*10^{-10}$	1.1
**d_2-TS4$^{\beta}$**	110.5	$^{D2\beta}k_5$	$6.E*10^{-7}$	$1.0E*10^{-6}$	2.3	137.6	$^{D2\beta}k_{-5}$	$1.2*10^{-11}$	$2.2*10^{-10}$	8.4
**E:P**	-23.8					0.0				
**$^{\alpha}d_1$-E:P**	-26.1					0.0				
**$^{\beta}d_1$-E:P**	-24.6					0.0				
**d_2-E:P**	-27.0					0.0				

**$k_{H/D}$**	$k_{HDX}*[D_2O]$= 5 s^{-1} M^{-1}*22.22 M=111 s^{-1}	$k_{H/D}$ ^{Tunel}	$k_{HDX}*[D_2O]$= 61.5 s^{-1} M^{-1}*22.22 M=1366.8 s^{-1}
**$k_{D/H}$**	$k_{HDX}*[H_2O]$= 5 s^{-1} M^{-1} *33.33 M= 167 s^{-1}	$k_{D/H}$ ^{Tunel}	$k_{HDX}*[H_2O]$= 5 s^{-1} M^{-1} *33.33 M= 167 s^{-1}

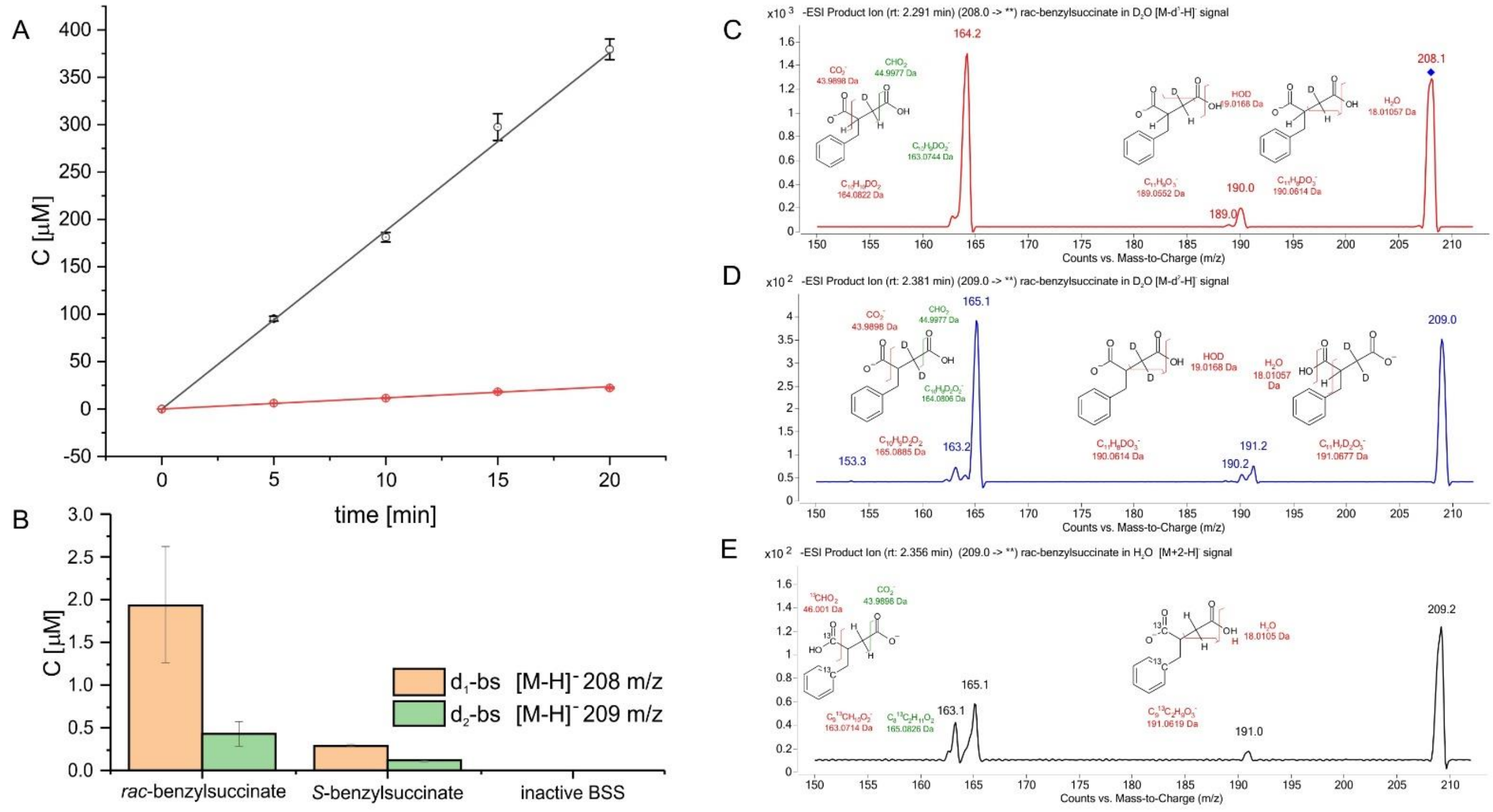

Figure S7. Results of the H/D exchange experiments. A) D/H exchange during reaction with d8-toluene in H2O, black line–formation of d_8-benzylsuccinate (215 m/z), red line formation of d_7-benzylsuccinate (214 m/z); B) yields of labeled products after 4h of benzylsuccinate incubation with BSS in D_2O, C) product ion spectrum of 208 m/z (mixture of d_1-benzylsuccinate and ^{13}C-benzylsuccinate) D) product ion spectrum of 209 m/z (mixture of d_2-benzylsuccinate, d_1-^{13}C2-benzylsuccinate and ^{13}C2-benzylsuccinate), E) product ion spectrum of 209 m/z from the assay with inactive BSS (fragment ions only from ^{13}C2-benzylsuccinate). Error bars–standard deviation. The figure reproduced from Szaleniec et al. [1]

Acknowledgements

The authors acknowledge the financial support provided by Deutsche Forschungsgemeinschaft/National Science Center Poland under Beethoven Life grant He2190/13-1 / 2018/31/F/NZ1/01856 as well as Polish high-performance computing infrastructure PLGrid (HPC Centre: ACK Cyfronet AGH) for providing computer facilities and support within computational grant no. PLG/2025/018050, PLG/2023/016888, PLG/2022/016024 and PLG/2021/015218. Authors acknowledge the joint consortium “Interdisciplinary Centre of Physical, Chemical and Biological Sciences” of ICSC PAS and INP PAS for providing access to the Agilent 1290 Infinity System with an automatic autosampler and an MS Agilent 6460 Triple Quad Detector. The sequence of the BSS encoding gene cluster was obtained from the whole-genome sequencing of *Aromatoleum* sp. The analysis was performed at the Genomics Core Facility in Malopolska Biotechnology Centre at Jagiellonian University, using short-read NGS and nanopore sequencing technologies.

References

1. Szaleniec, M.; Oleksy, G.; Sekuła, A.; Aleksić, I.; Pietras, R.; Sarewicz, M.; Krämer, K.; Pierik, A. J.; Heider, J., Modeling the Initiation Phase of the Catalytic Cycle in the Glycyl-Radical Enzyme Benzylsuccinate Synthase. *J. Phys. Chem. B* **2024,** *128* (24), 5823-5839.
